# Infection prevention requirements for the medical care of immunosuppressed patients: recommendations of the Commission for Hospital Hygiene and Infection Prevention (KRINKO) at the Robert Koch Institute 

**DOI:** 10.3205/dgkh000410

**Published:** 2022-04-13

**Authors:** Commission for Hospital Hygiene and Infection Prevention (KRINKO)

**Affiliations:** 1 Robert Koch Institute, Berlin, Germany

**Keywords:** immunocompromised, iInfection prevention and control, guideline, health care

## Abstract

In Germany, guidelines for hygiene in hospitals are given in form of recommendations by the Commission for Hospital Hygiene and Infection Prevention (Kommission für Krankenhaushygiene und Infektionsprävention, “KRINKO”). The KRINKO and its voluntary work are legitimized by the mandate according to § 23 of the Infection Protection Act (Infektionsschutzgesetz, “IfSG”).

The original German version of this document was published in February 2021 and has now been made available to the international professional public in English. The guideline provides recommendations on infection prevention and control for immunocompromised individuals in health care facilities. This recommendation addresses not only measures related to direct medical care of immunocompromised patients, but also management aspects such as surveillance, screening, antibiotic stewardship, and technical/structural aspects such as patient rooms, air quality, and special measures during renovations.

## Abstract (de)

In Deutschland werden die Anforderungen an die Hygiene im Gesundheitswesen in Form von Empfehlungen von der Kommission für Krankenhaushygiene und Infektionsprävention (KRINKO) veröffentlicht. Die KRINKO und ihre ehrenamtliche Arbeit legitimieren sich aus dem Auftrag nach §23 des Infektionsschutzgesetzes (IfSG).

Die deutsche Originalfassung dieses Dokuments wurde im Februar 2021 veröffentlicht und nun auf Englisch der internationalen Fachöffentlichkeit zur Verfügung gestellt. Das Dokument enthält Empfehlungen zur Infektionsprävention und -kontrolle bei der Versorgung von immungeschwächten Personen in Gesundheitseinrichtungen. Diese Empfehlung befasst sich nicht nur mit Maßnahmen, die die direkte medizinische Versorgung immungeschwächter Patienten betreffen, sondern auch mit Managementaspekten z.B. Überwachung, Screening, Antibiotic Stewardship, sowie mit technisch/strukturellen Aspekten z.B. Patientenzimmer, Luftqualität und besonderen Maßnahmen bei Renovierungsarbeiten.

## Legal notice

This translation is intended solely as a convenience to the non-German-reading public. Any discrepancies or differences that may arise in translation of the official German version of the recommendation of the Commission for Hospital Hygiene “Anforderungen an die Infektions-prävention bei der medizinischen Versorgung von immunsupprimierten Patienten” (Bundesgesundheitsbl. 2021;64(2):232–64, https://doi.org/10.1007/s00103-020-03265-x) are not binding and have no legal effect.

### Legal notice in German

**Rechtlicher Hinweis**

Rechtlich bindend ist die deutsche Originalfassung dieser Empfehlung „Anforderungen an die Infektionsprävention bei der medizinischen Versorgung von immunsupprimierten Patienten“ (Bundesgesundheitsbl 2021; 64:232–264, https://doi.org/10.1007/s00103-020-03265-x). Die englische Fassung dient der Information der internationalen Fachöffentlichkeit.

## List of abbreviations

- ABS: Antibiotic stewardship
- ADV: Adenovirus
- AFS: Antifungal stewardship
- AML: Acute myeloid leukaemia
- ART: Commission on Anti-infectives, Resistance and Therapy
- AWMF: Association of Scientific Medical Societies in Germany
- BMT: Bone marrow transplantation
- BSI: Bloodstream infection (infection with evidence of a pathogen in the blood culture, manifesting clinically as bacteraemia, fungaemia or sepsis) [1]
- CDI: *Clostridioides difficile* infection
- CHX: Chlorhexidine
- CMV: Cytomegalovirus
- CoNS: Coagulase-negative staphylococci
- CVAD: Central venous access device (an implanted central venous catheter providing long-term access, such as a Broviac or Hickman catheter or port)
- CVC: Central venous catheter
- Device: Medical device, the use of which is associated with an increased risk of infection (e.g. intravascular catheter, gastric tube, percutaneous endoscopic gastrostomy, tracheostomy, urethral catheter, bone implant materials, etc.)
- DIN: German Institute for Standardization
- ESBL: Extended-spectrum beta-lactamase
- FFP: Filtering face piece; respirator mask
- GVHD: Graft-versus-host disease
- HACCP: Hazard analysis and critical control points
- HD: Hygienic hand disinfection
- HEPA filter: High-efficiency particulate air/arrestance filter
- HHV: Human herpes virus
- HMPV: Human metapneumovirus
- HS dispenser: Hand sanitiser dispenser
- HSV: Herpes simplex virus
- IfSG: *Infektionsschutzgesetz*, German Infection Protection Act
- KISS: German Nosocomial Infection Surveillance System
- KRINKO: Commission for Hospital Hygiene and Infection Prevention at the Robert Koch Institute
- MRGN: Multidrug-resistant Gram-negative pathogens
- MRP: Multidrug-resistant pathogens
- MRSA: Methicillin (oxacillin)-resistant *S. aureus*
- NI: Nosocomial infections
- NRC: National reference centre
- NTM: Nontuberculous mycobacteria
- PBSCT: Peripheral blood stem cell transplantation
- PCR: Polymerase chain reaction
- PJP: *Pneumocystis jirovecii* pneumonia
- RKI: Robert Koch Institute
- RSV: Respiratory syncytial virus
- SCT: Stem cell transplantation (usually stem cells separated from peripheral blood)
- SM: Surgical mask
- STIKO: German Standing Committee on Vaccination
- TrinkwV: German Drinking Water Ordinance
- VDI: Association of German Engineers
- VRE: Vancomycin-resistant enterococci
- VZV: Varicella zoster virus
- WHO: World Health Organization

## Glossary of terms (as used in this document)

### Allogeneic stem cell transplantation

Transplantation of blood stem cells from another person, either a family member or an unrelated donor. 

### Autologous stem cell transplantation

Transplantation of the patient’s own blood stem cells, which are harvested from peripheral blood or bone marrow and processed, then frozen and transfused back into the patient at a later date.

### Bacteraemia

The presence of viable bacteria in blood; evidence of infectious bacteria in a properly collected blood culture.

### Basic hygiene measures

Measures taken in contact with all patients (or by patients themselves) to prevent transmission of infectious agents to patients and staff and to reduce the risk of nosocomial spread of pathogens. These include, in particular, hygienic hand disinfection (HD) and situational use of specific barrier measures:

- Disposable gloves if contamination of the hands with blood, respiratory secretions or other patient excretions is a possibility
- Protective clothing (patient-specific aprons or gowns) for tasks involving considerable contamination (e.g. caring for a patient with diarrhoea or vomiting)
- A respirator (e.g. FFP2 or FFP3) in the presence of patients with infections transmitted by aerosols
- A surgical mask (SM) for close contact with a patient who has an infection transmitted by droplets.

Other basic hygiene measures include the disinfection of contaminated surfaces and objects and the correct preparation of medical devices. Further information can be found in the KRINKO recommendations entitled “Infection Prevention in the Care and Treatment of Patients with Communicable Diseases [2]”. 

### Bloodstream infection

Evidence of an infectious agent in the properly collected blood culture of a patient with signs of infection, such as fever, and any other clinical or laboratory manifestations of systemic inflammatory response syndrome. The use of this term does not depend on the severity of the clinical picture.

### Facultative pathogens

Pathogens that require specific conditions to cause infectious diseases, such as access to parts of the body that are normally sterile, e.g. via catheter systems or foreign bodies, and can also cause infectious diseases in people who are not immunosuppressed.

### Graft-versus-host disease

Cells from the donor’s immune system recognise the recipient’s own antigens as foreign and cause an immune reaction that harms the recipient. The skin, mucous membranes and liver of the recipient (and the lungs in chronic GVHD) are most often affected. Intensification of treatment with immunosuppressants may be necessary to control GVHD, which has been assigned four grades of severity by the WHO.

### Severe graft-versus-host disease

In cases of severe GVHD, immunosuppression must be intensified, which substantially increases the risk of serious infections [3]. GVHD that meets one of the clinical international consensus criteria for grade 3 or 4 is considered to be severe [4].

### Induction therapy

In acute leukaemia, malignant cells crowd out healthy cells in the bone marrow, which can lead to infections and a tendency to bleed. In this situation, the primary goal of treatment is to destroy the diseased cells, enabling the displaced healthy cells to recover. To achieve this, several cycles of intensive chemotherapy are usually necessary. Chemotherapy administered to induce remission is called induction therapy, whereas chemotherapy given in remission is usually called consolidation therapy. Patients undergoing solid organ transplantation also require more intensive immunosuppression (e.g. with antithymocyte globulin or basiliximab) at the time of the transplant. This treatment is also called induction therapy and very important in terms of the level of immunosuppression in the individual patient.

### Isolation room

A room that can be used as a single room with ensuite sanitary facilities (shower and toilet), HS dispensers and an entrance area large enough for gowns, gloves and SM to be put on and disposed of before leaving the room [2].

### Neutropenia

A neutrophil count in peripheral blood of <0.5x10^9^/L or a white blood cell count that is <1x10^9^/L and falling if a differential blood count is not available. Severe (prolonged) neutropenia: neutropenia lasting longer than 10 days.

### Neutropenic diet

Explicitly avoiding any foods that can cause infections in immunosuppressed patients through contamination with and transmission of facultative or opportunistic microorganisms [5], [6], [7].

### Obligate pathogens

Pathogens which, in the absence of specific immunity, can cause infectious diseases in healthy people.

### Opportunistic pathogens

Pathogens which usually only cause infectious diseases if the immune system is compromised.

### Sepsis

Life-threatening organ dysfunction due to an inadequate host response to infections [1], [8], [9], [10].

### Severe immunosuppression

Severe immunosuppression equivalent or comparable to risk groups 2 and 3 (see Table 1 (Tab. 1)).

### Authors’ note on the English translation of some specific professional desig- nations in this document

This document uses professional terms for the hygiene team that are established in the German healthcare system. Since there may be no direct equivalents for these professional designations in other countries, we would like to explain the terms used in this document in more detail:

- *Infection control specialist* (“Krankenhaushygieniker”): In Germany, infection prevention and control is a certified medical specialty (“Facharzt für Krankenhaushygiene und Umweltmedizin”). As in other medical specialties, a 60-month postgraduate training must be completed after medical school to become an IPC specialist. This training includes 12 months spent in clinical wards (i.e., internal medicine, surgery, pediatrics) and at least 48 months in a certified and authorized IPC department [11].
- An *IPC link doctor* (“hygienebeauftragter Arzt”) is a physician working in the respective area who supports the implementation of the IPC measures in his or her area.
- An *IPC nurse* (“Hygienefachkraft”) is a nurse who has additional training in infection prevention and control.

## 1. Introduction and objectives

### 1.1. Background

Congenital and acquired forms of immunodeficiency are independent risk factors for potentially life-threatening nosocomial infections (NI), which can be caused by a multitude of pathogens (some opportunistic) [12]. The term immunosuppression is understood to mean the iatrogenic suppression of certain components of the immune system. The resulting immunodeficiency is either necessary for medical reasons (e.g. in certain autoimmune diseases or to prevent a rejection reaction after stem cell or organ transplantation) or a side effect of the medical treatment (e.g. cytostatic chemotherapy, radiotherapy or the use of biologicals in antineoplastic therapy). In this document, the term immunosuppression will also be used for patients (all references to professions or groups in this document include all genders) who are immunocompromised because of a congenital or acquired underlying disease and not medication [13].

Patients to whom these recommendations relate may be among those at risk of a complicated SARS-CoV-2 infection. Management of the pandemic caused by the new SARS-CoV-2 coronavirus is not covered by these recommendations. Please refer to relevant documents issued by the Robert Koch Institute (http://www.rki.de/covid-19) and competent medical professional associations, which are updated regularly, and to local pandemic plans.

### 1.2. Classification of risk groups

In its 2010 recommendations entitled “Hygiene Requirements for the Medical Care of Immunosuppressed Patients”, the Commission for Hospital Hygiene and Infection Prevention (KRINKO) defined three risk groups of immunosuppressed patients [14]. Infection prevention measures are based on the relevant risk group. This classification has been retained in this updated version (Table 1 (Tab. 1)). 

Further details of risk groups, infectious agents and transmission pathways can be found in Tab. 3 to Tab. 6 in Attachment 1. Tab. 3 contains information about infections that are occurring with greater frequency because of the increasing use of certain biologicals in recent years. As there are many possible patterns of findings, this table is inevitably incomplete. For instance, severe immunodeficiency can consist of immune cells which are numerically normal but dysfunctional. Important groups of patients with severe immunodeficiency include patients with certain congenital immunodeficiency syndromes, such as septic granulomatosis, and patients on immunosuppressive therapy (certain biologicals, long-term treatment with high doses of systemic corticosteroids or lifelong immunosuppression after organ transplantation). Acute treatment of rejection reactions after organ transplantation can be comparable with GVHD in terms of the resulting immunosuppression [15]. Tab. 4 provides a guide to the spectrum of pathogens causing invasive infections in patients with weakened immune systems.

**The risk groups of immunosuppressed patients defined here are a dynamic (and to some extent pragmatic) guide introduced primarily for adaptation of the required hygiene measures. This allocation concept suggested by the KRINKO must not be confused with other clinical risk scores or stages of disease.**

The specific situation of individual patients and the corresponding risk of infection can change in the course of treatment. Individual patients can move between risk groups depending on their clinical treatment situation (e.g. induction vs. consolidation therapy, recurrence of leukaemia, preparation for and execution of stem cell transplantation after conventional treatment). This means that it may be necessary for doctors to amend the risk group in their risk analysis.

A **“medical risk analysis”** is a critical review of the patients’ current situation (from an infection risk and prevention perspective) by the doctors treating them. It requires close on-site contact with the infection control specialist, the IPC link doctor and/or IPC nurse in hygiene so that more complex issues can be discussed at any time (please also refer to the KRINKO recommendations on personnel and organisational requirements for the prevention of nosocomial infections [16]).

As the attending physicians know from experience how long the neutropenia associated with certain underlying diseases and therapeutic interventions is likely to last, most patients can be assigned to the appropriate risk group in advance. Patients with solid tumours face additional risks because they usually require tumor surgery [17], [18], [19], [20], [21].

After allogeneic transplantation, the severity of graft-versus-host disease (GVHD) is also particularly crucial to the intensity of immunosuppressive therapy and corresponding risk group assignment. Patients with severe GVHD of the skin or gastrointestinal tract are at particularly high risk of severe exogenous and endogenous infections.

### 1.3. Prevention aims of these recommendations

The **overall aim of these updated recommendations** is to reduce the incidence of NI in immunosuppressed patients, if possible to unavoidable events [22], thereby increasing patient safety, improving their quality of life [23] and reducing the morbidity and mortality associated with NI [12], [24], [25]. It is also to counteract the selection and transmission of pathogens with specific and multidrug resistance, and NI caused by *Clostridioides (C.) difficile* (CDI) or viral pathogens, by taking appropriate measures.

### 1.4. Target groups of these recommendations

These recommendations are aimed at all professionals who are directly or indirectly involved in the medical care of immunosuppressed patients, particularly doctors and the relevant medical professional associations, nursing staff, hygiene professionals (infection control specialists, IPC link doctors, IPC nurses, physiotherapists, technical staff, public health services, hospital administrative staff, doctors working for health insurance companies, medical students and trainees [e.g. in healthcare and nursing]). They are also intended to provide a basic framework for infection prevention when drawing up plans for new wards and specialist outpatient clinics in which severely immunosuppressed patients will be treated.

### 1.5. What is new in these recommendations?

These recommendations replace the 2010 recommendations entitled “Hygiene Requirements for the Medical Care of Immunosuppressed Patients”. To make them easier to follow, this revised version has a new structure. Instead of a detailed introductory analysis of the various causes and manifestations of immunodeficiency (or immunosuppression), reference is made to relevant specialist literature [12], [26], [27], [28], [29], [30], [31],[32]. The KRINKO assumes that those responsible for the medical treatment of immunosuppressed patients have a sufficient knowledge in line with their training. Some recommendations are preceded by specific background information, which is intentionally brief so the document is easier to read.

This revised and updated document focuses on specific recommendations for NI prevention in healthcare facilities treating immunosuppressed patients [30]. Section 5 of the 2010 recommendations, which contains guidance on infection prevention during periods of outpatient treatment, is to be transferred to the information booklet first produced in 2010 by the German Association of Self-Help Organisations for Patients with Leukaemia and internet-based resources for wider distribution and use [33].

The KRINKO evidence categories from 2010 [34] are used in these recommendations (Table 2 (Tab. 2)). As there is no scientific evidence from controlled studies for some of the recommendations, not every recommendation is assigned a category.

There are of course many direct references to the latest version of other KRINKO recommendations, which are listed in the references section. The primary purpose of these recommendations is to add to existing KRINKO recommendations by highlighting specific aspects of the medical treatment of this particular patient population. However, very important measures that also feature in other KRINKO recommendations will be repeated in places. 

## 2. Recommendations

### 2.1. Prevention

#### 2.1.1. Training for all staff

**The KRINKO recommends:**

- Regular training in nosocomial infection prevention and control **in immunosuppressed patients** for all members of the treatment team (no cat.). This involves the transfer of knowledge and specific practical skills in accordance with locally agreed standards.
- That critical activities, such as the care of intravascular catheters and other devices, only be performed independently by adequately trained staff [35] (cat. IV).
- Combining training with practical exercises in small groups using examples from everyday clinical practice and involving hygiene professionals (no cat.).

#### 2.1.2. Training for patients and their relatives

Many patients and those accompanying them want to be actively involved in infection prevention through general information, specific advice and practical guidance [36], [37], [38], [39]. Severely immunosuppressed patients are often admitted to hospital, in some cases for long periods of time, or attend specialist outpatient clinics or day hospitals for their treatment. In such cases, there is a continuity of interaction with medical staff, which can be used to share basic hygiene strategies and repeatedly emphasise their importance. For example, without continuous active guidance, hygienic hand disinfection (HD) is performed too rarely by patients and visitors, but together with hand sanitiser dispensers (HS dispensers) (e.g. at the entrance to the hospital, ward or specialist outpatient clinic), it can increase the HD rate of patients and visitors [40], [41], [42], [43]. Experience shows that nursing staff are tremendously important in providing patients and their relatives with information and guidance about basic hygiene measures because of the intensive contact they have with them. Patients who are consistently asked to take these measures by medical staff are more likely to tell them about gaps in prevention (e.g. staff HD or three-way valve disinfection before manipulation of a central venous catheter) [42], [44], [45]. Obstacles arising from language barriers or a lack of health knowledge can be anticipated and overcome by involving patients and providing practical guidance. A professional translation should be provided if possible. Precise and clear explanations and rules should be communicated in simple terms. Patients who already have neutropenia or are expected to develop it in the course of their treatment should be told what neutropenia is and why it increases the risk of infections. Patients must know how fever is defined, how to take their temperature and exactly what to do if they develop a fever. Specific information and rules (on all aspects of this section) are essential for appropriate behaviour during periods of outpatient treatment [46], [47], [48]. Contradictory statements from different members of the treatment team should be avoided. Any existing differences of opinion within the team should not be shared with the patient; speak with one voice where possible [30], [49], [50].

**The KRINKO recommends:**

- Actively involving patients (and relatives, visitors and companions) as partners in infection prevention and control as much as possible (no cat.). This requires well-planned and sustained efforts by the entire treatment team [27], [50].
- Emphasising the importance of the hands in the transmission of pathogens on first contact after diagnosis [51], [52], [53] (cat. IB).
- Explaining and demonstrating the most important indications and correct procedure for HD (and hand washing at home) [54], [55], [56], [57] (see KRINKO recommendations entitled “Hand Hygiene in Healthcare Facilities” [54], [55] [no cat.]).
- At a later stage of treatment, specifically addressing more complex aspects of infection prevention, e.g. strategies for preventing food-related infections [58], [59], [60] or, if appropriate, infections transmitted through contact with pets or farm animals (case history) [61], [62], [63], and which vaccinations are recommended for relatives or close contacts [64] (cat. II).
- Where they are used: explaining why certain measures that go beyond basic hygiene [e.g. wearing a surgical mask (SM), contact isolation, protective isolation] are required and what they consist of (see sections 2.1.9 and 2.1.13) (no cat.). Experience shows that this avoids conflict and improves adherence.

If patients or the relatives caring for them are involved in any aspect of their treatment, **the KRINKO recommends:**

- Training them to the same standard as everyone else in the department, as happens with new members of the treatment team (no cat.).
- Documenting this training carefully (no cat.).

For example, this can include central venous catheter (implanted for long-term access) maintenance care during periods of outpatient treatment [65], [66], [67], [68], [69]. Patients (or their relatives) should be trained to carry out critical aspects of catheter care (such as dressing changes or flushing the catheter) independently [70], [71], [72]. 

These standards must never overwhelm patients or their relatives. Such training is not possible or wise for all patients (families).

To support the provision of information and active patient involvement, the KRINKO recommends:

- As a team (with hygiene professionals), discussing the most important issues and deciding exactly how to communicate them (no cat.).
- For example, providing brochures or simple handouts on basic hygiene and other subjects, in the patient’s language where possible, and using pictograms or visual aids [43] (no cat.).
- Involving patient representatives in the development of new information materials where possible (no cat.).
- Referring to reliable (for example, provided or reviewed by medical professional associations) online resources for patients [33], [73], [74], [75] (no cat.).

#### 2.1.3. Visitor rule requirements

Even with immunosuppressed patients in an inpatient setting, it is in their interests to actively encourage and facilitate social contact with relatives and visitors in order to counteract social isolation, depression and a tendency to withdraw. However, visitors must not have a communicable infectious disease (or be in the incubation period after known exposure to such a disease) and should always take basic hygiene measures when in contact with patients. The more open the communication between the treatment team, patients and relatives about this, the more likely patients and relatives (e.g. parents) are to ask in advance whether or not a visit is appropriate in terms of a possible infection. Basic clinical screening of all children for signs of infection before they enter the ward can be included in visitor rules but has not been shown to help prevent infection. If relatives and patients have understood how to behave and learnt to ask the treatment team any outstanding questions, such screening is not absolutely necessary outside risk groups 2 and 3 (see Table 1 (Tab. 1)). Infants who have received a live rotavirus vaccine should not have close contact with severely immunosuppressed patients for the following two weeks (if contact is unavoidable: disposable gloves during and HD after nappy changes).

**The KRINKO recommends:**

- Establishing binding rules for visitors, which can be consulted immediately in individual cases, in consultation with the most senior doctor and nurse in the ward or department (no cat.).
- Instructing visitors in HD and, if necessary, other aspects of basic hygiene and infection prevention (see KRINKO recommendations entitled “Hand Hygiene in Healthcare Facilities” [54], [55]) (no cat.).
- Excluding visitors who have a potentially transmissible infection (cardinal symptoms include fever, heavy cold, cough, conjunctivitis, unexplained rash, diarrhoea or vomiting) (no cat.).
- That visitors with signs of a mild respiratory infection, such as rhinitis, or oral herpes wear a SM (in addition to strict HD) [2], [76] (cat. II).
- Excluding visitors with only mild symptoms of a respiratory infection from visiting patients in risk groups 2 and 3 (see Table 1 (Tab. 1)) (no cat.).
- Also instructing children in HD in a way that is appropriate to their age and stage of development. Direct supervision and manual assistance are usually required until they reach school age (no cat.).

For the following measures, there is no scientific evidence that they help to prevent infection. **Therefore, the KRINKO does not recommend them (cat. III) in risk group 3 **[77]** (except in appropriate treatment situations or for specific infection epidemiology reasons in the general population)**

- Gowns for all visitors
- A SM for all visitors (exception: see 2.1.5.2; current prevention measures for the COVID-19 pandemic are of course unaffected by this)
- Disposable gloves for all visitors [78].

#### 2.1.4. Immunoprophylaxis

Active and passive immunisation of immunosuppressed patients is the subject of current recommendations by the German Standing Committee on Vaccination (STIKO) at the Robert Koch Institute, the body mandated by the IfSG to provide such recommendations [64], [79], [80], and medical professional associations [81]. Employers in the health service are entitled to know the vaccination status of their employees and use this information *“to make decisions about a new employment relationship or the nature of employment”*. As some easily transmitted, vaccine-preventable illnesses can be life-threatening, especially in immunosuppressed or immunocompromised patients (e.g. measles, chickenpox, influenza), the most senior doctor and nurse, infection control specialist and the occupational medical service should work intensively together to ensure that, where possible, all non-immune members of the treatment team get vaccinated against these illnesses [82], [83], [84]. This is an important task for the hospital administration [85]. Irrespective of this, please read section 20 (8) of the IfSG (duty to demonstrate immunity to measles), which came into effect on 01.03.2020 [84]. 

**The KRINKO recommends:**

- That treating physicians play an active role in ensuring that patients (depending on their current treatment situation and degree of immunosuppression) and their relatives (close contacts) are fully vaccinated according to the appropiate STIKO recommendations (no cat.).
- That medical staff (all types) who work in close contact with immunosuppressed patients are fully vaccinated, particularly to prevent nosocomial infections (including the annual influenza vaccine) [86], [87], [88], [89], [90], [91] (cat. IB).

If staff who have not been vaccinated against influenza have close patient contact (contact and droplet infection), the hygiene committee should review additional measures to minimise the risk of transmission (e.g. wearing a SM in addition to HD and other basic hygiene measures) (no cat.).

#### 2.1.5. Basic hygiene measures

##### 2.1.5.1 Hand hygiene

Hygienic hand disinfection (HD) is the most important measure in the prevention of NI. Without appropriate HD, staff hands have been shown to be contaminated with pathogenic microorganisms [92].

Adherence to HD is often relatively high on wards containing immunosuppressed patients [93], [94], [95]. However, even on these wards, staff disinfect their hands more often after patient contact than before (e.g. before an aseptic activity [95]). In a study of a stem cell transplantation unit, authorisation to disinfect gloved hands increased adherence, particularly before aseptic activities. The incidence density of severe infections (in this case: bloodstream infections [healthcare-associated bloodstream infection; HABSI] and pneumonia [hospital-acquired pneumonia; HAP]) decreased (from 6.0 to 2.5/1,000 patient days, not statistically significant), and the transmission rate of multidrug-resistant pathogens (MRP) (MRSA, ESBL-producing Gram-negative pathogens, VRE) was unaffected [96]. In order to maintain a high standard, participation in the *“Aktion Saubere Hände”* **Clean Hands Campaign** (or local implementation of a comparable concept) seems eminently reasonable [56]. HS dispensers should also be installed in the entrances to the relevant wards and outpatient clinics so that the first HD can take place on arrival [40], [41], [42].

**The KRINKO recommends:**

- Regular careful instruction, training and supervision of the entire treatment team in following the KRINKO recommendations on hand hygiene (cat. IA/IB, see KRINKO recommendations entitled “Hand Hygiene in Healthcare Facilities” [54], [55]).
- Installing an adequate number of patient-accessible HS dispensers in wards and specialist outpatient clinics for immunosuppressed patients (cat. IA/IB, see KRINKO recommendations entitled “Hand Hygiene in Healthcare Facilities” [54], [55]).

##### 2.1.5.2 Patient-specific protective clothing and scrubs

**The KRINKO recommends:**

- In order to contain certain transmissible infectious agents, wearing suitable, exclusively patient-specific protective clothing (e.g. aprons, gowns) for activities in which close patient contact could contaminate work clothing with blood, faeces, urine or secretions, and in general when caring for patients with diarrhoea, vomiting or extensive wounds (in this context, specific reference is made to the KRINKO recommendations entitled “Infection Prevention in the Care and Treatment of Patients with Communicable Diseases” [2], [97]) (no cat.).
- That medical staff providing care wear work clothes (not their private clothes) that have been properly prepared by their employer [2] (no cat.).
- That staff and relatives also use patient-specific protective gowns for close contact with patients in risk group 3 (see Table 1 (Tab. 1)) and therefore protective isolation [77] (cat. II).
- Using SMs for protective isolation in risk group 3 (see Table 1 (Tab. 1)) and for targeted prevention of droplet infections (no cat.).

Staff who have an acute infection should not work in close contact with immunosuppressed patients [98]. It is important to remember that respiratory viruses, which are generally harmless and self-limiting in otherwise healthy people, can cause a potentially life-threatening infection in immunosuppressed patients [99], [100], [101]. After infection with respiratory viral pathogens, immunosuppressed patients shed these pathogens for a much longer period without symptoms [102], [103], [104], and therefore measures to prevent transmission (contact and droplet) are sometimes required for several weeks.

When there is a clear seasonal increase in the incidence of respiratory infections in the general population (e.g. a recent increase in the rate of inpatient admissions for influenza or respiratory syncytial virus infection (RSV) [105], [106], [107], [108], [109]) and during the acute phase after stem cell transplantation, it can be beneficial for the treatment team and visitors to wear SM (during all contact with the patient) [104], [110], [111]. The same applies in the oncology outpatient clinic and waiting room [112]. For a situationally appropriate and flexible approach to the treatment team and visitors wearing a SM for all close patient contact, close cooperation and coordination with hospital hygiene and the attending diagnostic laboratory (current number of confirmed cases of influenza or RSV in the hospital as a whole) is crucial. 

#### 2.1.6. Antiseptic full body washes

In recent years, a full body wash (“bathing”) with solutions containing chlorhexidine (CHX) or octenidine, or with pre-packaged washcloths, has been promoted as a basic infection prevention measure, particularly in intensive care units and before and after surgery [113]. As well as to prevent NI (e.g. bloodstream infections and postoperative wound infections), the aim is to reduce the probability of transmitting certain multidrug-resistant pathogens [114], [115], [116]. According to a recent survey by the European Society for Blood and Marrow Transplantation (EBMT), 31% of the 109 participating stem cell transplantation centres do this systematically [117]. Other than for MRSA decolonisation [118], the KRINKO has previously only recommended this measure for cases in which the treating doctors and infection control specialists consider other preventive measures to be insufficient [65], [66], [67], [119], [120]. Existing studies of the use of a full body wash in immunosuppressed patients are not sufficient for a clear recommendation [113], [121], [122]. In the commonly cited study by Climo et al., only one stem cell transplantation department participated, and its results are not presented separately (only in Fig. 2 with no statistical data) [123]. This study is also the focus of criticism because of a possible conflict of interests [124]. In the full body “wash” patient group, the incidence density of intravascular catheter-related infections caused by coagulase-negative staphylococci (CoNS), in particular, was reduced; this can also be achieved through consistent use of prevention bundles [65], [66], [67], [120]. During the study, the investigational product (CHX washcloths) was withdrawn from the market temporarily because it was contaminated with *Burkholderia* *cepacia*. The inefficacy of CHX against certain Gram-negative infectious agents can promote outbreaks of the corresponding infections [125]. Octenidine, the antiseptic used in Germany as an alternative, also exhibits gaps in efficacy here [126], [127], [128]. 

In the ABATE study by Huang et al., 4,730 and 5,800 oncology patients (not including acute stem cell transplantation) were included in the control and CHX full body bathing groups, respectively. There were no significant differences in the primary endpoints (clinical cultures for MRSA or VRE and the incidence of bloodstream infections). Oncology patients with a central venous catheter were probably in the subgroup of patients with devices for whom the post-hoc analysis showed a benefit. However, it remains unclear whether restricting CHX “bathing” to oncology patients with a central venous catheter (CVC) has any advantage as alternative to the design of the ABATE study [116]. Certain device-related infections such as bacteriuria and candiduria are less common in men with a urinary catheter [129] and other patients who receive a CHX full body wash [130]. This may be because CHX treatment included all skin defects and wounds as well as the first 15 cm of every catheter (e.g., the ABATE study instruction video https://vimeo.com/164608558 [116] and section 2.1.1). The use of CHX full body treatments can reduce the CHX susceptibility of Gram-positive infectious agents in the corresponding patient population *in vitro* [131], [132]. The clinical significance of this observation remains unclear.

As there is still insufficient evidence for immunosuppressed patients, the KRINKO can neither recommend nor dismiss the use of an antiseptic full body wash (cat. III).

#### 2.1.7. Cleaning and disinfection

Please refer to the current version of the KRINKO recommendations entitled “Hygiene Requirements for the Cleaning and Disinfection of Surfaces” [133] and “Infection Prevention in the Care of Patients with Communicable Diseases” [2], [97], the pathogen-specific KRINKO recommendations entitled “Recommendations for the Prevention and Control of Methicillin-resistant Strains of *Staphylococcus aureus* (MRSA) in Medical and Care Facilities”, “Hygiene Measures for the Prevention of Infections Caused by Enterococci with Specific Antibiotic Resistance”, “Hygiene Measures for Infections or Colonisation with Multidrug-resistant Gram-negative Bacteria” and “Hygiene Measures for *Clostridioides difficile* Infection (CDI)” [118], [134], [135], [136] as well as recent secondary literature on this subject [137], [138], [139].

**The KRINKO recommends:**

- incorporating the above requirements for the cleaning and disinfection of surfaces into an overarching quality management plan that has been reviewed and approved by the infection control specialist or member of staff responsible for hygiene standards [140], [141] (no cat.).
- providing cleaning staff for an appropriate number of hours with evidence of specific training, who can understand and follow instructions from the treatment team immediately, (no cat.). This is necessary to meet the high and sometimes rapidly changing demands of such high-risk areas of inpatient care adequately.

#### 2.1.8. Number and features of isolation rooms

**The KRINKO recommends:**

- in view of the overall significantly increased and increasing demand for isolation rooms on a ward containing severely immunosuppressed patients [142], [143], [144], [145], [146], [147], [148], [149], [150], [151], equipping at least 50% of the rooms so they can be used for isolation: a room that can be used as a single room with ensuite sanitary facilities (shower and toilet), HS dispensers and an entrance area large enough for gowns, gloves and SM to be put on and disposed of before leaving the room [2] (cat. II).

#### 2.1.9. Protective isolation

**The KRINKO recommends:**

- accommodating neutropenic patients in risk groups 1 and 2 (Table 1 (Tab. 1)) in a single or twin room with ensuite sanitary facilities, but not larger units (three or more patients per room), and carefully observing basic hygiene measures (no cat.). As paediatric haemato-oncology patients are regularly admitted with a parent (companion caregiver), their rooms should be large enough for a folding bed to be set up next to the bed without excessively obstructing their care (particularly at night) or creating additional transmission risks.
- accommodating patients in risk group 3 (see Table 1 (Tab. 1)) in a single room with ensuite sanitary facilities [77] (for room air requirements, see section 2.1.13) (no cat.).

#### 2.1.10. Isolation in the event of colonisation or infection with transmissible pathogens

The treatment team should take active steps to counteract the negative effects of single-room isolation on the quality of medical and psychosocial treatment by providing adequate staffing and, where appropriate, monitoring devices (central monitors, intercom systems), facilitating external psychosocial support and making electronic forms of entertainment and communication (internet, etc.) available at the bedside [152]. No patient should receive lower-quality medical monitoring or treatment as a result of colonisation or infection or the need for protective isolation. For MRGN, VRE and MRSA, please refer to the KRINKO recommendations entitled “Hygiene Measures for Infections or Colonisation with Multidrug-resistant Gram-negative Bacteria”, “Hygiene Measures for the Prevention of Infections Caused by Enterococci with Specific Antibiotic Resistance” and “Recommendations for the Prevention and Control of Methicillin-resistant Strains of *Staphylococcus aureus* (MRSA) in Medical and Care Facilities” [118], [134], [135], [153].

Biehl et al. conducted a prospective study in the haemato-oncology departments of four German university hospitals [93]. They investigated the prevalence of colonisation with 3MRGN (resistance to third-generation cephalosporins and (for 3MRGN) also to fluoroquinolones. [Translator’s note: In the German MRGN classification system, an isolate is classified as 3MRGN if it is resistant to 3 of the 4 groups of antibiotics (piperacillin, third-generation cephalosporins, carbapenems and fluoroquinolones)]) *E. coli* on admission and the nosocomial transmission and development of bacteraemia resulting from this colonisation. Two hospitals were compared in each case. Patients with 3MRGN *E. coli* colonisation were treated in a single room where possible in two of the hospitals but not in the other two. In this patient population, which generally had high previous antibiotic exposure, the prevalence of 3MRGN on admission was 7.7% and 7.5%, respectively. The proportion of patients with 3MRGN colonisation was therefore slightly higher in haemato-oncology than in studies that included all university hospital admissions [93], [154], [155]. In this study, both nosocomial transmissions and bloodstream infections due to 3MRGN *E. coli* were extremely rare (overall 1.59% without isolation and 1.01% with isolation) and not significantly influenced by single-room isolation [93]. In a systematic literature review, the same research team also found a low incidence of secondary bloodstream infections (BSI) after colonisation with ESBL-producing Enterobacteriaceae [156]. However, an Italian multicentre study found higher BSI rates in colonised haemato-oncology patients (15.6% for ESBL producers and 14.1% for carbapenem-resistant Gram-negative pathogens [157]).

**The KRINKO recommends:**

- After a medical risk analysis, isolating patients who are infected with infectious agents or shedding asymptomatically (colonised) in a room that can be used as a single room in accordance with written hygiene standards based on the transmission route of the relevant pathogen (please refer to the KRINKO recommendations entitled “Infection Prevention in the Care and Treatment of Patients with Communicable Diseases”, “Recommendations for the Prevention and Control of Methicillin-resistant Strains of *Staphylococcus aureus* (MRSA) in Medical and Care Facilities”, “Hygiene Measures for the Prevention of Infections Caused by Enterococci with Specific Antibiotic Resistance”, “Hygiene Measures for Infections or Colonisation with Multidrug-resistant Gram-negative Bacteria” and “Hygiene Measures for *Clostridioides difficile* Infection (CDI)”) [2] [97], [118], [134], [135], [136].
- That single-room isolation is not always necessary for adult haemato-oncology patients who are colonised (or infected) with **3MRGN *E.coli*** and able to follow basic hygiene measures consistently (for 3MRGN isolates of other species, there are no comparable studies and therefore contact isolation in high-risk areas is still recommended) [93] (cat. II).
- That before cohorting risk group 2 patients (see Table 1 (Tab. 1)) with the same pathogen, a medical risk analysis be performed to check whether other aspects of infection prevention rule out cohorting for individual patients (no cat.).
- That in departments specialising in the treatment of immunocompromised patients, there is at least one room that is separated from the rest of the ward by an anteroom (with two doors) and has a ventilation system (negative pressure in the anteroom with sufficient airflow) that enables patients with airborne infectious diseases (e.g. chickenpox, measles) to be isolated [2] (no cat.). Such patients can also be accommodated in a designated isolation ward at the same hospital if this ward can provide the same quality of medical monitoring and treatment for the underlying disease (no cat.).
- Not giving patients (or their close contacts) who are isolated for a communicable disease or asymptomatic shedding of a transmissible pathogen (colonisation) free access to communal areas (e.g. the ward kitchen) (no cat.).
- That parents/close contacts admitted with paediatric patients also isolate with the child [158]. This is particularly important for patients with MRSA, VRE, 3MRGN or 4MRGN colonisation (no cat.).

Close contacts isolated with the patient should pay particular attention to basic hygiene measures within the isolation room (HD, protective gown when providing *care*, e.g. washing, and clean disposable gloves when changing nappies, etc.). Hands should be disinfected before leaving the room. In some hospitals, it has proven beneficial for these parents to wear a gown (and SM, depending on the pathogen) *outside the isolation room* if they are not leaving the ward immediately.

#### 2.1.11. Prevention of infections transmitted by contaminated foods

There is a fundamental distinction between:

1. An outpatient care setting (patients eat at home or outside the healthcare facility), where basic food hygiene rules apply. Immunosuppressed patients are familiar with these rules, which should be strictly observed by patients and their relatives [58], [59], [60]. Patients and their families require specific and structured advice here (see section 2.1.2 and see Tab. 5 in Attachment 1).
2. The hospital kitchen, which must meet special requirements (statutory regulations and controls) based on HACCP principles [159]. Infection prevention measures during food production, storage and distribution by the hospital operator (“hospital food”) apply to all patient groups and are not included in these recommendations.
3. Food storage and preparation in a **ward kitchen that is accessible to patients and relatives** (in paediatric haemato-oncology: “parents’ kitchen”). During intensive treatment, it can be difficult to feed immunosuppressed patients adequately and thereby prevent cachexia and other complications [160], [161], [162], [163] [164]. In this context, for medical reasons, it may be necessary to give patients access to their own food in addition or as an alternative to hospital food during their inpatient stay. This is sometimes achieved by bringing in food or by preparing and storing food for individual patients in a ward kitchen.
4. The storage and delivery of food administered via a tube (gastric tube, jejunal tube, percutaneous endoscopic gastrostomy), formula milk (infants) [165], [166], [167], [168] and breast milk (if this is expressed and stored).

Of course, ward kitchens used by patients and their relatives also require a hygiene plan that has been agreed with hygiene professionals and is binding for all users of the kitchen. 

**The KRINKO recommends:**

- continuing to avoid certain foods with a high risk of pathogenic bacterial contamination after discharge from hospital (see Tab. 5 in Attachment 1) and paying particular attention to basic hygiene measures when buying, storing and preparing food (see Tab. 5 in Attachment 1) [5] (no cat.).

The KRINKO is strongly opposed to a strict “neutropenic diet”, as the benefits are unproven and such a diet can significantly reduce the patient’s quality of life [7], [77], [169], [170], [171], [172], [173], [174], [175], [176], [177], [178] (cat. II).

Certain probiotics (of which standardisation under the German Medicinal Products Act is a significant problem) may have a favourable effect on the microbiome of immunosuppressed patients (e.g. a decrease in the incidence of antibiotic-associated diarrhoea or CDI) [179], [180], [181], [182], [183], [184]. In a study of microbiome analyses, no significant change after administration of probiotics was demonstrated [185]. However, analyses of various patient populations, individual case reports and case series suggest that probiotic microorganisms very rarely cause systemic (bloodstream) infections [185], [186], [187], [188], [189], [190], [191], [192], [193], [194], [195], [196], [197], [198], [199], [200], [201], [202].

**The KRINKO recommends:**

- for immunosuppressed patients in risk groups 2 and 3, carefully weighing up the risk of using **probiotics** (or approving their use by declaring that probiotic supplements are safe) against the expected benefit [203], [204] (cat. III).

#### 2.1.12. Structural functional measures to ensure a protective environment

**The KRINKO recommends:**

- That wards and specialist outpatient clinics treating patients in our risk categories 1–3 may not be a passage to reach other wards or outpatient clinics but instead form separate structural units (no cat.).
- That all surfaces, including the floor, be easy to clean and disinfect [133] (no cat.). Upholstered furniture, carpets and potted plants are not suitable [138].
- That environmental contamination by water spray from washbasins be avoided with a splash guard where necessary. This is particularly important in intervention rooms and areas in which injections, infusions, medication and enteral feeding solutions are prepared [205] (cat. II).

#### 2.1.13. Room air requirements

**The KRINKO recommends:**

- That in order to avoid invasive aspergillosis/filamentous fungal infections, patients undergoing induction therapy for AML (or relapsed AML) and patients in the acute phase after allogeneic stem cell transplantation or with severe GVHD stay in state-of-the-art rooms supplied with HEPA-filtered air (filter class H13) during their inpatient treatment [77], [206], [207], [208] (cat. IB). After autologous stem cell transplantation (without additional immunosuppression to prevent GVHD, which does not occur) patients do not require an isolation room with HEPA-filtered air, as in most centres the incidence of invasive mould infections is under 5% [209], [210] and the use of rooms with HEPA-filtered air does not have a significant effect on the rate of nosocomial pneumonia [211].

That is why, in many centres, it has not been compulsory to treat autologous stem cell transplantation patients in isolation rooms with HEPA-filtered air for a number of years [212], [213], [214]. It is important to remember that patients may already have asymptomatic fungal colonisation of the respiratory tract or paranasal sinuses on admission, which will not be affected by the provision of HEPA 13-filtered room air. In addition to the above measures, medication to prevent fungal infections caused by Aspergillus in high-risk patients is a crucial component of the overall prevention plan for invasive mycosis. However, this is not covered by these recommendations.

**The KRINKO also recommends:**

- Providing suitable protective isolation rooms with an anteroom (air pressure in the patient room is positive to the anteroom, air pressure in the anteroom is negative to the patient room and corridor, mainly to maintain safe pressure differentials between the patient room and corridor, and to prevent positive pressure in the patient room carrying pathogens into the corridor via exhaust air) [214]. However, there are no clinical studies that confirm the benefit of an anteroom for the endpoint of NI or NI transmission (no cat.).
- With new buildings or major renovations, giving consideration to providing HEPA-filtered air, not only in individual patient rooms but also entire wards or certain sections of wards (at least two-stage F9 filtration in corridors), so that even severely immunosuppressed patients can move around freely, thereby reducing their risk of social isolation (no cat.).
- With new buildings or major rebuilding work, not installing laminar air flow/low-turbulence displacement flow in isolation rooms, as there is no scientific evidence that this helps to prevent infection [77] (no cat.).
- Ensuring that all ventilation systems are regularly inspected and maintained in accordance with technical specifications (DIN 1946-4) and submitting the results of the hygiene acceptance test and regular state-of-the-art hygiene tests to the infection control specialist [215] (no cat.).
- That after an individual risk assessment, patients in risk group 2 or 3 wear a tight-fitting particle-filtering respirator (FFP2) when they leave their room [216], [217] (cat. II).
- Not using humidifiers or other technical equipment that emits potentially contaminated aerosols or raises dust (fans; justified exception: relieving dyspnoea during palliative treatment) (no cat.).
- If there is a ventilation system, not opening the windows where possible. It should not be possible for patients or staff to open patient room windows, except in a fire emergency. This requires appropriate state-of-the-art climate control and ventilation (see DIN 1946-4 and VDI 6022) [215], [218] (no cat.).
- Not setting up composting or waste processing units near departments in which severely immunosuppressed patients are treated, as they can emit large quantities of fungal spores [219] (cat. II).
- Not using leaf blowers to clear leaves in the immediate vicinity of departments in which severely immunosuppressed patients are treated; a safety zone should be defined here (no cat.).

#### 2.1.14. Requirements relating to the water supply and sanitary facilities

This section provides a number of basic recommendations for the prevention and control of water-related infections, which are particularly important for immunosuppressed patients [220], [221], [222], [223], [224]. Please refer specifically to the recommendations in “Hygiene Requirements for Wastewater Systems in Medical Facilities” for more information on this subject. In particular, the measures described in the appendix for areas with a higher risk of infection should be considered for risk groups 2 and 3.

##### 2.1.14.1 Hygienic construction and use of washbasins, showers and toilets in medical areas

These include [225]:

- considering the use of thermic siphon disinfection devices in high-risk areas containing group 2 and 3 patients provided the risk of retrograde contamination or aerosol formation cannot be controlled by other technical means [222],
- in the area surrounding the washbasin, providing an area (or storage space) that is protected from water spray, where patients can store personal care items (toothbrushes, creams, etc. and dressings) of relevance for transmission,
- every room having ensuite sanitary facilities with a washbasin, shower and toilet (sanitary facilities shared by no more than two patients in risk groups 1 and 2, one sanitary area per patient in risk group 3),
- washbasin taps that can be operated without using the hands. As taps with an electronic sensor may increase the risk of water contamination, their use is only justifiable with careful microbiological monitoring [226],
- not directing the water jet into the drain or having an overflow [55], [227],
- adequate ventilation of sanitary facilities so they do not become a reservoir for moulds and other pathogens [228],
- choosing materials that can be cleaned using suitable disinfection methods (per-compounds, chlorine compounds) for the drains of washbasins, showers and toilets,
- specifying how often shower tubes should be replaced (e.g. every six months),
- not using shower curtains because these are laborious to disinfect [229], [230], [231], [232],
- closing the toilet lid before flushing in order to avoid contaminating the surrounding area and user with spray and aerosols. The toilet should always be flushed with the lid closed before use.
- rimless toilets [222].

##### 2.1.14.2 Supply of drinking water (or mineral water from unopened bottles) 

**The KRINKO recommends:**

- If the microbiological quality of the water is not guaranteed by other means [233], using terminal bacterial filters in haemato-oncology wards and other wards treating severely immunosuppressed patients, particularly in patient rooms [224], [227], [234], [235], [236], [237], [238] (cat. II). It is important to ensure that external contamination of the filters does not result in transmission of the pathogens that its use is intended to prevent [239] (cat. II).
- **Not** **giving patients in risk groups 2 and 3** (see Table 1 (Tab. 1)) **still mineral water** in hospital (even for oral hygiene), as still mineral water can be contaminated with bacteria [240]. Instead, carbonated, sterile-filtered or boiled drinking water, or alternatively a drinking fountain with a sterile filter, is recommended (no cat.).
- When making tea (for drinking or oral hygiene), not simply bringing water to a boil but instead leaving it on a rapid boil for several minutes [241], as tea leaves can be contaminated with pathogenic bacteria and fungi (cat. II). A critical analysis of the medical use of tea in patient care should be undertaken for this reason.

#### 2.1.15. Hygiene requirements for demolition and reconstruction work

Building work in hospitals responsible for the inpatient care of severely immunosuppressed patients, or nearby construction or demolition work, can increase patient exposure to infectious agents [242]. This exposure can cause life-threatening infections (e.g. invasive aspergillosis of the respiratory tract) [243]. However, it is possible to avoid this kind of critical exposure through strict implementation of a suitably adapted bundle of measures ensuring close cooperation between clinicians, hospital hygiene, building designers and the companies carrying out the work [244], [245]. Antifungal prophylaxis to prevent invasive fungal infection does not provide adequate protection for severely immunosuppressed patients on its own [243]. Before any major building work takes place, the treating doctors should consider reviewing the indications in the guidelines for medication to prevent invasive fungal infections [246], [247], [248], [249], [250] in their own patient populations (*not within the responsibility of KRINKO*).

**The KRINKO recommends:**

- Without exception, agreeing all building, renovation and demolition work in the vicinity of severely immunosuppressed patients with the appropriate hygiene professional (infection control specialist) and the most senior nurse and doctor in the relevant department as early as the planning phase and otherwise immediately [251], [252], [253], [254] (cat. II). Hospital administration, or a subunit of hospital administration in charge of building work, is responsible for informing hospital hygiene staff about such work, giving a reasonable period of advance notice, and involving infection prevention personnel in the process.
- In contracts with the planners and companies doing the work, explicitly agreeing that the prevention guidelines of the hospital will be strictly followed by their employees and that these guidelines are a non-negotiable part of the building contract (no cat.).
- For larger projects that are likely to mean greater exposure for immunosuppressed patients, forming a multidisciplinary prevention group coordinated by infection prevention personnel well in advance of the work starting. This group makes specific recommendations on the protective measures that are required and monitors the building work from a hospital hygiene perspective on behalf of the medical and administrative director (no cat.).
- If necessary to protect patients, completely or temporarily transferring the relevant ward from the area of risk into another building [254], [255] until the work has been completed (cat. II).
- If there is no central ventilation system with terminal HEPA filtration, and mainly to avoid temporarily high exposure caused by building and renovation work, considering the use of decentralised mobile HEPA filtration units in patient rooms [256], [257], [258] (cat. II).
- Informing patients during building work and before major building or renovation work of a potentially increased risk of invasive fungal infections [242], [251], [252], [254], [259], [260], [261] in a suitable way (no cat.).
- That, where possible, immunosuppressed patients avoid areas where building work is taking place, and wear an FFP2 respirator with an exhalation valve during transport through such areas (where this is unavoidable) [27], [48] (cat. II).
- Safely shielding the ward from building work and preventing secondary entry of dust and dirt with a predetermined route. Where conditions allow, an impermeable dust barrier can often be created using drywall. To check for perfect sealing, a visual inspection should be performed (e.g. with gas detector/air flow test tubes) and the results documented [261] (no cat.).
- The removal of building rubble, the exhaust air system and disposal methods must be specified. Sealed containers should be used where necessary (no cat.).
- Cleaning patient care areas that are exposed to large amounts of construction dust with a wet disinfectant at least every working day and when dust is visible (no cat.).
- On wards and in outpatient clinics in the immediate vicinity of activities that generate dust, not opening windows while work is ongoing and protecting sterile materials and consumables from contamination (no cat.).
- After any work on the drinking water system with the potential to cause stagnation or contamination, or if it has not been used for a long time, checking the water **for compliance with** **TrinkwV** and for *Legionella* and *P. aeruginosa* before patients are exposed to it (cat. IV).
- Carrying out targeted surveillance of invasive mould infections while construction work is ongoing [242], [245], [261] (no cat.).

#### 2.1.16. Prevention of nosocomial urinary tract infections

Please refer to the KRINKO recommendations entitled “Prevention and Control of Catheter-related Urinary Tract Infections” [262].

#### 2.1.17. Prevention of postoperative wound infections 

As there is nothing specific to consider in terms of prevention strategy, please refer to the KRINKO recommendations entitled “Prevention of Postoperative Wound Infections” [263] and the secondary literature [17], [18], [19], [20], [21], [264].

#### 2.1.18. Prevention of infections originating in intravascular catheters

In addition to the KRINKO recommendations entitled “Prevention of Infections Originating in Intravascular Catheters” [65], [66], [67], [120], please refer to the detailed recommendations on preventing catheter-related BSI published by professional associations in the fields of general medicine and paediatric haemato-oncology [68], [69]. 

A significant proportion of BSI in patients with mucositis and/or severe GVHD after intensive chemotherapy do not originate in intravascular catheters [265], [266]. Nevertheless, severely immunosuppressed patients who require a central venous catheter for their treatment belong to the risk groups [265], [267], [268], [269], [270], [271], [272] for which the use of specific aids (e.g. CHX-releasing dressings, disinfection caps) can be considered after a medical risk analysis [65], [66], [67], [120], [273], [274], [275], [276], [277].

#### 2.1.19. Prevention of oral infections

**The KRINKO recommends:**

- That a dental consultation be arranged for all newly admitted patients and, depending on the clinical situation, starting any dental treatment that may be required in order to minimise the risk of local inflammation and systemic infections [278], [279], [280] (cat. II).
- Instructing patients in regular oral and dental hygiene in accordance with an in-house standard agreed by an interdisciplinary team, which can be continued during phases of oral (pharyngeal) mucositis [281], [282], [283], [284] (cat. IB).
- General (non-targeted) use of mouthwash solutions containing CHX is not recommended in this context [285] (cat. IB).

#### 2.1.20. Prevention of nosocomial infections of the gastrointestinal tract

Immunosuppressed patients can develop intestinal infections caused by opportunistic pathogens and shed these pathogens for a relatively long period of time, even after the acute symptoms have passed [286], [287], [288]. On the other hand, only a third of all episodes of diarrhoea in immunosuppressed patients after stem cell or organ transplantation are caused by a gastrointestinal infection [136], [289], [290]. In this context, targeted and rational pathogen diagnostics are important, including for hospital hygiene reasons [291]. 

**The KRINKO recommends:**

- To rule out nosocomial infections in immunosuppressed patients with diarrhoea, considering prompt advanced pathogen diagnostics, the specifics of which should be established with the relevant microbiologists, virologists and hygiene professional (no cat.).
- Depending on the pathogen (and on patient adherence to targeted prevention measures), follow-up tests to confirm the duration of isolation after symptoms of infectious diarrhoea have subsided because immunosuppressed patients may continue shedding the pathogen for much longer [292], [293], [294], [295], [296], [297] (cat. II). This does not include *C. difficile*-associated infections (a follow-up test is not required for asymptomatic patients) [136], [295], [298], [299], [300], [301].

Patients undergoing intensive chemotherapy or who have had a stem cell transplant are among those with the highest incidence density of CDI [299], [302], [303], [304], [305], [306]. For information on prevention, please refer to the latest KRINKO recommendations entitled “Hygiene Measures for *Clostridioides* *difficile* Infection (CDI)” [136].

#### 2.1.21. Prevention of zoonotic diseases

**The KRINKO recommends:**

- That risk group 2 and 3 patients (see Table 1 (Tab. 1)) in protective isolation have no contact with animals in hospital [61], [62], [63], [307] (cat. II).

A less restrictive approach may of course be taken with palliative care patients [308], [309], whereby contact with animals is restricted to these patients. Outside the hospital, simple rules of basic hygiene (e.g. washing hands with soap, HD) should be followed, as these can significantly reduce the risk of zoonotic diseases when handling pets and farm animals [61], [62], [63], [310], [311], [312], [313], [314], [315], [316] (see Tab. 6 and Compilation 1 in Attachment 1).

### 2.2. Surveillance

Please refer to the latest version of the KRINKO recommendations entitled “Nosocomial Infection Surveillance” [317]. First and foremost, surveillance data should be used in house to reduce the NI incidence rate on a long-term basis, or keep it as low as possible, in the interests of patient safety [317], [318], [319], [320]. External communication of particularly low infection rates is not the aim of NI surveillance [318], [321], [322]. There has not yet been a conclusive study depicting the definite proportion of avoidable NI in immunosuppressed patients [22], [318]. In a survey of 109 European Society for Blood and Marrow Transplantation (EBMT) centres published in 2015, only 21% carried out prospective surveillance of catheter-related bloodstream infections (CRBSI) [117]. KRINKO encourages the relevant medical professional associations to work with the National Reference Centre for the Surveillance of Nosocomial Infections on the continuous development of existing data acquisition modules for NI in immunosuppressed patients, increasing active participation among centres and including the use of anti-infectives [93].

**The KRINKO recommends:**

- That haemato-oncology treatment centres carry out prospective surveillance of nosocomial infections (particularly bloodstream infections; the RKI currently only recommends such surveillance in association with the use of intravascular catheters. For severely immunosuppressed patients, however, surveillance includes other BSI (see definition B3 in ONKO-KISS [haemato-oncology component of the nosocomial infection surveillance system]).) as set out in the German Infection Protection Act (Infektionschutzgesetz, IfSG) [323] and corresponding comments by the RKI [324], [325], [326], [327], [328], [329], [330] (cat. IB). Without surveillance, it is impossible to confirm whether prevention measures taken in the department are effective and have a beneficial effect on certain indicator infections in the long term (e.g. prevention bundles for bloodstream infections and CDI).
- Regularly reporting NI surveillance results along with pathogen and resistance statistics for invasive infections back to the treatment team, discussing these results with them and, if necessary, specifying further infection prevention measures (cat. IV).
- Carrying out NI surveillance using definitions that have been adapted to the characteristics of the immunocompromised patient population [330], [331], [332] (cat. II).

This includes consideration of neutropenia (incidence and duration) as the most established risk factor [327]. During cytostatic chemotherapy, however, a substantial proportion of all NI occur when the patient is not neutropenic. It is therefore sensible to include data on BSI and CDI, for example, when patients are not neutropenic [330]. In these patient groups, the ONKO-KISS module (patient-based surveillance after stem cell transplantation; https://www.nrz-hygiene.de/surveillance/kiss/onko-kiss/) and the STATIONS-KISS module (ward- or department-based surveillance of device-related infections; https://www.nrz-hygiene.de/surveillance/kiss/stations-kiss/; category: haemato-oncology) developed by the NRC are particularly suitable for NI surveillance. In general, the aim should be for hygiene professionals and haemato-oncologists to reach agreement [333], [334], [335] about whether a BSI is a secondary event (after translocation from the gastrointestinal tract in cases of severe mucositis or GVHD, for example) [265], [267], [268], [269], [270], [271], [272], [336], [337].

**The KRINKO recommends:**

- That NI caused by *Legionella* *pneumophila* or *Clostridioides* *difficile* and probable/confirmed invasive fungal infections (and NI caused by pathogens with specific or multidrug resistance) also be recorded for patients who are not neutropenic [338] (cat. IV, multidrug-resistant pathogens must be recorded pursuant to section 23 of the IfSG [339]).
- That haemato-oncology treatment centres are allocated an appropriate number of hours for surveillance by hygiene professionals [16] (no cat.).

### 2.3. Microbiological screening of immunosuppressed patients

Immunosuppressed patients often have comorbidities, a history of intensive contact with the healthcare system and in some cases extensive cumulative exposure to antibiotics (including certain reserve antibiotics [93]). However, the prevalence of colonisation with multidrug-resistant bacteria (e.g. MRSA, VRE, MRGN) can vary considerably in the different subgroups of immunosuppressed patients [340], [341], [342], [343], [344], and the consequences of such colonisation for individual patients cannot be summarised in a common risk algorithm [345]. In this respect, medical risk analysis and local epidemiology of infections caused by the relevant pathogens are particularly useful when deciding whether screening for certain MRP on admission helps to prevent infections [118], [134], [135], [142], [143], [144], [148], [346], [347], [348], [349], [350], [351], [352], [353], [354], [355]. For patients who come from countries with an increased incidence of tuberculosis or have stayed in these countries in the last year, tuberculosis should be included in the differential diagnosis of cough, fever and enlarged mediastinal lymph nodes for infection prevention reasons [356], [357].

**The KRINKO recommends:**

- Instead of general and undifferentiated MRP screening in immunosuppressed patients, preparing a local screening plan for colonisation with certain multidrug-resistant pathogens in consultation with hygiene professionals and the attending microbiology laboratory (cat. II). The KRINKO recommendations entitled “Recommendations for the Prevention and Control of Methicillin-resistant Strains of *Staphylococcus aureus* (MRSA) in Medical and Care Facilities” [118], “Hygiene Measures for the Prevention of Infections Caused by Enterococci with Specific Antibiotic Resistance” [134] and “Hygiene Measures for Infections or Colonisation with Multidrug-resistant Gram-negative Bacteria” [135], [358], the medical risk analysis (patient population) and local multidrug-resistant pathogen epidemiology are the starting point for this.
- When interpreting pathogen and resistance statistics, paying special attention to infectious agents that show resistance to the antibiotics or antifungals used to prevent infection in the department [246], [359], [360], [361], [362], [363], [364], [365], [366], [367], [368] (cat. II).
- Outside the agreed screening indications, refraining from routine microbiological cultures of patients and the environment if there is no suspicion of an infection or outbreak because random environmental controls do not have any measurable benefit [369], [370], [371], [372], [373] (cat. IB); this excludes those prescribed by quality assurance laws and ordinances.
- Particularly in the winter months (November to April; influenza and RSV season), in addition to prompt diagnostic tests for symptomatic patients, considering screening patients in risk group 3 (see Table 1 (Tab. 1)) for influenza and RSV on admission, for example with (RT-)PCR tests, because this makes it easier to identify immunocompromised patients who are already infectious but not (yet) symptomatic and has many implications for individual patients and hospital hygiene [100], [101], [102], [103], [104], [112], [374], [375], [376], [377], [378], [379] (cat. II).
- In immunosuppressed patients with signs of atypical pneumonia, regular testing for *Legionella* (e.g. PCR testing of respiratory secretions in addition to the urinary antigen test) and, in the event of a positive result, informing hospital hygiene immediately, as even one nosocomial *Legionella* infection can indicate a nosocomial source of infection [234], [380] (cat. II).

### 2.4. Antimicrobial stewardship in immunosuppressed patients

This falls within the responsibilities of the Commission on Anti-infectives, Resistance and Therapy (ART) afilliated with the Robert Koch Institute and medical professional associations [381], [382]. The general information given here has been agreed with the ART. 

Fundamentally, the hospital hygiene, infection prevention and **antimicrobial stewardship (ABS)** programmes followed in clinical practice have common goals that focus on patient protection (safety) and a continuous improvement in the quality of treatment [142], [143], [144], [383], [384], [385], [386], [387], [388]. This is particularly true for the prevention, control and clinical management of infections caused by pathogens with specific or multidrug resistance. In immunosuppressed patients, such pathogens are particularly important when they cause clinically severe infections (e.g. sepsis). In such cases, it can be crucial for the spectrum of efficacy of empirical antibiotic treatment to include pathogens with specific resistance [142], [143], [144], [148], [342], [346], [347], [348], [349], [350], [351], [361], [362], [388], [389], [390], [391], [392], [393], [394], [395], [396], [397], [398], [399], [400], [401], [402], [403], [404], [405], [406], [407], [408], [409], [410], [411], [412], [413], [414]. On the other hand, this must not lead to prolonged uncritical use of reserve antibiotics [156], [415], [416], [417], [418], [419], [420], [421]. In haemato-oncology, professional associations have already developed comprehensive evidence-based guidelines for diagnosis and treatment in certain clinical situations involving infectious diseases, on which a local interdisciplinary ABS programme can be based. Immunosuppressed patients with a history indicating penicillin intolerance (but not clear immediate anaphylactic reactions) must undergo further evaluation to rule out a (very rare) penicillin allergy of the immediate type so as not to be unjustifiably denied the most effective treatment with penicillins or other beta-lactam antibiotics for their indication [422], [423], [424], [425], [426], [427], [428].

The prevention and treatment of invasive fungal infections in certain high-risk patients is another broad area in which enhanced **antifungal** **stewardship** **(AFS)** initiatives are now being developed with the help of national and international professional association guidelines [429], [430], [431], [432], [433], [434], [435], [436], [437], [438], [439], [440], [441], [442], [443], [444], [445]. 

Furthermore, staff specialising in hospital hygiene and infection prevention (and doctors with responsibility for hygiene) are increasingly taking advantage of ABS training opportunities [446], and therefore synergies exist in the personalisation of corresponding programmes (see Compilation 2 in Attachment 1).

**The KRINKO recommends (with the agreement of the Commission on ART):**

- That the medical professional associations in the AWMF continue with the progressive development and detailed formulation of existing guidelines on antimicrobial stewardship [381], [382] for the different clinical areas in which immunosuppressed patients are treated so that the characteristics of this patient population are given proper consideration [148], [270], [351], [408], [447], [448], [449], [450], [451], [452], [453], [454], [455], [456], [457], [458], [459], [460], [461], [462], [463], [464], [465], [466] (cat. II). The challenge is to identify distinct targets of ABS and AFS that are as specific as possible [467] (see Compilation 2 in Attachment 1) and implement sustainable ABS/AFS strategies in clinical practice [468].
- When updating guidelines on the diagnosis and treatment of certain infections in immunosuppressed patients, focusing on aspects of ABS and AFS from a critical perspective and including them (no cat.).
- Developing common indicators for the quality of hospital hygiene and infection prevention structures, processes and results and for ABS/AFS programmes in immunosuppressed patients [469], [470], [471], [472], [473] (cat. II).
- Particularly in hospitals treating risk group 2 and 3 patients (see Table 1 (Tab. 1)), setting up ABS programmes that meet medical professional association standards (AWMF guidelines) [382] (cat. II). This also applies to paediatric haemato-oncology treatment centres and paediatric organ transplantation centres [381] (cat. II).

These recommendations were produced on behalf of the Commission for Hospital Hygiene and Infection Prevention by Prof. Heike von Baum, Dr. Peter Bischoff, Prof. Dr. Maximilian Christopeit, Prof. Steffen Engelhart, Prof. Martin Exner, Prof. Thomas Lehrnbecher and Prof. Arne Simon (Head of the Working Party) on a voluntary basis and without influence from commercial groups. From the Robert Koch Institute, Dr. Eva Feuerhahn and Dr. Melanie Brunke were involved. The recommendations were prepared by the working party and, after a detailed discussion, agreed by the Commission.

## Notes

### Competing interests

The author declares to have no competing interests.

## Supplementary Material

Tab. 3–6; Compilation 1–2

## Figures and Tables

**Table 1:**
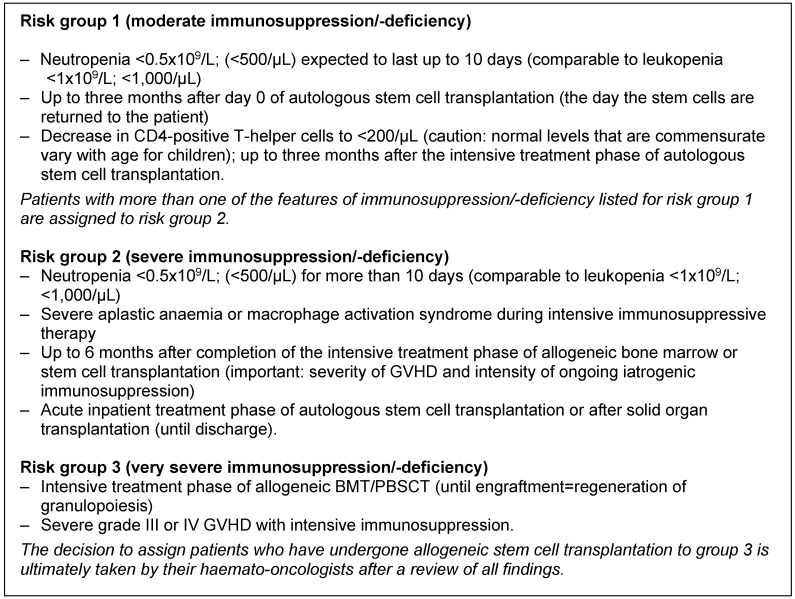
Risk groups (see notes in the text, dynamic concept)

**Table 2:**
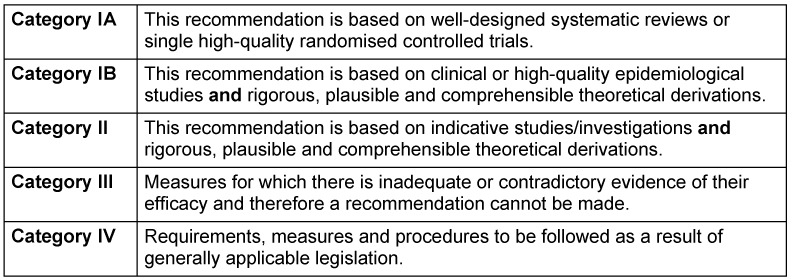
Categories in the Hospital Hygiene and Infection Prevention Guidelines (2010) [34]
