# Supplementary material for: Infection prevention requirements for the medical care of immunosuppressed patients: recommendations of the Commission for Hospital Hygiene and Infection Prevention (KRINKO) at the Robert Koch Institute
Source: GMS Hyg Infect Control. 2022 Apr 13;17:Doc07. doi: 10.3205/dgkh000410 (PMC9174886; doi:10.3205/dgkh000410)
Supplement: Tab. 3–6; Compilation 1–2 [file HIC-17-07-s-001.pdf]

Attachment

Table 3: Associations between certain immunosuppressive/antineoplastic active substances and their target structures and a selection of infectious complications [474], [475], [476], [477], [478]

Target molecule (active substances*)	Specific infectious complications
TNF-α (infliximab, adalimumab, golimumab, certolizumab pegol, etanercept)	• Reactivation of latent tuberculosis<br>• Reactivation of other granulomatous infections<br>• Reactivation of chronic hepatitis B<br>• Increased risk of severe and potentially fatal fungal infections
IL-1α, IL-1β (anakinra, canakinumab, gevokizumab, rilonacept)	• General increase in the risk of infection<br>• Theoretically increased risk of reactivating latent tuberculosis
IL-5 (mepolizumab, reslizumab)	• No association with specific infections reported to date
IL-6 (tocilizumab, siltuximab)	• General increase in the risk of infection<br>• Reactivation of VZV<br>• Reactivation of chronic hepatitis B<br>• Reactivation of latent tuberculosis
IL-12, IL-23 (common p40 subunit, ustekinumab, risankizumab, guselkumab)	• Slightly increased risk of reactivating VZV<br>• Slightly increased risk of reactivating chronic hepatitis B<br>• Theoretically increased risk of reactivating latent tuberculosis<br>• Tinea infections (tinea pedis, tinea cruris, tinea corporis, pityriasis versicolor, tinea manuum, onychomycosis) with risankizumab
IL-17 (secukinumab, ixekizumab, brodalumab)	• Slightly increased risk of mucocutaneous candidiasis
IgE (omalizumab)	• Possibly increased risk of parasitic infections (geohelminths, case reports)
Complement component C5 (eculizumab, ravulizumab)	• Infections with<br>o *Neisseria meningitidis*<br>o *Neisseria gonorrhoeae*<br>• Other bacterial infections, including *Streptococcus pneumoniae* and *Haemophilus influenzae B*
VEGF-A, VEGF-B, PIGF (bevacizumab, panitumumab, aflibercept)	• General increase in the risk of infection, most likely due to neutropenia<br>• Increased risk of gastrointestinal perforation with secondary peritonitis and bacteraemia
VEGFR-2, tyrosine kinase domain of VEGFR (ramucirumab, sorafenib, sunitinib, axitinib, pazopanib, regorafenib, vandetanib, cabozantinib)	• Only ramucirumab; like VEGF-A and VEGF-B
EGFR/HER1 (cetuximab, panitumumab)	• General increase in the risk of infection, most likely due to neutropenia<br>• Superinfection of the papulopustular rash
ErbB2/HER2 (trastuzumab, trastuzumab emtansine, pertuzumab)	• No association with specific infections reported to date
Tyrosine kinase domains of EGFR/HER1, ErbB2/HER2 and other members of the ErbB family (erlotinib, gefitinib, afatinib, neratinib, lapatinib, osimertinib)	• No association with specific infections reported to date
BCR-ABL, c-KIT, others (imatinib, dasatinib, nilotinib, bosutinib, ponatinib)	• Invasive fungal infections<br>• Reactivation of VZV<br>• Reactivation of latent tuberculosis<br>• CMV reactivation/infection (particularly dasatinib)
Ras/Raf/MEK/ERK (vemurafenib, dabrafenib, encorafenib, trametinib, cobimetinib, selumetinib)	• No association with specific infections reported to date

Target molecule (active substances*)	Specific infectious complications
Bruton's tyrosine kinase (ibrutinib, acalabrutinib)	• *Pneumocystis jirovecii* pneumonia<br>• Invasive fungal infections<br>• Progressive multifocal leukoencephalopathy (ibrutinib)
Ras/PI 3-kinase/Akt/mTOR (idelalisib, buparlisib, rigosertib, duvelisib)	• *Pneumocystis jirovecii* pneumonia<br>• Invasive fungal infections<br>• CMV reactivation/infection
BCL2 (venetoclax)	• No association with specific infections reported to date
JAK/STAT (ruxolitinib, tofacitinib, baricitinib)	• *Pneumocystis jirovecii* pneumonia<br>• Reactivation of VZV<br>• Reactivation of latent tuberculosis<br>• CMV reactivation/infection<br>• EBV reactivation/infection<br>• Progressive multifocal leukoencephalopathy
Ras/PI 3-kinase/Akt/mTOR (sirolimus, everolimus, temsirolimus)	• Reactivation of VZV<br>• Reactivation of latent tuberculosis
Histone deacetylase inhibitors (vorinostat, panobinostat, romidepsin)	• No association with specific infections reported to date
CD19 (blinatumomab)	• Reactivation of HSV<br>• Reactivation of VZV<br>• *Pneumocystis jirovecii* pneumonia<br>• Reactivation of chronic hepatitis B
CD20 (rituximab, obinutuzumab, ofatumumab, veltuzumab, ocrelizumab)	• Neutropenia risk<br>• Possibly *Pneumocystis jirovecii* pneumonia<br>• Reactivation of chronic hepatitis B<br>• Reactivation of hepatitis C<br>• Enteroviral infections<br>• Progressive multifocal leukoencephalopathy
CD52 (alemtuzumab)	• Reactivation of HSV<br>• Reactivation of VZV<br>• Reactivation of Epstein-Barr virus<br>• Reactivation of chronic hepatitis B<br>• At higher doses for indications other than multiple sclerosis also *Pneumocystis jirovecii* pneumonia, invasive fungal infections, reactivation of JC and BK polyomavirus, neutropenia<br>• At lower doses for multiple sclerosis also human papillomavirus, reactivation of latent tuberculosis, listeriosis and candidiasis
CD22 (epratuzumab, inotuzumab ozogamicin)	• No association with specific infections reported to date
CD30 (brentuximab vedotin)	• Neutropenia<br>• Reactivation of chronic hepatitis B<br>• CMV reactivation/infection<br>• Progressive multifocal leukoencephalopathy
CD33 (gemtuzumab ozogamicin)	• No association with specific infections reported to date
CD38 (daratumumab)	• Neutropenia<br>• Reactivation of VZV
CD40 (dacetuzumab)	• Neutropenia<br>• Possibly infections comparable with those occurring in hyper IgM syndrome (*Pneumocystis jirovecii* pneumonia, CMV reactivation/infection, invasive fungal infections, protozoa)
CD319 (elotuzumab)	• Reactivation of VZV

Target molecule (active substances*)	Specific infectious complications
CCR4 (mogamulizumab)	• Possibly neutropenia<br>• Reactivation of chronic hepatitis B<br>• CMV reactivation/infection
CTLA-4 (ipilimumab, abatacept)	• Reactivation of latent tuberculosis<br>• Listeriosis<br>• Secondary infections caused by supportive treatment with corticosteroids: *Pneumocystis jirovecii* pneumonia, invasive aspergillosis
PD-1, PDL-1 (nivolumab, pembrolizumab, atezolizumab)	• Reactivation of latent tuberculosis<br>• Listeriosis<br>• Secondary infections caused by supportive treatment with corticosteroids: *Pneumocystis jirovecii* pneumonia, invasive aspergillosis
LFA-3/ CD2 (alefacept)	• No association with specific infections reported to date
Integrins (natalizumab, vedolizumab)	• Progressive multifocal leukoencephalopathy (natalizumab)<br>• *Clostridioides difficile* (vedolizumab)
Sphingosine-1-phosphate receptor (fingolimod, siponimod, ozanimod)	• Reactivation of VZV<br>• Cryptococcal meningitis<br>• Progressive multifocal leukoencephalopathy<br>• Human papillomavirus (ozanimod)
Proteasome inhibitor (bortezomib, carfilzomib, ixazomib)	• Reactivation of VZV<br>• Respiratory illnesses<br>• Complications after influenza

*Active substances are grouped together for methodological reasons. There may be significant differences in the risk of specific infectious complications between individual active substances in these groups.

Compiled by: Prof. Dr. Maximilian Christopeit, Department of General Medicine II – Haematology, Oncology, Clinical Immunology and Rheumatology, Tübingen University Hospital and previously

Interdisciplinary Inpatient and Outpatient Department of Stem Cell Transplantation, Hamburg Eppendorf University Hospital (UKE)

Table 4: Pathogens causing invasive infections in immunocompromised patients

**Neutropenia**	• Coagulase-negative staphylococci (CoNS, e.g. *Staphylococcus epidermidis*)<br>• *Staphylococcus aureus*<br>• α-haemolytic (viridans) streptococci (e.g. *Streptococcus mitis*)<br>• *Enterococcus faecium*<br>• Enterobacteriaceae (*Escherichia coli, Enterobacter* spp.*, Klebsiella oxytoca, Klebsiella pneumoniae, Proteus* spp.*, Serratia marcescens)*<br>• Non-fermenters (*Achromobacter xylosoxidans, Acinetobacter baumannii, Burkholderia cepacia, Pseudomonas aeruginosa, Ralstonia pickettii, Stenotrophomonas maltophilia)*<br>• *Legionella* spp.<br>• Fungi (*Aspergillus* and *Candida* spp., Mucoraceae)
**T-cell defect**	• Mycobacteria (particularly *Mycobacterium tuberculosis* but also nontuberculous mycobacteria (NTM) such as *Mycobacterium avium, Mycobacterium fortuitum, Mycobacterium marinum, Mycobacterium septicum,* etc.)<br>• *Listeria monocytogenes* and *Nocardia* spp.<br>• Viruses (CMV, HSV, VZV, HHV-6, RSV, HMPV, ADV, polyomaviruses, e.g. BK virus)<br>• Fungi (and *Cryptococcus* spp., *Pneumocystis jirovecii*) [479], [480]<br>• Parasites (e.g. *Toxoplasma gondii* or *Cryptosporidium parvum / Cryptosporidium hominis*) [481], [482].
**Antibody deficiency**	• Mainly encapsulated bacteria, e.g. *Streptococcus pneumoniae* or *Haemophilus influenzae*<br>• Viruses (CMV, HSV, norovirus, rotavirus, adenovirus)<br>• Fungi (*Candida* spp.)
**Splenectomy/ functional asplenia**	• Capsule-forming bacteria (e.g. pneumococci*, Haemophilus influenzae, Neisseria meningitidis*)<br>• *Salmonella enteritidis*, *Salmonella typhimurium*
**Others**	
**Mucositis of the gastrointestinal tract**	• α-haemolytic (viridans) streptococci (e.g. *Streptococcus mitis*)<br>• *Clostridioides difficile* (and very rarely: *Clostridium septicum)*<br>• *Enterococcus faecium* and *Enterococcus faecalis*<br>• *Leuconostoc* spp.*, Rothia mucilaginosa*<br>• *Campylobacter jejuni*
**Skin lesion/ venous catheter infection**	• Coagulase-negative staphylococci (CoNS, e.g. *Staphylococcus epidermidis*)<br>• *Staphylococcus aureus*<br>• *Corynebacterium* spp.<br>• *Pseudomonas aeruginosa* (Ecthyma gangrenosum), *Stenotrophomonas maltophilia*<br>• Very rarely: Nontuberculous mycobacteria<br>• Fungi (*Aspergillus* spp.)

Table 5: General information on the prevention of food-related infections (and for food preparation by patients in all risk groups and their companions) [5], [58], [59], [60], [163]

Food	High risk	Low risk
Meat including poultry, fish	Raw or insufficiently cooked (e.g. sashimi, insufficiently cooked seafood such as mussels or prawns)<br>• Raw minced pork, tartar and similar foods made of raw minced meat<br>• Raw sliced meat such as carpaccio<br>• Spreadable short-aged raw sausage (e.g. made from raw minced pork and/or beef, Braunschweiger smoked sausage)<br>• Hard salami	Sufficiently cooked[a]<br>(meat that is white or brown in the middle after cooking, clear juices)
Eggs and egg products	Raw or insufficiently cooked	Eggs: hard-boiled egg white and yolk (at least 8 min);<br>if the recipe calls for raw eggs, use pasteurised egg products<br>(e.g. liquid egg)
Dairy products like quark and cheese	Products made from unpasteurised (raw) milk, surface-ripened[b] cheese, whether it is pasteurised or not<br>(e.g. sour milk cheese, Harzer, Mainzer, yellow cheese, Olomouc, Limburger, Münster, Tilsit, etc.)	Products that have at least been pasteurised
Salad	Buffet-style salad bar<br>Raw sprouts	Salad that has been carefully washed and freshly prepared
Water/ice	Tap water that has not been filtered or boiled, still mineral water	Drinking water monitored by infection control specialists (for *Pseudomonas aeruginosa*, etc.); drinking water that has been boiled or filtered with a 0.2 µm filter, tea made with sufficiently boiled water<br>(rapid boil for 1 min)
Fruit and vegetables	Not cleaned or washed	Well washed<br>(perhaps cleaned with a soft brush or peeled)
Nuts		Only vacuum-packed, roasted, shelled nuts; consume within 24 hours
Muesli	Large pack	• Small patient-specific pack consumed within a week<br>• In risk groups 2 and 3, autoclaved individual portions
**A selection of basic hygiene rules for food handling**		
• Pay attention to best-before dates and do not use damaged packs.<br>• Wash hands with soap before preparing food (disinfect hands after touching potentially contaminated meat or fish products).<br>• Use fresh meat and fish within 2 days.<br>• Have separate "unclean" (raw meat, unwashed vegetables, etc.) and clean areas; different areas (and kitchen utensils) for preparing raw meat/poultry/fish, vegetables and other foods.<br>• Store food in a refrigerator with adequate refrigeration (4-8°C).<br>• Store leftover food (including opened fruit juices) in a refrigerator and only for short periods (no more than 24 hours) or freeze and cook thoroughly before eating.<br>• Crockery is best cleaned in a dishwasher (at least 65°C).<br>• Do not use a sponge to wash up, clean kitchen surfaces with disposable wipes, wash kitchen textiles at a minimum of 60°C and iron after drying.		

[a] Core temperature at least 75˚C.

[b] *Brevibacterium* spp., micrococci and yeasts are used to ripen soft cheese and sour milk cheese. During ripening, these cheeses develop a yellowish or reddish-orange surface smear.

Table 6: Selection of infectious agents isolated in zoonotic diseases [61], [62], [63], [310], [311], [312], [313], [314], [315], [316]

Animal species	Zoonotic disease/pathogen
Dog	• *Capnocytophaga canimorsus*, *Pasteurella multocida* (bite wound, wound infection, sepsis; also transmitted though kissing animals, face/wound licking)<br>• Methicillin-resistant *Staphylococcus aureus*<br>• Parasites: intestinal parasites (*Cryptosporidium parvum, Cryptosporidium hominis*), microsporidiosis (*Microsporidium canis*)<br>• Fungi (*Trichophyton mentagrophytes*)
Cat	• Cat scratch disease, peliosis hepatis, bacillary angiomatosis (*Bartonella henselae,* other *Bartonella* spp.), leptospirosis (*Leptospira interrogans, Leptospira icterohaemorrhagiae*), *Pasteurella multocida*<br>• Parasites: toxoplasmosis (*Toxoplasma gondii*)<br>• Fungi: *Malassezia pachydermatis* [483, 484], microsporidiosis (*Microsporidium canis), Trichophyton mentagrophytes,* Sporotrichosis (*Sporothrix schenckii*)
Reptiles (tortoise, snake, gecko)	• Salmonellosis (rare serovars) Caution: chronic shedding!
Hens/poultry	• *Campylobacter* infections<br>• Salmonellosis
Calves, pigs, animal feed	• *Staphylococcus aureus*<br>• *Campylobacter* infections<br>• Yersiniosis, salmonellosis<br>• Enterohaemorrhagic *Escherichia coli* (haemolytic-uraemic syndrome)
Calves, lambs	• Giardiasis (*Giardia lamblia*)
Lambs, flocks of sheep	• Q fever (*Coxiella burnetii*)
Fish tanks	• Atypical mycobacteria (*Mycobacterium marinum*)<br>• *Pseudomonas aeruginosa* and other opportunistic pathogens associated with water
Wild mice	• *Streptobacillus moniliformis*<br>• Hantaviruses
Rats, dogs, mice, hedgehogs	• Leptospirosis (*Leptospira icterohaemorrhagiae*)
Doves	• Cryptococcosis *(Cryptococcus neoformans)*
Budgerigars, parrots	• Psittacosis, parrot fever (*Chlamydia psittaci)*
Horses	• *Rhodococcus equi* (atypical pneumonia)<br>• Aspergillus and other moulds
Mice, guinea pigs, hamsters	• Lymphocytic choriomeningitis (LCM virus)

Compilation 1:

A selection of measures for preventing zoonotic diseases when handling pets [61], [63], [316], [485], [486]

- Hand hygiene (wash with water and soap solution or disinfect the hands) after touching the animal or objects contaminated by the animal (caution: dry food can also be heavily contaminated with pathogens).
- Do not leave small children alone with the pet (supervise contact).
- Do not feed the animal raw meat where possible.
- Do not feed the animal in the kitchen.
- Do not let the pet in the bedroom (or sleep in the same bed).
- Do not kiss the animal and do not get licked (especially wounds). Clean and disinfect scratches and cover with a sterile plaster.
- Do not let immunosuppressed patients clean cat litter trays – clean daily – (bird cages or fish tanks) or wear protective clothing (gloves, FFP2 mask).
- Wash dog blankets and similar textiles at a minimum of 60°C.
- Keep cats in the house where possible.
- Do not let dogs off the lead outside the home.
- Do not acquire any new animals (particularly animals under 6 months old) during immunosuppressive therapy.
- Have the pet checked by a vet and take it to the vet at the first signs of an infection. Inform the vet that a member of the household is immunosuppressed.
- Do not linger in a stable (groom the horse, etc.) for as long as there is an increased risk of invasive fungal infections.

Compilation 2

Possible (a selection of) starting points for antibiotic/antifungal stewardship programmes in immunosuppressed patients

Interdisciplinary development, implementation and evaluation (plan-do-check-act cycles) of internal guidelines on the following subjects

- medication to prevent infection (bacteria, fungi), including in patients with asplenia,
- diagnostic testing and treatment of fever without a focus during neutropenia,
- diagnostic testing and treatment of sepsis during neutropenia or immunosuppressive therapy,
- prevention (in general: prevention bundles for NI [see relevant KRINKO recommendations]), diagnostic testing and treatment of intravascular catheter-related infections,
- empirical (or pre-emptive) treatment with antifungals,
- diagnostic testing, treatment and prevention of CDI,
- diagnostic testing and treatment of bloodstream infections caused by *S. aureus* (including MRSA),
- diagnostic testing and treatment of bloodstream infections caused by *Candida* spp.,
- perioperative antibiotic prophylaxis in patients undergoing chemotherapy and surgical interventions,
- restriction of the use of carbapenems and glycopeptides.

Establishing an ongoing surveillance and reporting system for

- selected nosocomial infections,
- pathogen and resistance statistics (invasive isolates),
- pathogen and resistance statistics (from screening materials),
- the use of anti-infectives, and specifying how the results of this surveillance will be handled by the interdisciplinary team and used to identify any action that needs to be taken.

Preparing an in-house list of anti-infectives with specific information on

- dosage and administration (example: prolonged or continuous administration of certain beta-lactam antibiotics) and the most important adverse reactions and interactions,
- particular anti-infectives which usage should be restricted (where appropriate, only after approval or with a compulsory follow-up review in the form of an infectious diseases consultation),
- drug monitoring, where use of a particular anti-infective requires this.

Strategies for adapting antibiotic treatment
(selecting the most suitable anti-infectives, methods of administration, dosage details, duration of treatment)

- to the patient's risk profile
  (e.g. oral outpatient vs intravenous inpatient treatment),
- to a pre-existing colonisation (or known previous infections) with certain multidrug-resistant pathogens (MRSA, MRGN, VRE),
- because of possible drug interactions (co-medication) or organ dysfunction (e.g. kidney failure, inner ear hearing loss, neuropathy),
- to confirmed pathogens and known antibiotic susceptibility (de-escalation).

References

1. Berner R, Sauter S, Duffner U, Brandis M, Niemeyer CM. Bakteriämie-Episoden bei pädiatrisch-onkologischen Patienten, insbesondere durch Streptokokken der Viridans-Gruppe. Klin Padiatr. 1998;210(4):256-60.
2. Kommission für Krankenhaushygiene und Infektionsprävention (KRINKO). Infektionsprävention im Rahmen der Pflege und Behandlung von Patienten mit übertragbaren Krankheiten. Bundesgesundheitsbl. 2015;58(10):1151-70.
3. Miller HK, Braun TM, Stillwell T, Harris AC, Choi S, Connelly J, Couriel D, Goldstein S, Kitko CL, Magenau J, Pawarode A, Reddy P, Riwes M, Yanik GA, Levine JE. Infectious Risk after Allogeneic Hematopoietic Cell Transplantation Complicated by Acute Graft-versus-Host Disease. Biol Blood Marrow Transplant. 2017 Mar;23(3):522-8. DOI: 10.1016/j.bbmt.2016.12.630
4. Harris AC, Young R, Devine S, Hogan WJ, Ayuk F, Bunworasate U, Chanswangphuwana C, Efebera YA, Holler E, Litzow M, Ordemann R, Qayed M, Renteria AS, Reshef R, Wölfl M, Chen YB, Goldstein S, Jagasia M, Locatelli F, Mielke S, Porter D, Schechter T, Shekhovtsova Z, Ferrara JL, Levine JE. International, Multicenter Standardization of Acute Graft-versus-Host Disease Clinical Data Collection: A Report from the Mount Sinai Acute GVHD International Consortium. Biol Blood Marrow Transplant. 2016;22(1):4-10. DOI: 10.1016/j.bbmt.2015.09.001
5. Fox N, Freifeld AG. The neutropenic diet reviewed: moving toward a safe food handling approach. Oncology (Williston Park). 2012;26(6):572-5, 80, 82 passim.
6. Maia JE, da Cruz LB, Gregianin LJ. Microbiological profile and nutritional quality of a regular diet compared to a neutropenic diet in a pediatric oncology unit. Pediatr Blood Cancer. 2018;65(3):e26828. DOI: 10.1002/pbc.26828
7. Moody KM, Baker RA, Santizo RO, Olmez I, Spies JM, Buthmann A, Granowetter L, Dulman RY, Ayyanar K, Gill JB, Carroll AE. A randomized trial of the effectiveness of the neutropenic diet versus food safety guidelines on infection rate in pediatric oncology patients. Pediatr Blood Cancer. 2018 Jan;65(1). DOI: 10.1002/pbc.26711
8. Singer M, Deutschman CS, Seymour CW, Shankar-Hari M, Annane D, Bauer M, Bellomo R, Bernard GR, Chiche JD, Coopersmith CM, Hotchkiss RS, Levy MM, Marshall JC, Martin GS, Opal SM, Rubenfeld GD, van der Poll T, Vincent JL, Angus DC. The Third International Consensus Definitions for Sepsis and Septic Shock (Sepsis-3). JAMA. 2016 Feb;315(8):801-10. DOI: 10.1001/jama.2016.0287
9. Kochanek M, Schalk E, von Bergwelt-Baildon M, Beutel G, Buchheidt D, Hentrich M, Henze L, Kiehl M, Liebregts T, von Lilienfeld-Toal M, Classen A, Mellinghoff S, Penack O, Piepel C, Böll B. Management of sepsis in neutropenic cancer patients: 2018 guidelines from the Infectious Diseases Working Party (AGIHO) and Intensive Care Working Party (iCHOP) of the German Society of Hematology and Medical Oncology (DGHO). Ann Hematol. 2019 May;98(5):1051-69. DOI: 10.1007/s00277-019-03622-0
10. Seymour CW, Liu VX, Iwashyna TJ, Brunkhorst FM, Rea TD, Scherag A, Rubenfeld G, Kahn JM, Shankar-Hari M, Singer M, Deutschman CS, Escobar GJ, Angus DC. Assessment of Clinical Criteria for Sepsis: For the Third International Consensus Definitions for Sepsis and Septic Shock (Sepsis-3). JAMA. 2016 Feb 23;315(8):762-74. DOI: 10.1001/jama.2016.0288
11. Tsioutis C, Birgand G, Bathoorn E, Deptula A, Ten Horn L, Castro-Sánchez E, Săndulescu O, Widmer AF, Tsakris A, Pieve G, Tacconelli E, Mutters NT. Education and training programmes for infection prevention and control professionals: mapping the current opportunities and local needs in European countries. Antimicrob Resist Infect Control. 2020 11;9(1):183. DOI: 10.1186/s13756-020-00835-1
12. Stosor V, Zembower TR, editors. Infectious Complications in Cancer Patients. Vol 161. Cham (CH): Springer International Publishing; 2014. (Cancer Treatment and Research).
13. Girmenia C, Candoni A, Delia M, Latagliata R, Molteni A, Oliva EN, Palumbo GA, Poloni A, Salutari P, Santini V, Voso MT, Musto P. Infection control in patients with myelodysplastic syndromes who are candidates for active treatment: Expert panel consensus-based recommendations. Blood Rev. 2019 Mar;34:16-25. DOI: 10.1016/j.blre.2018.10.002
14. Kommission für Krankenhaushygiene und Infektionsprävention (KRINKO). Anforderungen an die Hygiene bei der medizinischen Versorgung von immunsupprimierten Patienten. Bundesgesundheitsbl. 2010;53(4):357-88. DOI: 10.1007/s00103-010-1028-9
15. Ständige Impfkommision (STIKO). Hinweise der STIKO zu Impfungen für Patienten mit Immundefizienz. Stand: November 2005. Epid Bull. 2005;39:1-12.
16. Kommission für Krankenhaushygiene und Infektionsprävention (KRINKO). Personelle und organisatorische Voraussetzungen zur Prävention nosokomialer Infektionen. Bundesgesundheitsbl. 2009;53(9):951-62. DOI: 10.1007/s00103-009-0929-y
17. Rolston KV. Infections in Cancer Patients with Solid Tumors: A Review. Infect Dis Ther. 2017 Mar;6(1):69-83. DOI: 10.1007/s40121-017-0146-1
18. Avritscher EB, Cooksley CD, Rolston KV, Swint JM, Delclos GL, Franzini L, Swisher SG, Walsh GL, Mansfield PF, Elting LS. Serious postoperative infections following resection of common solid tumors: outcomes, costs, and impact of hospital surgical volume. Support Care Cancer. 2014 Feb;22(2):527-35. DOI: 10.1007/s00520-013-2006-1
19. Sammon J, Trinh VQ, Ravi P, Sukumar S, Gervais MK, Shariat SF, Larouche A, Tian Z, Kim SP, Kowalczyk KJ, Hu JC, Menon M, Karakiewicz PI, Trinh QD, Sun M. Health care-associated infections after major cancer surgery: temporal trends, patterns of care, and effect on mortality. Cancer. 2013 Jun;119(12):2317-24. DOI: 10.1002/cncr.28027
20. Sammon JD, Klett DE, Sood A, Olugbade K Jr, Schmid M, Kim SP, Menon M, Trinh QD. Sepsis after major cancer surgery. J Surg Res. 2015 Feb;193(2):788-94. DOI: 10.1016/j.jss.2014.07.046

21. Rolston KVI, Nesher L, Tarrand JT. Current Microbiology of Surgical Site Infections in Patients with Cancer: A Retrospective Review. Infect Dis Ther. 2014;3(2):245-56. DOI: 10.1007/s40121-014-0048-4
22. Schreiber PW, Sax H, Wolfensberger A, Clack L, Kuster SP; Swissnoso. The preventable proportion of healthcare-associated infections 2005-2016: Systematic review and meta-analysis. Infect Control Hosp Epidemiol. 2018 11;39(11):1277-95. DOI: 10.1017/ice.2018.183
23. Crossnohere NL, Richardson DR, Reinhart C, O'Donoghue B, Love SM, Smith BD, Bridges JFP. Side effects from acute myeloid leukemia treatment: results from a national survey. Curr Med Res Opin. 2019 Nov;35(11):1965-70. DOI: 10.1080/03007995.2019.1631149
24. Lyman GH, Michels SL, Reynolds MW, Barron R, Tomic KS, Yu J. Risk of mortality in patients with cancer who experience febrile neutropenia. Cancer. 2010 Dec;116(23):5555-63. DOI: 10.1002/cncr.25332
25. Rhee C, Jones TM, Hamad Y, Pande A, Varon J, O'Brien C, Anderson DJ, Warren DK, Dantes RB, Epstein L, Klompas M; Centers for Disease Control and Prevention (CDC) Prevention Epicenters Program. Prevalence, Underlying Causes, and Preventability of Sepsis-Associated Mortality in US Acute Care Hospitals. JAMA Netw Open. 2019 Feb;2(2):e187571. DOI: 10.1001/jamanetworkopen.2018.7571
26. Pergam SA. Infection Prevention in Transplantation. Curr Infect Dis Rep. 2016 Jan;18(2):7. DOI: 10.1007/s11908-015-0513-6
27. Tomblyn M, Chiller T, Einsele H, Gress R, Sepkowitz K, Storek J, Wingard JR, Young JA, Boeckh MJ, Boeckh MA; Center for International Blood and Marrow Research; National Marrow Donor program; European Blood and MarrowTransplant Group; American Society of Blood and Marrow Transplantation; Canadian Blood and Marrow Transplant Group; Infectious Diseases Society of America; Society for Healthcare Epidemiology of America; Association of Medical Microbiology and Infectious Disease Canada; Centers for Disease Control and Prevention. Guidelines for preventing infectious complications among hematopoietic cell transplantation recipients: a global perspective. Biol Blood Marrow Transplant. 2009 Oct;15(10):1143-238. DOI: 10.1016/j.bbmt.2009.06.019
28. Ullmann AJ, Schmidt-Hieber M, Bertz H, Heinz WJ, Kiehl M, Krüger W, Mousset S, Neuburger S, Neumann S, Penack O, Silling G, Vehreschild JJ, Einsele H, Maschmeyer G; Infectious Diseases Working Party of the German Society for Hematology and Medical Oncology (AGIHO/DGHO) and the DAG-KBT (German Working Group for Blood and Marrow Transplantation). Infectious diseases in allogeneic haematopoietic stem cell transplantation: prevention and prophylaxis strategy guidelines 2016. Ann Hematol. 2016 Sep;95(9):1435-55. DOI: 10.1007/s00277-016-2711-1
29. Balletto E, Mikulska M. Bacterial Infections in Hematopoietic Stem Cell Transplant Recipients. Mediterr J Hematol Infect Dis. 2015;7(1):e2015045. DOI: 10.4084/MJHID.2015.045
30. Ariza-Heredia EJ, Chemaly RF. Update on infection control practices in cancer hospitals. CA Cancer J Clin. 2018 Sep;68(5):340-55. DOI: 10.3322/caac.21462
31. Maschmeyer G, Rolston KVI, editors. Infections in Hematology A clinically oriented, compact, and up-to-date overview on all aspects of infections in hematology patients. Heidelberg: Springer; 2015.
32. Bennett J, Dolin R, Blaser M. Mandell, Douglas, and Bennett's Principles and Practice of Infectious Diseases. 9th rev. ed. Philadelphia: Elsevier; 2019
33. Dunbar A, Tai E, Nielsen DB, Shropshire S, Richardson LC. Preventing infections during cancer treatment: development of an interactive patient education website. Clin J Oncol Nurs. 2014 Aug;18(4):426-31. DOI: 10.1188/14.CJON.426-431
34. Kommission für Krankenhaushygiene und Infektionsprävention (KRINKO). Die Kategorien in der Richtlinie für Krankenhaushygiene und Infektionsprävention - Aktualisierung der Definitionen. Mitteilung der Kommission für Krankenhaushygiene und Infektionsprävention. Bundesgesundheitsbl. 2010;53(7):754-6.
35. MTD-Verlag GmbH. Medizinprodukte-Betreiberverordnung (MPBetreibV) – Verordnung über das Errichten, Betreiben und Anwenden von Medizinprodukten (Medizinprodukte-Betreiberverordnung – MPBe-treibV) in der Neufassung vom 21. August 2002 (BGBl. I S. 3397) zuletzt geändert durch Artikel 1 und 2 der Verordnung vom 27. September 2016 (BGBl. I S. 2203). Gültig seit 1. Januar 2017. 2016 [cited 2020 Nov 01]. Available from: https://www.mtd.de/gesundheitssystem/gesetze-verordnungen/medizinprodukte-betreiberverordnung-mpbetreibv.
36. Gudnadottir U, Fritz J, Zerbel S, Bernardo A, Sethi AK, Safdar N. Reducing health care-associated infections: patients want to be engaged and learn about infection prevention. Am J Infect Control. 2013 Nov;41(11):955-8. DOI: 10.1016/j.ajic.2013.03.310
37. Görig T, Dittmann K, Kramer A, Heidecke CD, Diedrich S, Hübner NO. Active involvement of patients and relatives improves subjective adherence to hygienic measures, especially selfreported hand hygiene: Results of the AHOI pilot study. Antimicrob Resist Infect Control. 2019;8:201. DOI: 10.1186/s13756-019-0648-6
38. Butenko S, Lockwood C, McArthur A. Patient experiences of partnering with healthcare professionals for hand hygiene compliance: a systematic review. JBI Database System Rev Implement Rep. 2017 Jun;15(6):1645-70. DOI: 10.11124/JBISRIR-2016-003001
39. Fernandes Agreli H, Murphy M, Creedon S, Ni Bhuachalla C, O'Brien D, Gould D, Savage E, Barry F, Drennan J, Smiddy MP, Condell S, Horgan S, Murphy S, Wills T, Burton A, Hegarty J. Patient involvement in the implementation of infection prevention and control guidelines and associated interventions: a scoping review. BMJ Open. 2019 Mar 23;9(3):e025824. DOI: 10.1136/bmjopen-2018-025824
40. Birnbach DJ, Nevo I, Barnes S, Fitzpatrick M, Rosen LF, Everett-Thomas R, Sanko JS, Arheart KL. Do hospital visitors wash their hands? Assessing the use of alcohol-based hand sanitizer in a hospital lobby. Am J Infect Control. 2012 May;40(4):340-3. DOI: 10.1016/j.ajic.2011.05.006

41. Wong MWH, Xu YZ, Bone J, Srigley JA. Impact of patient and visitor hand hygiene interventions at a pediatric hospital: A stepped wedge cluster randomized controlled trial. Am J Infect Control. 2020;48(5):511-6. DOI: 10.1016/j.ajic.2019.09.026
42. Srigley JA, Furness CD, Gardam M. Measurement of patient hand hygiene in multiorgan transplant units using a novel technology: an observational study. Infect Control Hosp Epidemiol. 2014 Nov;35(11):1336-41. DOI: 10.1086/678419
43. Gaube S, Fischer P, Windl V, Lermer E. The effect of persuasive messages on hospital visitors' hand hygiene behavior. Health Psychol. 2020 Jun;39(6):471-81. DOI: 10.1037/hea0000854
44. Davis R, Parand A, Pinto A, Buetow S. Systematic review of the effectiveness of strategies to encourage patients to remind healthcare professionals about their hand hygiene. J Hosp Infect. 2015 Mar;89(3):141-62. DOI: 10.1016/j.jhin.2014.11.010
45. von Lengerke T, Kröning B, Lange K; Lower Saxon Diabetes Outpatient Centres Study Group. Patients' intention to speak up for health care providers' hand hygiene in inpatient diabetic foot wound treatment: a cross-sectional survey in diabetes outpatient centres in Lower Saxony, Germany. Psychol Health Med. 2017 Dec;22(10):1137-48. DOI: 10.1080/13548506.2016.1268696
46. Han A, Choi JS. Factors influencing infection prevention self-care behaviors in patients with hematologic cancer after discharge. Eur J Oncol Nurs. 2018 Aug;35:102-6. DOI: 10.1016/j.ejon.2018.06.005
47. Leonard K. A European survey relating to cancer therapy and neutropenic infections: nurse and patient viewpoints. Eur J Oncol Nurs. 2012;16(4):380-6.
48. Yokoe D, Casper C, Dubberke E, Lee G, Muñoz P, Palmore T, Sepkowitz K, Young JA, Donnelly JP; Center for International Blood and Marrow Transplant Research; National Marrow Donor Program; European Blood and Marrow Transplant Group; American Society of Blood and Marrow Transplantation; Canadian Blood and Marrow Transplant Group; Infectious Disease Society of America; Society for Healthcare Epidemiology of America; Association of Medical Microbiology and Infectious Diseases Canada; Centers for Disease Control and Prevention. Safe living after hematopoietic cell transplantation. Bone Marrow Transplant. 2009 Oct;44(8):509-19. DOI: 10.1038/bmt.2009.262
49. Lequilliec N, Raymond R, Vanjak D, Baghdadi N, Boulestreau H, Zahar JR, Gangneux JP. Practices of infectious control management during neutropenia: A survey from 149 French hospitals. J Mycol Med. 2017 Jun;27(2):227-31. DOI: 10.1016/j.mycmed.2017.02.006
50. Thom KA, Kleinberg M, Roghmann MC. Infection prevention in the cancer center. Clin Infect Dis. 2013 Aug;57(4):579-85. DOI: 10.1093/cid/cit290
51. Okada J, Yamamizu Y, Fukai K. Effectiveness of hand hygiene depends on the patient's health condition and care environment. Jpn J Nurs Sci. 2016 Oct;13(4):413-23. DOI: 10.1111/jjns.12122
52. Mody L, Washer LL, Kaye KS, Gibson K, Saint S, Reyes K, Cassone M, Mantey J, Cao J, Altamimi S, Perri M, Sax H, Chopra V, Zervos M. Multidrug-resistant Organisms in Hospitals: What Is on Patient Hands and in Their Rooms? Clin Infect Dis. 2019 Nov;69(11):1837-44. DOI: 10.1093/cid/ciz092
53. Pittet D, Allegranzi B, Sax H, Dharan S, Pessoa-Silva CL, Donaldson L, Boyce JM; WHO Global Patient Safety Challenge, World Alliance for Patient Safety. Evidence-based model for hand transmission during patient care and the role of improved practices. Lancet Infect Dis. 2006 Oct;6(10):641-52. DOI: 10.1016/S1473-3099(06)70600-4
54. Kommission für Krankenhaushygiene und Infektionsprävention (KRINKO). Erratum zu: Händehygiene in Einrichtungen des Gesundheitswesens. Bundesgesundheitsbl. 2016;59(11):1503-4.
55. Kommission für Krankenhaushygiene und Infektionsprävention (KRINKO). Händehygiene in Einrichtungen des Gesundheitswesens. Bundesgesundheitsbl. 2016;59(9):1189-220.
56. Reichardt C, Königer D, Bunte-Schönberger K, van der Linden P, Mönch N, Schwab F, Behnke M, Gastmeier P. Three years of national hand hygiene campaign in Germany: what are the key conclusions for clinical practice? J Hosp Infect. 2013 Feb;83 Suppl 1:S11-6. DOI: 10.1016/S0195-6701(13)60004-3
57. Kampf G, Simon A. Händehygiene bei immunsupprimierten Patienten. In: Kampf G, editor. Kompendium Händehygiene. Wiesbaden: mhp-Verlag; 2017. p. 266-71.
58. Lund BM, O'Brien SJ. The occurrence and prevention of foodborne disease in vulnerable people. Foodborne Pathog Dis. 2011 Sep;8(9):961-73. DOI: 10.1089/fpd.2011.0860
59. Evans EW, Redmond EC. An assessment of food safety information provision for UK chemotherapy patients to reduce the risk of foodborne infection. Public Health. 2017 Dec;153:25-35. DOI: 10.1016/j.puhe.2017.06.017
60. Evans EW, Redmond EC. Food Safety Knowledge and Self-Reported Food-Handling Practices in Cancer Treatment. Oncol Nurs Forum. 2018 Sep;45(5):E98-E110. DOI: 10.1188/18.ONF.E98-E110
61. Stull JW, Stevenson KB. Zoonotic disease risks for immunocompromised and other high-risk clients and staff: promoting safe pet ownership and contact. Vet Clin North Am Small Anim Pract. 2015 Mar;45(2):377-92, vii. DOI: 10.1016/j.cvsm.2014.11.007
62. Gurry GA, Campion V, Premawardena C, Woolley I, Shortt J, Bowden DK, Kaplan Z, Dendle C. High rates of potentially infectious exposures between immunocompromised patients and their companion animals: an unmet need for education. Intern Med J. 2017 Mar;47(3):333-5. DOI: 10.1111/imj.13361
63. Hemsworth S, Pizer B. Pet ownership in immunocompromised children – a review of the literature and survey of existing guidelines. Eur J Oncol Nurs. 2006 Apr;10(2):117-27. DOI: 10.1016/j.ejon.2005.08.001
64. Laws HJ, Baumann U, Bogdan C, Burchard G, Christopeit M, Hecht J, Heininger U, Hilgendorf I, Kern W, Kling K, Kobbe G, Külper W, Lehrnbecher T, Meisel R, Simon A, Ullmann A, de Wit M, Zepp F. Impfen bei Immundefizienz: Anwendungshinweise zu den von der Ständigen Impfkommission empfohlenen Impfungen. (III) Impfen bei hämatologischen und onkologischen Erkrankungen (antineoplastische Therapie,

Stammzelltransplantation), Organtransplantation und Asplenie. Bundesgesundheitsblatt Gesundheitsforschung Gesundheitsschutz. 2020 May;63(5):588-644. DOI: 10.1007/s00103-020-03123-w
65. Kommission für Krankenhaushygiene und Infektionsprävention (KRINKO). Prävention von Infektionen, die von Gefäßkathetern ausgehen. Hinweise zur Implementierung. Informativer Anhang 2 zur Empfehlung der Kommission für Krankenhaushygiene und Infektionsprävention (KRINKO) beim Robert Koch-Institut. Bundesgesundheitsbl. 2017;60(2):231-44.
66. Kommission für Krankenhaushygiene und Infektionsprävention (KRINKO). Prävention von Infektionen, die von Gefäßkathetern ausgehen. Teil 1 – Nichtgetunnelte zentralvenöse Katheter Empfehlung der Kommission für Krankenhaushygiene und Infektionsprävention (KRINKO) beim Robert Koch-Institut. Bundesgesundheitsbl. 2017;60(2):171-206.
67. Kommission für Krankenhaushygiene und Infektionsprävention (KRINKO). Prävention von Infektionen, die von Gefäßkathetern ausgehen. Teil 2 – Periphervenöse Verweilkanülen und arterielle Katheter Empfehlung der Kommission für Krankenhaushygiene und Infektionsprävention (KRINKO) beim Robert Koch-Institut. Bundesgesundheitsbl. 2017;60(2):207-15.
68. Hentrich M, Schalk E, Schmidt-Hieber M, Chaberny I, Mousset S, Buchheidt D, Ruhnke M, Penack O, Salwender H, Wolf HH, Christopeit M, Neumann S, Maschmeyer G, Karthaus M; Infectious Diseases Working Party of the German Society of Hematology and Medical Oncology. Central venous catheter-related infections in hematology and oncology: 2012 updated guidelines on diagnosis, management and prevention by the Infectious Diseases Working Party of the German Society of Hematology and Medical Oncology. Ann Oncol. 2014 May;25(5):936-47. DOI: 10.1093/annonc/mdt545
69. Simon A, Furtwängler R, Laws HJ, Greiner J, Lehrnbecher T, Ammann RA, Schilling F, Graf N. Evidenzbasierte Empfehlungen zur Anwendung dauerhaft implantierter, zentralvenöser Zugänge in der diatrischen Onkologie im Auftrag der Gesellschaft für pädiatrische Onkologie und Hämatologie. 5. vollst. überarb. Aufl. Wiesbaden: mhp Verlag; 2018.
70. DeLa Cruz RF, Caillouet B, Guerrero SS. Strategic patient education program to prevent catheter-related bloodstream infection. Clin J Oncol Nurs. 2012 Feb;16(1):E12-7. DOI: 10.1188/12.CJON.E12-E17
71. Møller T, Adamsen L. Hematologic patients' clinical and psychosocial experiences with implanted long-term central venous catheter: self-management versus professionally controlled care. Cancer Nurs. 2010 Nov-Dec;33(6):426-35. DOI: 10.1097/NCC.0b013e3181dc1908
72. Møller T, Borregaard N, Tvede M, Adamsen L. Patient education – a strategy for prevention of infections caused by permanent central venous catheters in patients with haematological malignancies: a randomized clinical trial. J Hosp Infect. 2005 Dec;61(4):330-41. DOI: 10.1016/j.jhin.2005.01.031
73. Centers for Disease Control and Prevention (CDC), CDC Foundation. 3 Steps Toward Preventing Infections During Cancer Treatment. 2019 [cited 2020 Nov 01]. Available from: https://www.preventcancerinfections.org/
74. Verbund für Angewandte Hygiene e.V. (VAH). Hygiene-Tipps für das Krankenhaus. Informationen zur Infektionsprävention. 2019 [cited 2020 Nov 01]. Available from: https://hygiene-tipps-fuer-kids.de/krankenhaus-projektbeschreibung
75. Exner M, Simon A, Stiftung Deutsche Leukämie- & Lymphom-Hilfe, editors. Infektionen? Nein, danke! Wir tun was dagegen! Vermeidung übertragbarer Krankheiten bei Patienten mit Abwehrschwäche im häuslichen Umfeld. 2017 [cited 2020 Nov 01]. Available from: https://www.leukaemie-hilfe.de/nc/download-informationen.html?tx_drblob_pi1%5BdownloadUid%5D=631
76. Hall CB. Nosocomial respiratory syncytial virus infections: the "Cold War" has not ended. Clin Infect Dis. 2000;31(2):590-6. DOI: 10.1086/313960
77. Libbrecht C, Goutagny MP, Bacchetta J, Ploton C, Bienvenu AL, Bleyzac N, Mialou V, Bertrand Y, Domenech C. Impact of a change in protected environment on the occurrence of severe bacterial and fungal infections in children undergoing hematopoietic stem cell transplantation. Eur J Haematol. 2016 Jul;97(1):70-7. DOI: 10.1111/ejh.12685
78. Picheansanthian W, Chotibang J. Glove utilization in the prevention of cross transmission: a systematic review. JBI Database System Rev Implement Rep. 2015 May;13(4):188-230. DOI: 10.11124/jbisrir-2015-1817
79. Ständige Impfkommission (STIKO) am Robert Koch Institut. Hinweise zu Impfungen bei Patienten mit Immundefizienz. Epid Bull. 2005;39:353-64.
80. Niehues T, Bogdan C, Hecht J, Mertens T, Wiese-Posselt M, Zepp F. Impfen bei Immundefizienz. Bundesgesundheitsbl. 2017;60(6):674-84.
81. Rieger CT, Liss B, Mellinghoff S, Buchheidt D, Cornely OA, Egerer G, Heinz WJ, Hentrich M, Maschmeyer G, Mayer K, Sandherr M, Silling G, Ullmann A, Vehreschild MJGT, von Lilienfeld-Toal M, Wolf HH, Lehners N; German Society of Hematology and Medical Oncology Infectious Diseases Working Group (AGIHO). Anti-infective vaccination strategies in patients with hematologic malignancies or solid tumors-Guideline of the Infectious Diseases Working Party (AGIHO) of the German Society for Hematology and Medical Oncology (DGHO). Ann Oncol. 2018 Jun;29(6):1354-65. DOI: 10.1093/annonc/mdy117
82. El Ramahi R, Freifeld A. Epidemiology, Diagnosis, Treatment, and Prevention of Influenza Infection in Oncology Patients. J Oncol Pract. 2019 Apr;15(4):177-84. DOI: 10.1200/JOP.18.00567
83. Price SA, Podczervinski S, MacLeod K, Helbert L, Pergam SA. Understanding influenza vaccination rates and reasons for refusal in caregivers and household contacts of cancer patients. Am J Infect Control. 2019 Apr;47(4):468-70. DOI: 10.1016/j.ajic.2018.10.010
84. Gesetz für den Schutz vor Masern und zur Stärkung der Impfprävention (Masernschutzgesetz). Vom 10. Februar 2020. BGBl Teil I. Nr. 6. p.148-57.

85. Kommission für Krankenhaushygiene und Infektionsprävention (KRINKO). Impfungen von Personal in medizinischen Einrichtungen in Deutschland: Empfehlung zur Umsetzung der gesetzlichen Regelung in § 23a Infektionsschutzgesetz. Bundesgesundheitsbl. 2021;64(5):636-42.
86. Berg TT, Wicker S. Impfungen für medizinisches Personal. Krankenhaushygiene up2date. 2018;13(03):331-42.
87. Frenzel E, Chemaly RF, Ariza-Heredia E, Jiang Y, Shah DP, Thomas G, Graviss L, Raad I. Association of increased influenza vaccination in health care workers with a reduction in nosocomial influenza infections in cancer patients. Am J Infect Control. 2016 Sep;44(9):1016-21. DOI: 10.1016/j.ajic.2016.03.024
88. Field RI. Mandatory vaccination of health care workers: whose rights should come first? Pharm Ther. 2009;34(11):615-8.
89. Maltezou HC, Poland GA. Vaccination policies for healthcare workers in Europe. Vaccine. 2014 Aug;32(38):4876-80. DOI: 10.1016/j.vaccine.2013.10.046
90. Maltezou HC, Dedoukou X, Vernardaki A, Katerelos P, Kostea E, Tsiodras S, Mentis A, Saroglou G, Theodoridou M, Georgakopoulou T. Measles in healthcare workers during the ongoing epidemic in Greece, 2017–2018. J Hosp Infect. 2018 Dec;100(4):e261-e263. DOI: 10.1016/j.jhin.2018.06.007
91. Maltezou HC, Poland GA. Immunization of healthcare providers: a critical step toward patient safety. Vaccine. 2014 Aug;32(38):4813. DOI: 10.1016/j.vaccine.2014.05.046
92. Montoya A, Schildhouse R, Goyal A, Mann JD, Snyder A, Chopra V, Mody L. How often are health care personnel hands colonized with multidrug- resistant organisms? A systematic review and meta-analysis. Am J Infect Control. 2019 Jun;47(6):693-703. DOI: 10.1016/j.ajic.2018.10.017
93. Biehl LM, Higgins P, Wille T, Peter K, Hamprecht A, Peter S, Dörfel D, Vogel W, Häfner H, Lemmen S, Panse J, Rohde H, Klupp EM, Schafhausen P, Imirzalioglu C, Falgenhauer L, Salmanton-García J, Stecher M, Vehreschild JJ, Seifert H, Vehreschild MJGT. Impact of single-room contact precautions on hospital-acquisition and transmission of multidrug-resistant Escherichia coli: a prospective multicentre cohort study in haematological and oncological wards. Clin Microbiol Infect. 2019 Aug;25(8):1013-20. DOI: 10.1016/j.cmi.2018.12.029
94. Sodré da Costa LS, Neves VM, Marra AR, Sampaio Camargo TZ, Fátima dos Santos Cardoso M, da Silva Victor E, Vogel C, Tahira Colman FA, Laselva CR, Pavão dos Santos OF, Edmond MB. Measuring hand hygiene compliance in a hematology-oncology unit: a comparative study of methodologies. Am J Infect Control. 2013 Nov;41(11):997-1000. DOI: 10.1016/j.ajic.2013.03.301
95. Graf K, Ott E, Wolny M, Tramp N, Vonberg RP, Haverich A, Chaberny IF. Hand hygiene compliance in transplant and other special patient groups: an observational study. Am J Infect Control. 2013 Jun;41(6):503-8. DOI: 10.1016/j.ajic.2012.09.009
96. Fehling P, Hasenkamp J, Unkel S, Thalmann I, Hornig S, Trümper L, Scheithauer S. Effect of gloved hand disinfection on hand hygiene before infection-prone procedures on a stem cell ward. J Hosp Infect. 2019 Nov;103(3):321-7. DOI: 10.1016/j.jhin.2019.06.004
97. Kommission für Krankenhaushygiene und Infektionsprävention (KRINKO). Erratum zu: Infektionsprävention im Rahmen der Pflege und Behandlung von Patienten mit übertragbaren Krankheiten. Bundesgesundheitsbl. 2016;59(1):124-9.
98. Szymczak JE, Smathers S, Hoegg C, Klieger S, Coffin SE, Sammons JS. Reasons Why Physicians and Advanced Practice Clinicians Work While Sick: A Mixed-Methods Analysis. JAMA Pediatr. 2015 Sep;169(9):815-21. DOI: 10.1001/jamapediatrics.2015.0684
99. Bailey ES, Lobaugh-Jin E, Smith B, Sova C, Misuraca J, Henshaw N, Gray GC. Molecular epidemiology of an outbreak of human parainfluenza virus 3 among oncology patients. J Hosp Infect. 2019 Nov;103(3):349-53. DOI: 10.1016/j.jhin.2019.07.012
100. Chemaly RF, Shah DP, Boeckh MJ. Management of respiratory viral infections in hematopoietic cell transplant recipients and patients with hematologic malignancies. Clin Infect Dis. 2014 Nov;59 Suppl 5:S344-51. DOI: 10.1093/cid/ciu623
101. Campbell AP, Guthrie KA, Englund JA, Farney RM, Minerich EL, Kuypers J, Corey L, Boeckh M. Clinical outcomes associated with respiratory virus detection before allogeneic hematopoietic stem cell transplant. Clin Infect Dis. 2015 Jul;61(2):192-202. DOI: 10.1093/cid/civ272
102. Geis S, Prifert C, Weissbrich B, Lehners N, Egerer G, Eisenbach C, Buchholz U, Aichinger E, Dreger P, Neben K, Burkhardt U, Ho AD, Kräusslich HG, Heeg K, Schnitzler P. Molecular characterization of a respiratory syncytial virus outbreak in a hematology unit in Heidelberg, Germany. J Clin Microbiol. 2013 Jan;51(1):155-62. DOI: 10.1128/JCM.02151-12
103. Lehners N, Tabatabai J, Prifert C, Wedde M, Puthenparambil J, Weissbrich B, Biere B, Schweiger B, Egerer G, Schnitzler P. Long-Term Shedding of Influenza Virus, Parainfluenza Virus, Respiratory Syncytial Virus and Nosocomial Epidemiology in Patients with Hematological Disorders. PLoS One. 2016;11(2):e0148258. DOI: 10.1371/journal.pone.0148258
104. von Lilienfeld-Toal M, Berger A, Christopeit M, Hentrich M, Heussel CP, Kalkreuth J, Klein M, Kochanek M, Penack O, Hauf E, Rieger C, Silling G, Vehreschild M, Weber T, Wolf HH, Lehners N, Schalk E, Mayer K. Community acquired respiratory virus infections in cancer patients-Guideline on diagnosis and management by the Infectious Diseases Working Party of the German Society for haematology and Medical Oncology. Eur J Cancer. 2016 Nov;67:200-12. DOI: 10.1016/j.ejca.2016.08.015
105. Hijano DR, Maron G, Hayden RT. Respiratory Viral Infections in Patients With Cancer or Undergoing Hematopoietic Cell Transplant. Front Microbiol. 2018;9:3097. DOI: 10.3389/fmicb.2018.03097
106. Shah DP, Ghantoji SS, Mulanovich VE, Ariza-Heredia EJ, Chemaly RF. Management of respiratory viral infections in hematopoietic cell transplant recipients. Am J Blood Res. 2012;2(4):203-18.

107. Shah DP, Shah PK, Azzi JM, El Chaer F, Chemaly RF. Human metapneumovirus infections in hematopoietic cell transplant recipients and hematologic malignancy patients: A systematic review. Cancer Lett. 2016 Aug;379(1):100-6. DOI: 10.1016/j.canlet.2016.05.035
108. Sung L, Alonzo TA, Gerbing RB, Aplenc R, Lange BJ, Woods WG, Feusner J, Franklin J, Patterson MJ, Gamis AS; Children's Oncology Group. Respiratory syncytial virus infections in children with acute myeloid leukemia: a report from the Children's Oncology Group. Pediatr Blood Cancer. 2008 Dec;51(6):784-6. DOI: 10.1002/pbc.21710
109. Khawaja F, Chemaly RF. Respiratory syncytial virus in hematopoietic cell transplant recipients and patients with hematologic malignancies. Haematologica. 2019 Jul;104(7):1322-31. DOI: 10.3324/haematol.2018.215152
110. Sokol KA, De la Vega-Diaz I, Edmondson-Martin K, Kim S, Tindle S, Wallach F, Steinberg A. Masks for prevention of respiratory viruses on the BMT unit: results of a quality initiative. Transpl Infect Dis. 2016 Dec;18(6):965-7. DOI: 10.1111/tid.12608
111. Sung AD, Sung JAM, Thomas S, Hyslop T, Gasparetto C, Long G, Rizzieri D, Sullivan KM, Corbet K, Broadwater G, Chao NJ, Horwitz ME. Universal Mask Usage for Reduction of Respiratory Viral Infections After Stem Cell Transplant: A Prospective Trial. Clin Infect Dis. 2016 Oct;63(8):999-1006. DOI: 10.1093/cid/ciw451
112. Chu HY, Englund JA, Podczervinski S, Kuypers J, Campbell AP, Boeckh M, Pergam SA, Casper C. Nosocomial transmission of respiratory syncytial virus in an outpatient cancer center. Biol Blood Marrow Transplant. 2014 Jun;20(6):844-51. DOI: 10.1016/j.bbmt.2014.02.024
113. Huang SS. Chlorhexidine-based decolonization to reduce healthcare-associated infections and multidrug-resistant organisms (MDROs): who, what, where, when, and why? J Hosp Infect. 2019 Nov;103(3):235-43. DOI: 10.1016/j.jhin.2019.08.025
114. Messler S, Klare I, Wappler F, Werner G, Ligges U, Sakka SG, Mattner F. Reduction of nosocomial bloodstream infections and nosocomial vancomycin-resistant Enterococcus faecium on an intensive care unit after introduction of antiseptic octenidine-based bathing. J Hosp Infect. 2019 Mar;101(3):264-71. DOI: 10.1016/j.jhin.2018.10.023
115. Fan CY, Lee WT, Hsu TC, Lee CH, Wang SP, Chen WS, Huang CH, Lee CC. Effect of chlorhexidine bathing on colonization or infection with Acinetobacter baumannii: a systematic review and meta-analysis. J Hosp Infect. 2019 Nov;103(3):284-92. DOI: 10.1016/j.jhin.2019.08.004
116. Huang SS, Septimus E, Kleinman K, Moody J, Hickok J, Heim L, Gombosev A, Avery TR, Haffenreffer K, Shimelman L, Hayden MK, Weinstein RA, Spencer-Smith C, Kaganov RE, Murphy MV, Forehand T, Lankiewicz J, Coady MH, Portillo L, Sarup-Patel J, Jernigan JA, Perlin JB, Platt R; ABATE Infection trial team. Chlorhexidine versus routine bathing to prevent multidrug-resistant organisms and all-cause bloodstream infections in general medical and surgical units (ABATE Infection trial): a cluster-randomised trial. Lancet. 2019 Mar;393(10177):1205-15. DOI: 10.1016/S0140-6736(18)32593-5
117. Snarski E, Mank A, Iacobelli S, Hoek J, Styczyński J, Babic A, Cesaro S, Johansson E. Current practices used for the prevention of central venous catheter-associated infection in hematopoietic stem cell transplantation recipients: a survey from the Infectious Diseases Working Party and Nurses' Group of EBMT. Transpl Infect Dis. 2015 Aug;17(4):558-65. DOI: 10.1111/tid.12399
118. Kommission für Krankenhaushygiene und Infektionsprävention (KRINKO). Empfehlungen zur Prävention und Kontrolle von Methicillin-resistenten Staphylococcus aureus-Stämmen (MRSA) in medizinischen und pflegerischen Einrichtungen. Bundesgesundheitsbl. 2014;57(6):696-732.
119. Kommission für Krankenhaushygiene und Infektionsprävention (KRINKO). Prävention Gefäßkatheter-assoziierter Infektionen - Empfehlung der Kommission für Krankenhaushygiene und Infektionsprävention am Robert Koch-Institut. Bundesgesundheitsbl. 2002;25(11):907-24.
120. Kommission für Krankenhaushygiene und Infektionsprävention (KRINKO). Prävention von Infektionen, die von Gefäßkathetern ausgehen. Hinweise zur Blutkulturdiagnostik. Informativer Anhang 1 zur Empfehlung der Kommission für Krankenhaushygiene und Infektionsprävention (KRINKO) beim Robert Koch-Institut. Bundesgesundheitsbl. 2017;60(2):216-30.
121. Raulji CM, Clay K, Velasco C, Yu LC. Daily Bathing with Chlorhexidine and Its Effects on Nosocomial Infection Rates in Pediatric Oncology Patients. Pediatr Hematol Oncol. 2015;32(5):315-21. DOI: 10.3109/08880018.2015.1013588
122. Choi SW, Chang L, Hanauer DA, Shaffer-Hartman J, Teitelbaum D, Lewis I, Blackwood A, Akcasu N, Steel J, Christensen J, Niedner MF. Rapid reduction of central line infections in hospitalized pediatric oncology patients through simple quality improvement methods. Pediatr Blood Cancer. 2013 Feb;60(2):262-9. DOI: 10.1002/pbc.24187
123. Climo MW, Yokoe DS, Warren DK, Perl TM, Bolon M, Herwaldt LA, Weinstein RA, Sepkowitz KA, Jernigan JA, Sanogo K, Wong ES. Effect of daily chlorhexidine bathing on hospital-acquired infection. N Engl J Med. 2013 Feb;368(6):533-42. DOI: 10.1056/NEJMoa1113849
124. Abbas M, Pires D, Peters A, Morel CM, Hurst S, Holmes A, Saito H, Allegranzi B, Lucet JC, Zingg W, Harbarth S, Pittet D. Conflicts of interest in infection prevention and control research: no smoke without fire. A narrative review. Intensive Care Med. 2018 Oct;44(10):1679-90. DOI: 10.1007/s00134-018-5361-z
125. Heo ST, Kim SJ, Jeong YG, Bae IG, Jin JS, Lee JC. Hospital outbreak of Burkholderia stabilis bacteraemia related to contaminated chlorhexidine in haematological malignancy patients with indwelling catheters. J Hosp Infect. 2008 Nov;70(3):241-5. DOI: 10.1016/j.jhin.2008.07.019
126. Gastmeier P, Kämpf KP, Behnke M, Geffers C, Schwab F. An observational study of the universal use of octenidine to decrease nosocomial bloodstream infections and MDR organisms. J Antimicrob Chemother. 2016 Sep;71(9):2569-76. DOI: 10.1093/jac/dkw170

127. Meißner A, Hasenclever D, Brosteanu O, Chaberny IF. EFFECT of daily antiseptic body wash with octenidine on nosocomial primary bacteraemia and nosocomial multidrug-resistant organisms in intensive care units: design of a multicentre, cluster-randomised, double-blind, cross-over study. BMJ Open. 2017 Nov;7(11):e016251. DOI: 10.1136/bmjopen-2017-016251
128. Becker SL, Berger FK, Feldner SK, Karliova I, Haber M, Mellmann A, Schäfers HJ, Gärtner B. Outbreak of complex infections associated with contaminated octenidine mouthwash solution, Germany, August to September 2018. Euro Surveill. 2018 Oct;23(42). DOI: 10.2807/1560-7917.ES.2018.23.42.1800540
129. Huang SS, Septimus E, Hayden MK, Kleinman K, Sturtevant J, Avery TR, Moody J, Hickok J, Lankiewicz J, Gombosev A, Kaganov RE, Haffenreffer K, Jernigan JA, Perlin JB, Platt R, Weinstein RA; Agency for Healthcare Research and Quality (AHRQ) DEcIDE Network and Healthcare-Associated Infections Program, and the CDC Prevention Epicenters Program. Effect of body surface decolonisation on bacteriuria and candiduria in intensive care units: an analysis of a cluster-randomised trial. Lancet Infect Dis. 2016 Jan;16(1):70-9. DOI: 10.1016/S1473-3099(15)00238-8
130. Wang EW, Layon AJ. Chlorhexidine gluconate use to prevent hospital acquired infections-a useful tool, not a panacea. Ann Transl Med. 2017;5(1):14.
131. Kampf G. Acquired resistance to chlorhexidine – is it time to establish an ‘antiseptic’ stewardship’ initiative? J Hosp Infect. 2016;94(3):213-27.
132. McNeil JC, Hulten KG, Kaplan SL, Mahoney DH, Mason EO. Staphylococcus aureus infections in pediatric oncology patients: high rates of antimicrobial resistance, antiseptic tolerance and complications. Pediatr Infect Dis J. 2013 Feb;32(2):124-8. DOI: 10.1097/INF.0b013e318271c4e0
133. Kommission für Krankenhaushygiene und Infektionsprävention (KRINKO). Anforderung an die Hygiene bei der Reinigung und Desinfektion von Flächen. Bundesgesundheitsbl. 2004;47(1):51-61.
134. Kommission für Krankenhaushygiene und Infektionsprävention (KRINKO). Hygienemaßnahmen zur Prävention der Infektion durch Enterokokken mit speziellen Antibiotikaresistenzen. Bundesgesundheitsbl. 2018;61(10):1310–61.
135. Kommission für Krankenhaushygiene und Infektionsprävention (KRINKO). Hygienemaßnahmen bei Infektionen oder Besiedlung mit multiresistenten gramnegativen Stäbchen. Bundesgesundheitsbl. 2012;55(10):1311-54.
136. Kommission für Krankenhaushygiene und Infektionsprävention (KRINKO). Hygienemaßnahmen bei Clostridioides difficile-Infektion (CDI). Bundesgesundheitsbl. 2019;62(7):906–23.
137. Kanamori H, Rutala WA, Weber DJ. The Role of Patient Care Items as a Fomite in Healthcare-Associated Outbreaks and Infection Prevention. Clin Infect Dis. 2017 Oct;65(8):1412-19. DOI: 10.1093/cid/cix462
138. Donskey CJ. Does improving surface cleaning and disinfection reduce health care-associated infections? Am J Infect Control. 2013 May;41(5 Suppl):S12-9. DOI: 10.1016/j.ajic.2012.12.010
139. Han JH, Sullivan N, Leas BF, Pegues DA, Kaczmarek JL, Umscheid CA. Cleaning Hospital Room Surfaces to Prevent Health Care-Associated Infections: A Technical Brief. Ann Intern Med. 2015 Oct;163(8):598-607. DOI: 10.7326/M15-1192
140. Leas BF, Sullivan N, Han JH, Pegues DA, Kaczmarek JL, Umscheid CA. Environmental Cleaning for the Prevention of Healthcare-Associated Infections. Rockville (MD): Agency for Healthcare Research and Quality (AHRQ); 2015.
141. Havill NL. Best practices in disinfection of noncritical surfaces in the health care setting: creating a bundle for success. Am J Infect Control. 2013 May;41(5 Suppl):S26-30. DOI: 10.1016/j.ajic.2012.10.028
142. Satlin MJ, Chavda KD, Baker TM, Chen L, Shashkina E, Soave R, Small CB, Jacobs SE, Shore TB, van Besien K, Westblade LF, Schuetz AN, Fowler VG Jr, Jenkins SG, Walsh TJ, Kreiswirth BN. Colonization With Levofloxacin-resistant Extended-spectrum β-Lactamase-producing Enterobacteriaceae and Risk of Bacteremia in Hematopoietic Stem Cell Transplant Recipients. Clin Infect Dis. 2018 Nov;67(11):1720-8. DOI: 10.1093/cid/ciy363
143. Satlin MJ, Jenkins SG, Walsh TJ. The global challenge of carbapenem-resistant Enterobacteriaceae in transplant recipients and patients with hematologic malignancies. Clin Infect Dis. 2014 May;58(9):1274-83. DOI: 10.1093/cid/ciu052
144. Satlin MJ, Walsh TJ. Multidrug-resistant Enterobacteriaceae, Pseudomonas aeruginosa, and vancomycin-resistant Enterococcus: Three major threats to hematopoietic stem cell transplant recipients. Transpl Infect Dis. 2017 Dec;19(6). DOI: 10.1111/tid.12762
145. Vehreschild MJ, Hamprecht A, Peterson L, Schubert S, Häntschel M, Peter S, Schafhausen P, Rohde H, Lilienfeld-Toal MV, Bekeredjian-Ding I, Libam J, Hellmich M, Vehreschild JJ, Cornely OA, Seifert H. A multicentre cohort study on colonization and infection with ESBL-producing Enterobacteriaceae in high-risk patients with haematological malignancies. J Antimicrob Chemother. 2014 Dec;69(12):3387-92. DOI: 10.1093/jac/dku305
146. Vehreschild MJ, Liss BJ, Cornely OA. Intestinal colonisation and blood stream infections due to vancomycin-resistant enterococci (VRE) and extended-spectrum beta-lactamase-producing Enterobacteriaceae (ESBLE) in patients with haematological and oncological malignancies. Infection. 2013 Oct;41(5):1049-50. DOI: 10.1007/s15010-013-0436-9
147. Vehreschild MJ, Weitershagen D, Biehl LM, Tacke D, Waldschmidt D, Töx U, Wisplinghoff H, Von Bergwelt-Baildon M, Cornely OA, Vehreschild JJ. Clostridium difficile infection in patients with acute myelogenous leukemia and in patients undergoing allogeneic stem cell transplantation: epidemiology and risk factor analysis. Biol Blood Marrow Transplant. 2014 Jun;20(6):823-8. DOI: 10.1016/j.bbmt.2014.02.022

148. Ruhnke M, Arnold R, Gastmeier P. Infection control issues in patients with haematological malignancies in the era of multidrug-resistant bacteria. Lancet Oncol. 2014 Dec;15(13):e606-e619. DOI: 10.1016/S1470-2045(14)70344-4
149. Pouch SM, Satlin MJ. Carbapenem-resistant Enterobacteriaceae in special populations: Solid organ transplant recipients, stem cell transplant recipients, and patients with hematologic malignancies. Virulence. 2017 May;8(4):391-402. DOI: 10.1080/21505594.2016.1213472
150. Medernach RL, Logan LK. The Growing Threat of Antibiotic Resistance in Children. Infect Dis Clin North Am. 2018 Mar;32(1):1-17. DOI: 10.1016/j.idc.2017.11.001
151. van Loon K, Voor In 't Holt AF, Vos MC. A Systematic Review and Meta-analyses of the Clinical Epidemiology of Carbapenem-Resistant Enterobacteriaceae. Antimicrob Agents Chemother. 2018;62:e01730-17.
152. Rump B, Timen A, Hulscher M, Verweij M. Ethics of Infection Control Measures for Carriers of Antimicrobial Drug-Resistant Organisms. Emerg Infect Dis. 2018 Sep;24(9):1609-16. DOI: 10.3201/eid2409.171644
153. Kommission für Krankenhaushygiene (KRINKO). Ergänzung zur Empfehlung der KRINKO „Hygienemaßnahmen bei Infektionen oder Besiedlung mit multiresistenten gramnegativen Stäbchen" (2012) im Zusammenhang mit der von EUCAST neu definierten Kategorie „I" bei der Antibiotika-Resistenzbestimmung: Konsequenzen für die Definition von MRGN. Epid Bull. 2019;9:82-3.
154. Rohde AM, Zweigner J, Wiese-Posselt M, Schwab F, Behnke M, Kola A, Obermann B, Knobloch JK, Feihl S, Querbach C, Gebhardt F, Mischnik A, Ihle V, Schröder W, Armean S, Peter S, Tacconelli E, Hamprecht A, Seifert H, Vehreschild MJGT, Kern WV, Gastmeier P; DZIF-ATHOS study group. Incidence of infections due to third generation cephalosporin-resistant – a prospective multicentre cohort study in six German university hospitals. Antimicrob Resist Infect Control. 2018;7:159. DOI: 10.1186/s13756-018-0452-8
155. Boldt AC, Schwab F, Rohde AM, Kola A, Bui MT, Märtin N, Kipnis M, Schröder C, Leistner R, Wiese-Posselt M, Zweigner J, Gastmeier P, Denkel LA. Admission prevalence of colonization with third-generation cephalosporin-resistant Enterobacteriaceae and subsequent infection rates in a German university hospital. PLoS One. 2018 Aug 1;13(8):e0201548. DOI: 10.1371/journal.pone.0201548
156. Biehl LM, Schmidt-Hieber M, Liss B, Cornely OA, Vehreschild MJ. Colonization and infection with extended spectrum beta-lactamase producing Enterobacteriaceae in high-risk patients – Review of the literature from a clinical perspective. Crit Rev Microbiol. 2016;42(1):1-16. DOI: 10.3109/1040841X.2013.875515
157. Cattaneo C, Di Blasi R, Skert C, Candoni A, Martino B, Di Renzo N, Delia M, Ballanti S, Marchesi F, Mancini V, Orciuolo E, Cesaro S, Prezioso L, Fanci R, Nadali G, Chierichini A, Facchini L, Picardi M, Malagola M, Orlando V, Trecarichi EM, Tumbarello M, Aversa F, Rossi G, Pagano L; SEIFEM Group. Bloodstream infections in haematological cancer patients colonized by multidrug-resistant bacteria. Ann Hematol. 2018 Sep;97(9):1717-26. DOI: 10.1007/s00277-018-3341-6
158. Deutsche Gesellschaft für Pädiatrische Infektiologie (DGPI), Paed IC. Infektionspräventives Vorgehen bei Nachweis von MRGN im Kindesalter. Hyg Med. 2014;39(10):392-9.
159. Joint FAO/WHO Codex Alimentarius Commission. General Requirements (Food Hygiene). Codex Alimentarius (Supplement to Volume 1B). Rome: Food and Agriculture Organization of the United Nations (FAO), World Health Organization (WHO); 1997.
160. De Waele E, Demol J, Caccialanza R, Cotogni P, Spapen H, Malbrain ML, De Grève J, Pen JJ. Unidentified cachexia patients in the oncologic setting: Cachexia UFOs do exist. Nutrition. 2019 Jul-Aug;63-64:200-4. DOI: 10.1016/j.nut.2019.02.015
161. Isenring EA, Teleni L. Nutritional counseling and nutritional supplements: a cornerstone of multidisciplinary cancer care for cachectic patients. Curr Opin Support Palliat Care. 2013 Dec;7(4):390-5. DOI: 10.1097/SPC.0000000000000016
162. Kurk S, Peeters P, Stellato R, Dorresteijn B, de Jong P, Jourdan M, Creemers GJ, Erdkamp F, de Jongh F, Kint P, Simkens L, Tanis B, Tjin-A-Ton M, Van Der Velden A, Punt C, Koopman M, May A. Skeletal muscle mass loss and dose-limiting toxicities in metastatic colorectal cancer patients. J Cachexia Sarcopenia Muscle. 2019 Aug;10(4):803-83. DOI: 10.1002/jcsm.12436
163. Schmid I, Albert MH, Stachel D, Simon A. Nahrungsmittelrestriktionen zur Infektionsprävention bei Kindern mit Krebserkrankung: Was ist gesichert und was ist sinnvoll? Hyg Med. 2008;33(1/2):16-24.
164. Baumgartner A, Hoskin K, Schuetz P. Optimization of nutrition during allogeneic hematologic stem cell transplantation. Curr Opin Clin Nutr Metab Care. 2018 May;21(3):152-8. DOI: 10.1097/MCO.0000000000000461
165. Friedemann M. Gesundheitliches Gefährdungspotenzial von Enterobacter sakazakii (Cronobacter spp. nov.) in Säuglingsnahrung. Bundesgesungheitsbl. 2008;51(6):664-74.
166. Healy B, Cooney S, O'Brien S, Iversen C, Whyte P, Nally J, Callanan JJ, Fanning S. Cronobacter (Enterobacter sakazakii): an opportunistic foodborne pathogen. Foodborne Pathog Dis. 2010 Apr;7(4):339-50. DOI: 10.1089/fpd.2009.0379
167. Holý O, Forsythe S. Cronobacter spp. as emerging causes of healthcare-associated infection. J Hosp Infect. 2014 Mar;86(3):169-77. DOI: 10.1016/j.jhin.2013.09.011
168. Hurrell E, Kucerova E, Loughlin M, Caubilla-Barron J, Hilton A, Armstrong R, Smith C, Grant J, Shoo S, Forsythe S. Neonatal enteral feeding tubes as loci for colonisation by members of the Enterobacteriaceae. BMC Infect Dis. 2009 Sep;9:146. DOI: 10.1186/1471-2334-9-146
169. Gardner A, Mattiuzzi G, Faderl S, Borthakur G, Garcia-Manero G, Pierce S, Brandt M, Estey E. Randomized comparison of cooked and noncooked diets in patients undergoing remission induction therapy for acute myeloid leukemia. J Clin Oncol. 2008 Dec;26(35):5684-8. DOI: 10.1200/JCO.2008.16.4681

170. Lassiter M, Schneider SM. A pilot study comparing the neutropenic diet to a non-neutropenic diet in the allogeneic hematopoietic stem cell transplantation population. Clin J Oncol Nurs. 2015;19(3):273-8.
171. van Tiel F, Harbers MM, Terporten PH, van Boxtel RT, Kessels AG, Voss GB, Schouten HC. Normal hospital and low-bacterial diet in patients with cytopenia after intensive chemotherapy for hematological malignancy: a study of safety. Ann Oncol. 2007 Jun;18(6):1080-4. DOI: 10.1093/annonc/mdm082
172. Sonbol M, Jain T, Firwana B. Neutropenic diets to prevent cancer infections: updated systematic review and meta-analysis. BMJ supportive & palliative care. 2019;9(4):425-33.
173. Ball S, Brown TJ, Das A, Khera R, Khanna S, Gupta A. Effect of Neutropenic Diet on Infection Rates in Cancer Patients With Neutropenia: A Meta-analysis of Randomized Controlled Trials. Am J Clin Oncol. 2019 Mar;42(3):270-4. DOI: 10.1097/COC.0000000000000514
174. Wolfe HR, Sadeghi N, Agrawal D, Johnson DH, Gupta A. Things We Do For No Reason: Neutropenic Diet. J Hosp Med. 2018;13(8):573-6.
175. Tramsen L, Salzmann-Manrique E, Bochennek K, Klingebiel T, Reinhardt D, Creutzig U, Sung L, Lehrnbecher T. Lack of Effectiveness of Neutropenic Diet and Social Restrictions as Anti-Infective Measures in Children With Acute Myeloid Leukemia: An Analysis of the AML-BFM 2004 Trial. J Clin Oncol. 2016 Aug 10;34(23):2776-83. DOI: 10.1200/JCO.2016.66.7881
176. Taggart C, Neumann N, Alonso PB, Lane A, Pate A, Stegman A, Stendahl A, Davies SM, Dandoy CE, Grimley M. Comparing a Neutropenic Diet to a Food Safety-Based Diet in Pediatric Patients Undergoing Hematopoietic Stem Cell Transplantation. Biol Blood Marrow Transplant. 2019 Jul;25(7):1382-6. DOI: 10.1016/j.bbmt.2019.03.017
177. Trifilio S, Helenowski I, Giel M, Gobel B, Pi J, Greenberg D, Mehta J. Questioning the role of a neutropenic diet following hematopoetic stem cell transplantation. Biol Blood Marrow Transplant. 2012 Sep;18(9):1385-90. DOI: 10.1016/j.bbmt.2012.02.015
178. Moody K, Finlay J, Mancuso C, Charlson M. Feasibility and safety of a pilot randomized trial of infection rate: neutropenic diet versus standard food safety guidelines. J Pediatr Hematol Oncol. 2006 Mar;28(3):126-33. DOI: 10.1097/01.mph.0000210412.33630.fb
179. Goldenberg JZ, Yap C, Lytvyn L, Lo CK, Beardsley J, Mertz D, Johnston BC. Probiotics for the prevention of Clostridium difficile-associated diarrhea in adults and children. Cochrane Database Syst Rev. 2017 Dec;12:CD006095. DOI: 10.1002/14651858.CD006095.pub4
180. Guo Q, Goldenberg JZ, Humphrey C, El Dib R, Johnston BC. Probiotics for the prevention of pediatric antibiotic-associated diarrhea. Cochrane Database Syst Rev. 2019 Apr;4:CD004827. DOI: 10.1002/14651858.CD004827.pub5
181. Ouyang X, Li Q, Shi M, Niu D, Song W, Nian Q, Li X, Ding Z, Ai X, Wang J. Probiotics for preventing postoperative infection in colorectal cancer patients: a systematic review and meta-analysis. Int J Colorectal Dis. 2019 Mar;34(3):459-69. DOI: 10.1007/s00384-018-3214-4
182. Alsadat N, Hyun K, Boroumand F, Juergens C, Kritharides L, Brieger DB. Achieving lipid targets within 12 months of an acute coronary syndrome: an observational analysis. Med J Aust. 2022 Mar 02. DOI: 10.5694/mja2.51442
183. Kujawa-Szewieczek A, Adamczak M, Kwiecień K, Dudzicz S, Gazda M, Więcek A. The Effect of Lactobacillus plantarum 299v on the Incidence of Clostridium difficile Infection in High Risk Patients Treated with Antibiotics. Nutrients. 2015 Dec;7(12):10179-88. DOI: 10.3390/nu7125526
184. Bai J, Behera M, Bruner DW. The gut microbiome, symptoms, and targeted interventions in children with cancer: a systematic review. Support Care Cancer. 2018 Feb;26(2):427-39. DOI: 10.1007/s00520-017-3982-3
185. Gorshein E, Wei C, Ambrosy S, Budney S, Vivas J, Shenkerman A, Manago J, McGrath MK, Tyno A, Lin Y, Patel V, Gharibo M, Schaar D, Jenq RR, Khiabanian H, Strair R. Lactobacillus rhamnosus GG probiotic enteric regimen does not appreciably alter the gut microbiome or provide protection against GVHD after allogeneic hematopoietic stem cell transplantation. Clin Transplant. 2017 May;31(5). DOI: 10.1111/ctr.12947
186. Cohen SA, Woodfield MC, Boyle N, Stednick Z, Boeckh M, Pergam SA. Incidence and outcomes of bloodstream infections among hematopoietic cell transplant recipients from species commonly reported to be in over-the-counter probiotic formulations. Transpl Infect Dis. 2016 Oct;18(5):699-705. DOI: 10.1111/tid.12587
187. Salminen MK, Rautelin H, Tynkkynen S, Poussa T, Saxelin M, Valtonen V, Järvinen A. Lactobacillus bacteremia, species identification, and antimicrobial susceptibility of 85 blood isolates. Clin Infect Dis. 2006 Mar;42(5):e35-44. DOI: 10.1086/500214
188. Land MH, Rouster-Stevens K, Woods CR, Cannon ML, Cnota J, Shetty AK. Lactobacillus sepsis associated with probiotic therapy. Pediatrics. 2005 Jan;115(1):178-81. DOI: 10.1542/peds.2004-2137
189. Kunz AN, Fairchok MP, Noel JM. Lactobacillus sepsis associated with probiotic therapy. Pediatrics. 2005 Aug;116(2):517; author reply 517-8. DOI: 10.1542/peds.2005-0475
190. Cannon JP, Lee TA, Bolanos JT, Danziger LH. Pathogenic relevance of Lactobacillus: a retrospective review of over 200 cases. Eur J Clin Microbiol Infect Dis. 2005 Jan;24(1):31-40. DOI: 10.1007/s10096-004-1253-y
191. Arpi M, Vancanneyt M, Swings J, Leisner JJ. Six cases of Lactobacillus bacteraemia: identification of organisms and antibiotic susceptibility and therapy. Scand J Infect Dis. 2003;35(6-7):404-8. DOI: 10.1080/00365540310011830
192. Carretto E, Barbarini D, Marzani FC, Fumagalli P, Monzillo V, Marone P, Emmi V. Catheter-related bacteremia due to Lactobacillus rhamnosus in a single-lung transplant recipient. Scand J Infect Dis. 2001;33(10):780-2. DOI: 10.1080/003655401317074653
193. Cooper CD, Vincent A, Greene JN, Sandin RL, Cobian L. Lactobacillus bacteremia in febrile neutropenic patients in a cancer hospital. Clin Infect Dis. 1998;26(5):1247-8.

194. Schlegel L, Lemerle S, Geslin P. Lactobacillus species as opportunistic pathogens in immunocompromised patients. Eur J Clin Microbiol Infect Dis. 1998 Dec;17(12):887-8. DOI: 10.1007/s100960050216
195. Muñoz P, Bouza E, Cuenca-Estrella M, Eiros JM, Pérez MJ, Sánchez-Somolinos M, Rincón C, Hortal J, Peláez T. Saccharomyces cerevisiae fungemia: an emerging infectious disease. Clin Infect Dis. 2005 Jun;40(11):1625-34. DOI: 10.1086/429916
196. Herbrecht R, Nivoix Y. Saccharomyces cerevisiae fungemia: an adverse effect of Saccharomyces boulardii probiotic administration. Clin Infect Dis. 2005 Jun;40(11):1635-7. DOI: 10.1086/429926
197. Enache-Angoulvant A, Hennequin C. Invasive Saccharomyces infection: a comprehensive review. Clin Infect Dis. 2005 Dec;41(11):1559-68. DOI: 10.1086/497832
198. Cesaro S, Chinello P, Rossi L, Zanesco L. Saccharomyces cerevisiae fungemia in a neutropenic patient treated with Saccharomyces boulardii. Support Care Cancer. 2000;8(6):504-5.
199. Cassone M, Serra P, Mondello F, Girolamo A, Scafetti S, Pistella E, Venditti M. Outbreak of Saccharomyces cerevisiae subtype boulardii fungemia in patients neighboring those treated with a probiotic preparation of the organism. J Clin Microbiol. 2003 Nov;41(11):5340-3. DOI: 10.1128/JCM.41.11.5340-5343.2003
200. Olver WJ, James SA, Lennard A, Galloway A, Roberts IN, Boswell TC, Russell NH. Nosocomial transmission of Saccharomyces cerevisiae in bone marrow transplant patients. J Hosp Infect. 2002 Dec;52(4):268-72. DOI: 10.1053/jhin.2002.1314
201. Ladas EJ, Bhatia M, Chen L, Sandler E, Petrovic A, Berman DM, Hamblin F, Gates M, Hawks R, Sung L, Nieder M. The safety and feasibility of probiotics in children and adolescents undergoing hematopoietic cell transplantation. Bone Marrow Transplant. 2016 Feb;51(2):262-6. DOI: 10.1038/bmt.2015.275
202. Yelin I, Flett KB, Merakou C, Mehrotra P, Stam J, Snesrud E, Hinkle M, Lesho E, McGann P, McAdam AJ, Sandora TJ, Kishony R, Priebe GP. Genomic and epidemiological evidence of bacterial transmission from probiotic capsule to blood in ICU patients. Nat Med. 2019 Nov;25(11):1728-32. DOI: 10.1038/s41591-019-0626-9
203. Diorio C, Robinson PD, Ammann RA, Castagnola E, Erickson K, Esbenshade A, Fisher BT, Haeusler GM, Kuczynski S, Lehrnbecher T, Phillips R, Cabral S, Dupuis LL, Sung L. Guideline for the Management of Infection in Children and Adolescents With Cancer and Pediatric Hematopoietic Stem-Cell Transplantation Recipients. J Clin Oncol. 2018 Nov;36(31):3162-71. DOI: 10.1200/JCO.18.00407
204. Mehta A, Rangarajan S, Borate U. A cautionary tale for probiotic use in hematopoietic SCT patients-Lactobacillus acidophilus sepsis in a patient with mantle cell lymphoma undergoing hematopoietic SCT. Bone Marrow Transplant. 2013;48(3):461-2.
205. Hota S, Hirji Z, Stockton K, Lemieux C, Dedier H, Wolfaardt G, Gardam MA. Outbreak of multidrug-resistant Pseudomonas aeruginosa colonization and infection secondary to imperfect intensive care unit room design. Infect Control Hosp Epidemiol. 2009 Jan;30(1):25-33. DOI: 10.1086/592700
206. Eckmanns T, Rüden H, Gastmeier P. The influence of high-efficiency particulate air filtration on mortality and fungal infection among highly immunosuppressed patients: a systematic review. J Infect Dis. 2006 May;193(10):1408-18. DOI: 10.1086/503435
207. Passweg JR, Rowlings PA, Atkinson KA, Barrett AJ, Gale RP, Gratwohl A, Jacobsen N, Klein JP, Ljungman P, Russell JA, Schaefer UW, Sobocinski KA, Vossen JM, Zhang MJ, Horowitz MM. Influence of protective isolation on outcome of allogeneic bone marrow transplantation for leukemia. Bone Marrow Transplant. 1998 Jun;21(12):1231-8. DOI: 10.1038/sj.bmt.1701238
208. Menegueti MG, Ferreira LR, Silva MF, Silva AS, Bellissimo-Rodrigues F. Assessment of microbiological air quality in hemato-oncology units and its relationship with the occurrence of invasive fungal infections: an integrative review. Rev Soc Bras Med Trop. 2013 Jul-Aug;46(4):391-6. DOI: 10.1590/0037-8682-0022-2013
209. Cesaro S, Tridello G, Castagnola E, Calore E, Carraro F, Mariotti I, Colombini A, Perruccio K, Decembrino N, Russo G, Maximova N, Baretta V, Caselli D. Retrospective study on the incidence and outcome of proven and probable invasive fungal infections in high-risk pediatric onco-hematological patients. Eur J Haematol. 2017 Sep;99(3):240-8. DOI: 10.1111/ejh.12910
210. Linke C, Tragiannidis A, Ahlmann M, Fröhlich B, Wältermann M, Burkhardt B, Rossig C, Groll AH. Epidemiology and management burden of invasive fungal infections after autologous hematopoietic stem cell transplantation: 10-year experience at a European Pediatric Cancer Center. Mycoses. 2019 Oct;62(10):954-60. DOI: 10.1111/myc.12968
211. Vokurka S, Bystrická E, Svoboda T, Škoda Gorican IK, Sever M, Mazur E, Kopinska A, Pavlicová V, Mocanu O, Tanase A, Ghelase R, Zítková M, Labudíková M, Raida L, Hrabánková-Navrátilová D, Bocková J. The availability of HEPA-filtered rooms and the incidence of pneumonia in patients after haematopoietic stem cell transplantation (HSCT): results from a prospective, multicentre, eastern European study. J Clin Nurs. 2014 Jun;23(11-12):1648-52. DOI: 10.1111/jocn.12286
212. Hicheri Y, Einsele H, Martino R, Cesaro S, Ljungman P, Cordonnier C. Environmental prevention of infection in stem cell transplant recipients: a survey of the Infectious Diseases Working Part of the European Group for Blood and Marrow Transplantation. Transpl Infect Dis. 2013;15:251-8. DOI: 10.1111/tid.12064
213. Ruijters VJ, Oosterom N, Wolfs TFW, van den Heuvel-Eibrink MM, van Grotel M. Frequency and Determinants of Invasive Fungal Infections in Children With Solid and Hematologic Malignancies in a Nonallogeneic Stem Cell Transplantation Setting: A Narrative Review. J Pediatr Hematol Oncol. 2019;41(5):345-54. DOI: 10.1097/MPH.0000000000001468
214. Styczynski J, Tridello G, Donnelly JP, Iacobelli S, Hoek J, Mikulska M, Aljurf M, Gil L, Cesaro S. Protective environment for hematopoietic cell transplant (HSCT) recipients: The Infectious Diseases Working Party EBMT

analysis of global recommendations on health-care facilities. Bone Marrow Transplant. 2018 Sep;53(9):1131-8. DOI: 10.1038/s41409-018-0141-5
215. DIN 1946-4:2018-09. Raumlufttechnik – Teil 4: Raumlufttechnische Anlagen in Gebäuden und Räumen des Gesundheitswesens. Berlin: Beuth.
216. Maschmeyer G, Neuburger S, Fritz L, Böhme A, Penack O, Schwerdtfeger R, Buchheidt D, Ludwig WD; Infectious Diseases Working Party (AGIHO) of the German Society of Haematology and Oncology. A prospective, randomised study on the use of well-fitting masks for prevention of invasive aspergillosis in high-risk patients. Ann Oncol. 2009 Sep;20(9):1560-4. DOI: 10.1093/annonc/mdp034
217. Raad I, Hanna H, Osting C, Hachem R, Umphrey J, Tarrand J, Kantarjian H, Bodey GP. Masking of neutropenic patients on transport from hospital rooms is associated with a decrease in nosocomial aspergillosis during construction. Infect Control Hosp Epidemiol. 2002 Jan;23(1):41-3. DOI: 10.1086/501967
218. Verein Deutscher Ingenieure e.V. (VDI). Richtlinienreihe VDI 6022 „Raumlufttechnik, Raumluftqualität“. [cited 2020 Nov 01]. Available from: https://www.vdi.de/richtlinien/unsere-richtlinien-highlights/vdi-6022
219. Göttlich E, Engesser K, Bardtke D. Emission von Pilzsporen in Müllverarbeitungsanlagen. Forum Städte-Hygiene. 1994;45(11/12):321-5.
220. Dyck A, Exner M, Kramer A. Experimental based experiences with the introduction of a water safety plan for a multi-located university clinic and its efficacy according to WHO recommendations. BMC Public Health. 2007 Mar;7:34. DOI: 10.1186/1471-2458-7-34
221. Kizny Gordon AE, Mathers AJ, Cheong EYL, Gottlieb T, Kotay S, Walker AS, Peto TEA, Crook DW, Stoesser N. The Hospital Water Environment as a Reservoir for Carbapenem-Resistant Organisms Causing Hospital-Acquired Infections-A Systematic Review of the Literature. Clin Infect Dis. 2017 May;64(10):1435-44. DOI: 10.1093/cid/cix132
222. Kossow A, Kampmeier S, Willems S, Berdel WE, Groll AH, Burkhardt B, Rossig C, Groth C, Idelevich EA, Kipp F, Mellmann A, Stelljes M. Control of Multidrug-Resistant Pseudomonas aeruginosa in Allogeneic Hematopoietic Stem Cell Transplant Recipients by a Novel Bundle Including Remodeling of Sanitary and Water Supply Systems. Clin Infect Dis. 2017 Sep;65(6):935-42. DOI: 10.1093/cid/cix465
223. Baranovsky S, Jumas-Bilak E, Lotthé A, Marchandin H, Parer S, Hicheri Y, Romano-Bertrand S. Tracking the spread routes of opportunistic premise plumbing pathogens in a haematology unit with water points-of-use protected by antimicrobial filters. J Hosp Infect. 2018 Jan;98(1):53-9. DOI: 10.1016/j.jhin.2017.07
224. Garvey MI, Bradley CW, Holden E. Waterborne Pseudomonas aeruginosa transmission in a hematology unit? Am J Infect Control. 2018;46(4):383-6.
225. Kommission für Krankenhaushygiene und Infektionsprävention (KRINKO). Anforderungen der Hygiene an abwasserführende Systeme in medizinischen Einrichtungen. Bundesgesundheitsbl. 2020;63(4):484-501.
226. Charron D, Bédard E, Lalancette C, Laferrière C, Prévost M. Impact of electronic faucets and water quality on the occurrence of Pseudomonas aeruginosa in water: a multi-hospital study. Infect Control Hosp Epidemiol. 2015 Mar;36(3):311-9. DOI: 10.1017/ice.2014.46
227. Schneider H, Geginat G, Hogardt M, Kramer A, Dürken M, Schroten H, Tenenbaum T. Pseudomonas aeruginosa outbreak in a pediatric oncology care unit caused by an errant water jet into contaminated siphons. Pediatr Infect Dis J. 2012 Jun;31(6):648-50. DOI: 10.1097/INF.0b013e31824d1a11
228. Picot-Guéraud R, Khouri C, Brenier-Pinchart MP, Saviuc P, Fares A, Sellon T, Thiebaut-Bertrand A, Mallaret MR. En-suite bathrooms in protected haematology wards: a source of filamentous fungal contamination? J Hosp Infect. 2015 Nov;91(3):244-9. DOI: 10.1016/j.jhin.2015.07.005
229. Brown L, Siddiqui S, McMullen A, Waller J, Baer S. Revisiting the “leading edge” of hospital privacy curtains in the medical intensive care unit. Am J Infect Control. 2020 Jul;48(7):746-0. DOI: 10.1016/j.ajic.2020.03.015
230. Larocque M, Carver S, Bertrand A, McGeer A, McLeod S, Borgundvaag B. Acquisition of bacteria on health care workers’ hands after contact with patient privacy curtains. Am J Infect Control. 2016 Nov;44(11):1385-6. DOI: 10.1016/j.ajic.2016.04.227
231. Shek K, Patidar R, Kohja Z, Liu S, Gawaziuk JP, Gawthrop M, Kumar A, Logsetty S. Rate of contamination of hospital privacy curtains on a burns and plastic surgery ward: a cross-sectional study. J Hosp Infect. 2017 May;96(1):54-8. DOI: 10.1016/j.jhin.2017.03.012
232. Wilson G, Jackson V, Boyken L, Puig-Asensio M, Marra AR, Perencevich E, Schweizer ML, Diekema D, Breheny P, Petersen C. A randomized control trial evaluating efficacy of antimicrobial impregnated hospital privacy curtains in an intensive care setting. Am J Infect Control. 2020 Aug;48(8):862-8. DOI: 10.1016/j.ajic.2019.12.024
233. Garvey MI, Wilkinson MAC, Holden KL, Martin T, Parkes J, Holden E. Tap out: reducing waterborne Pseudomonas aeruginosa transmission in an intensive care unit. J Hosp Infect. 2019;102(1):75-81.
234. Francois Watkins LK, Toews KE, Harris AM, Davidson S, Ayers-Millsap S, Lucas CE, Hubbard BC, Kozak-Muiznieks NA, Khan E, Kutty PK. Lessons From an Outbreak of Legionnaires’ Disease on a Hematology-Oncology Unit. Infect Control Hosp Epidemiol. 2017 Mar;38(3):306-13. DOI: 10.1017/ice.2016.281
235. Micol JB, de Botton S, Guieze R, Coiteux V, Darre S, Dessein R, Leroy O, Yakoub-Agha I, Quesnel B, Bauters F, Beaucaire G, Alfandari S. An 18-case outbreak of drug-resistant Pseudomonas aeruginosa bacteriemia in hematology patients. Haematologica. 2006 Aug;91(8):1134-8.
236. Vianelli N, Giannini MB, Quarti C, Bucci Sabattini MA, Fiacchini M, de Vivo A, Graldi P, Galli S, Nanetti A, Baccarani M, Ricci P. Resolution of a Pseudomonas aeruginosa outbreak in a hematology unit with the use of disposable sterile water filters. Haematologica. 2006 Jul;91(7):983-5.

237. De Brabandere E, Ablorh R, Leroux-Roels I. The hospital sanitary as a source of a vim-producing multidrug resistant pseudomonas aeruginosa outbreak at the pediatric hemato-oncology ward. Antimicrob Resist Infect Control. 2017;6(Suppl 3):52.
238. Walker J, Moore G. Safe water in healthcare premises. J Hosp Infect. 2016 Sep;94(1):1. DOI: 10.1016/j.jhin.2016.07.001
239. Garvey MI, Bradley CW, Jumaa P. The risks of contamination from tap end filters. J Hosp Infect. 2016 Nov;94(3):282-3. DOI: 10.1016/j.jhin.2016.08.006
240. Eckmanns T, Oppert M, Martin M, Amorosa R, Zuschneid I, Frei U, Rüden H, Weist K. An outbreak of hospital-acquired Pseudomonas aeruginosa infection caused by contaminated bottled water in intensive care units. Clin Microbiol Infect. 2008 May;14(5):454-8. DOI: 10.1111/j.1469-0691.2008.01949.x
241. Wilson C, Dettenkofer M, Jonas D, Daschner FD. Pathogen growth in herbal teas used in clinical settings: a possible source of nosocomial infection? Am J Infect Control. 2004 Apr;32(2):117-9. DOI: 10.1016/j.ajic.2003.09.004
242. Kanamori H, Rutala WA, Sickbert-Bennett EE, Weber DJ. Review of fungal outbreaks and infection prevention in healthcare settings during construction and renovation. Clin Infect Dis. 2015 Aug;61(3):433-44. DOI: 10.1093/cid/civ297
243. Pokala HR, Leonard D, Cox J, Metcalf P, McClay J, Siegel J, Winick N. Association of hospital construction with the development of healthcare associated environmental mold infections (HAEMI) in pediatric patients with leukemia. Pediatr Blood Cancer. 2014 Feb;61(2):276-80. DOI: 10.1002/pbc.24685
244. Talento AF, Fitzgerald M, Redington B, O'Sullivan N, Fenelon L, Rogers TR. Prevention of healthcare-associated invasive aspergillosis during hospital construction/renovation works. J Hosp Infect. 2019;103(1):1-12.
245. Berger J, Willinger B, Diab-Elschahawi M, Blacky A, Kalhs P, Koller W, Assadian O, Aichberger KJ. Effectiveness of preventive measures for hemato-oncologic patients undergoing stem cell transplantation during a period of hospital construction. Am J Infect Control. 2011 Nov;39(9):746-51. DOI: 10.1016/j.ajic.2011.01.011
246. Mellinghoff SC, Panse J, Alakel N, Behre G, Buchheidt D, Christopeit M, Hasenkamp J, Kiehl M, Koldehoff M, Krause SW, Lehners N, von Lilienfeld-Toal M, Löhnert AY, Maschmeyer G, Teschner D, Ullmann AJ, Penack O, Ruhnke M, Mayer K, Ostermann H, Wolf HH, Cornely OA. Primary prophylaxis of invasive fungal infections in patients with haematological malignancies: 2017 update of the recommendations of the Infectious Diseases Working Party (AGIHO) of the German Society for Haematology and Medical Oncology (DGHO). Ann Hematol. 2018 Feb;97(2):197-207. DOI: 10.1007/s00277-017-3196-2
247. Lehrnbecher T. Antifungal prophylaxis in pediatric patients undergoing therapy for cancer: drugs and dosing. Curr Opin Infect Dis. 2015 Dec;28(6):523-31. DOI: 10.1097/QCO.0000000000000210
248. Yunus S, Pieper S, Kolve H, Goletz G, Jurgens H, Groll AH. Azole-based chemoprophylaxis of invasive fungal infections in paediatric patients with acute leukaemia: an internal audit. J Antimicrob Chemother. 2014;69(3):815-20.
249. Tragiannidis A, Dokos C, Lehrnbecher T, Groll AH. Antifungal chemoprophylaxis in children and adolescents with haematological malignancies and following allogeneic haematopoietic stem cell transplantation: review of the literature and options for clinical practice. Drugs. 2012 Mar;72(5):685-704. DOI: 10.2165/11599810-000000000-00000
250. Lehrnbecher T, Fisher BT, Phillips B, Beauchemin M, Carlesse F, Castagnola E, Duong N, Dupuis LL, Fioravantti V, Groll AH, Haeusler GM, Roilides E, Science M, Steinbach WJ, Tissing W, Warris A, Patel P, Robinson PD, Sung L. Clinical Practice Guideline for Systemic Antifungal Prophylaxis in Pediatric Patients With Cancer and Hematopoietic Stem-Cell Transplantation Recipients. J Clin Oncol. 2020 Sep;38(27):3205-16. DOI: 10.1200/JCO.20.00158
251. Rhame FS. Prevention of nosocomial aspergillosis. J Hosp Infect. 1991 Jun;18 Suppl A:466-72. DOI: 10.1016/0195-6701(91)90058-g
252. Streifel AJ, Lauer JL, Vesley D, Juni B, Rhame FS. Aspergillus fumigatus and other thermotolerant fungi generated by hospital building demolition. Appl Environ Microbiol. 1983 Aug;46(2):375-8. DOI: 10.1128/aem.46.2.375-378.1983
253. Vonberg RP, Gastmeier P. Nosocomial aspergillosis in outbreak settings. J Hosp Infect. 2006 Jul;63(3):246-54. DOI: 10.1016/j.jhin.2006.02.014
254. Chang CC, Ananda-Rajah M, Belcastro A, McMullan B, Reid A, Dempsey K, Athan E, Cheng AC, Slavin MA. Consensus guidelines for implementation of quality processes to prevent invasive fungal disease and enhanced surveillance measures during hospital building works, 2014. Intern Med J. 2014 Dec;44(12b):1389-97. DOI: 10.1111/imj.12601
255. Combariza JF, Toro LF, Orozco JJ, Arango M. Cost-effectiveness analysis of interventions for prevention of invasive aspergillosis among leukemia patients during hospital construction activities. Eur J Haematol. 2018 Feb;100(2):140-6. DOI: 10.1111/ejh.12991
256. Manuel RJ, Kibbler CC. The epidemiology and prevention of invasive aspergillosis. J Hosp Infect. 1998 Jun;39(2):95-109. DOI: 10.1016/s0195-6701(98)90323-1
257. Mahieu LM, De Dooy JJ, Van Laer FA, Jansens H, Ieven MM. A prospective study on factors influencing aspergillus spore load in the air during renovation works in a neonatal intensive care unit. J Hosp Infect. 2000;45(3):191-7.
258. Barnes RA, Rogers TR. Control of an outbreak of nosocomial aspergillosis by laminar air-flow isolation. J Hosp Infect. 1989 Aug;14(2):89-94. DOI: 10.1016/0195-6701(89)90110-2
259. Klimowski LL, Rotstein C, Cummings KM. Incidence of nosocomial aspergillosis in patients with leukemia over a twenty-year period. Infect Control Hosp Epidemiol. 1989;10(7):299-305.

260. Antoniadou A. Outbreaks of zygomycosis in hospitals. Clin Microbiol Infect. 2009 Oct;15 Suppl 5:55-9. DOI: 10.1111/j.1469-0691.2009.02982.x
261. Construction-related nosocomial infections in patients in health care facilities. Decreasing the risk of Aspergillus, Legionella and other infections. Can Commun Dis Rep. 2001 Jul;27 Suppl 2:i-x, 1-42, i-x, 1-46.
262. Kommission für Krankenhaushygiene und Infektionsprävention (KRINKO). Prävention und Kontrolle Katheter-assoziierter Harnwegsinfektionen. Bundesgesundheitsbl. 2015;58(6):641-50.
263. Kommission für Krankenhaushygiene und Infektionsprävention (KRINKO). Prävention postoperativer Wundinfektionen. Bundesgesundheitsbl. 2018;61(4):448-73.
264. Tomsic I, Heinze NR, Chaberny IF, Krauth C, Schock B, von Lengerke T. Implementation interventions in preventing surgical site infections in abdominal surgery: a systematic review. BMC Health Serv Res. 2020 Mar;20(1):236. DOI: 10.1186/s12913-020-4995-z
265. Poutsiaka DD, Munson D, Price LL, Chan GW, Snydman DR. Blood stream infection (BSI) and acute GVHD after hematopoietic SCT (HSCT) are associated. Bone Marrow Transplant. 2011 Feb;46(2):300-7. DOI: 10.1038/bmt.2010.112
266. Dandoy CE, Alonso PB. MBI-LCBI and CLABSI: more than scrubbing the line. Bone Marrow Transplant. 2019 Dec;54(12):1932-9. DOI: 10.1038/s41409-019-0489-1
267. Balian C, Garcia M, Ward J. A Retrospective Analysis of Bloodstream Infections in Pediatric Allogeneic Stem Cell Transplant Recipients: The Role of Central Venous Catheters and Mucosal Barrier Injury. J Pediatr Oncol Nurs. 2018 May;35(3):210-7. DOI: 10.1177/1043454218762706
268. Dandoy CE, Haslam D, Lane A, Jodele S, Demmel K, El-Bietar J, Flesch L, Myers KC, Pate A, Rotz S, Daniels P, Wallace G, Nelson A, Waters H, Connelly B, Davies SM. Healthcare Burden, Risk Factors, and Outcomes of Mucosal Barrier Injury Laboratory-Confirmed Bloodstream Infections after Stem Cell Transplantation. Biol Blood Marrow Transplant. 2016 Sep;22(9):1671-7. DOI: 10.1016/j.bbmt.2016.06.002
269. Lukenbill J, Rybicki L, Sekeres MA, Zaman MO, Copelan A, Haddad H, Fraser T, DiGiorgio MJ, Hanna R, Duong H, Hill B, Kalaycio M, Sobecks R, Bolwell B, Copelan E. Defining incidence, risk factors, and impact on survival of central line-associated blood stream infections following hematopoietic cell transplantation in acute myeloid leukemia and myelodysplastic syndrome. Biol Blood Marrow Transplant. 2013 May;19(5):720-4. DOI: 10.1016/j.bbmt.2013.01.022
270. Mikulska M, Viscoli C, Orasch C, Livermore DM, Averbuch D, Cordonnier C, Akova M; Fourth European Conference on Infections in Leukemia Group (ECIL-4), a joint venture of EBMT, EORTC, ICHS, ELN and ESGICH/ESCMID. Aetiology and resistance in bacteraemias among adult and paediatric haematology and cancer patients. J Infect. 2014 Apr;68(4):321-31. DOI: 10.1016/j.jinf.2013.12.006
271. Metzger KE, Rucker Y, Callaghan M, Churchill M, Jovanovic BD, Zembower TR, Bolon MK. The burden of mucosal barrier injury laboratory-confirmed bloodstream infection among hematology, oncology, and stem cell transplant patients. Infect Control Hosp Epidemiol. 2015 Feb;36(2):119-24. DOI: 10.1017/ice.2014.38
272. See I, Iwamoto M, Allen-Bridson K, Horan T, Magill SS, Thompson ND. Mucosal barrier injury laboratory-confirmed bloodstream infection: results from a field test of a new National Healthcare Safety Network definition. Infect Control Hosp Epidemiol. 2013 Aug;34(8):769-76. DOI: 10.1086/671281
273. Stango C, Runyan D, Stern J, Macri I, Vacca M. A successful approach to reducing bloodstream infections based on a disinfection device for intravenous needleless connector hubs. J Infus Nurs. 2014 Nov-Dec;37(6):462-5. DOI: 10.1097/NAN.0000000000000075
274. Kamboj M, Blair R, Bell N, Son C, Huang YT, Dowling M, Lipitz-Snyderman A, Eagan J, Sepkowitz K. Use of Disinfection Cap to Reduce Central-Line-Associated Bloodstream Infection and Blood Culture Contamination Among Hematology-Oncology Patients. Infect Control Hosp Epidemiol. 2015 Dec;36(12):1401-8. DOI: 10.1017/ice.2015.219
275. Timsit JF, Mimoz O, Mourvillier B, Souweine B, Garrouste-Orgeas M, Alfandari S, Plantefeve G, Bronchard R, Troche G, Gauzit R, Antona M, Canet E, Bohe J, Lepape A, Vesin A, Arrault X, Schwebel C, Adrie C, Zahar JR, Ruckly S, Tournegros C, Lucet JC. Randomized controlled trial of chlorhexidine dressing and highly adhesive dressing for preventing catheter-related infections in critically ill adults. Am J Respir Crit Care Med. 2012 Dec;186(12):1272-8. DOI: 10.1164/rccm.201206-1038OC
276. Timsit JF, Schwebel C, Bouadma L, Geffroy A, Garrouste-Orgeas M, Pease S, Herault MC, Haouache H, Calvino-Gunther S, Gestin B, Armand-Lefevre L, Leflon V, Chaplain C, Benali A, Francais A, Adrie C, Zahar JR, Thuong M, Arrault X, Croize J, Lucet JC; Dressing Study Group. Chlorhexidine-impregnated sponges and less frequent dressing changes for prevention of catheter-related infections in critically ill adults: a randomized controlled trial. JAMA. 2009 Mar;301(12):1231-41. DOI: 10.1001/jama.2009.376
277. Ruschulte H, Franke M, Gastmeier P, Zenz S, Mahr KH, Buchholz S, Hertenstein B, Hecker H, Piepenbrock S. Prevention of central venous catheter related infections with chlorhexidine gluconate impregnated wound dressings: a randomized controlled trial. Ann Hematol. 2009 Mar;88(3):267-72. DOI: 10.1007/s00277-008-0568-7
278. van der Velden WJ, Herbers AH, Netea MG, Blijlevens NM. Mucosal barrier injury, fever and infection in neutropenic patients with cancer: introducing the paradigm febrile mucositis. Br J Haematol. 2014 Nov;167(4):441-52. DOI: 10.1111/bjh.13113
279. Zecha JAEM, Raber-Durlacher JE, Laheij AMGA, Westermann AM, Epstein JB, de Lange J, Smeele LE. The impact of the oral cavity in febrile neutropenia and infectious complications in patients treated with myelosuppressive chemotherapy. Support Care Cancer. 2019 Oct;27(10):3667-79. DOI: 10.1007/s00520-019-04925-8

280. Schmalz G, Tulani L, Busjan R, Haak R, Kottmann T, Trümper L, Hasenkamp J, Ziebolz D. Dental and Periodontal Treatment Need after Dental Clearance Is Not Associated with the Outcome of Induction Therapy in Patients with Acute Leukemia: Results of a Retrospective Pilot Study. Adv Hematol. 2020 Apr 21;2020:6710906. DOI: 10.1155/2020/6710906
281. Carvalho CG, Medeiros-Filho JB, Ferreira MC. Guide for health professionals addressing oral care for individuals in oncological treatment based on scientific evidence. Support Care Cancer. 2018 Aug;26(8):2651-61. DOI: 10.1007/s00520-018-4111-7
282. Lalla RV, Bowen J, Barasch A, Elting L, Epstein J, Keefe DM, McGuire DB, Migliorati C, Nicolatou-Galitis O, Peterson DE, Raber-Durlacher JE, Sonis ST, Elad S; Mucositis Guidelines Leadership Group of the Multinational Association of Supportive Care in Cancer and International Society of Oral Oncology (MASCC/ISOO). MASCC/ISOO clinical practice guidelines for the management of mucositis secondary to cancer therapy. Cancer. 2014 May 15;120(10):1453-61. DOI: 10.1002/cncr.28592
283. Peterson DE, Boers-Doets CB, Bensadoun RJ, Herrstedt J; ESMO Guidelines Committee. Management of oral and gastrointestinal mucosal injury: ESMO Clinical Practice Guidelines for diagnosis, treatment, and follow-up. Ann Oncol. 2015 Sep;26 Suppl 5:v139-51. DOI: 10.1093/annonc/mdv202
284. Mutters NT, Neubert TR, Nieth R, Mutters R. The role of Octenidol(®), Glandomed(®) and chlorhexidine mouthwash in the prevention of mucositis and in the reduction of the oropharyngeal flora: a double-blind randomized controlled trial. GMS Hyg Infect Control. 2015;10:Doc05. DOI: 10.3205/dgkh000248
285. Cardona A, Balouch A, Abdul MM, Sedghizadeh PP, Enciso R. Efficacy of chlorhexidine for the prevention and treatment of oral mucositis in cancer patients: a systematic review with meta-analyses. J Oral Pathol Med. 2017 Oct;46(9):680-8. DOI: 10.1111/jop.12549
286. Lemes LG, Corrêa TS, Fiaccadori FS, Cardoso Dd, Arantes Ade M, Souza KM, Souza M. Prospective study on Norovirus infection among allogeneic stem cell transplant recipients: prolonged viral excretion and viral RNA in the blood. J Clin Virol. 2014 Nov;61(3):329-33. DOI: 10.1016/j.jcv.2014.08.004
287. Sheahan A, Copeland G, Richardson L, McKay S, Chou A, Babady NE, Tang YW, Boulad F, Eagan J, Sepkowitz K, Kamboj M. Control of norovirus outbreak on a pediatric oncology unit. Am J Infect Control. 2015 Oct;43(10):1066-9. DOI: 10.1016/j.ajic.2015.05.032
288. Ye X, Van JN, Munoz FM, Revell PA, Kozinetz CA, Krance RA, Atmar RL, Estes MK, Koo HL. Noroviruses as a Cause of Diarrhea in Immunocompromised Pediatric Hematopoietic Stem Cell and Solid Organ Transplant Recipients. Am J Transplant. 2015 Jul;15(7):1874-81. DOI: 10.1111/ajt.13227
289. Echenique IA, Penugonda S, Stosor V, Ison MG, Angarone MP. Diagnostic yields in solid organ transplant recipients admitted with diarrhea. Clin Infect Dis. 2015 Mar;60(5):729-37. DOI: 10.1093/cid/ciu880
290. Kamboj M, Mihu CN, Sepkowitz K, Kernan NA, Papanicolaou GA. Work-up for infectious diarrhea after allogeneic hematopoietic stem cell transplantation: single specimen testing results in cost savings without compromising diagnostic yield. Transpl Infect Dis. 2007 Dec;9(4):265-9. DOI: 10.1111/j.1399-3062.2007.00230.x
291. Trinh SA, Echenique IA, Penugonda S, Angarone MP. Optimal strategies for the diagnosis of community-onset diarrhea in solid organ transplant recipients: Less is more. Transpl Infect Dis. 2017;19(2):e12673. DOI: 10.1111/tid.12673
292. Chadwick PR, Beards G, Brown D, Caul EO, Cheesbrough J, Clarke I, Curry A, O'Brien S, Quigley K, Sellwood J, Westmoreland D. Management of hospital outbreaks of gastro-enteritis due to small roundstructured viruses. J Hosp Infect. 2000 May;45(1):1-10. DOI: 10.1053/jhin.2000.0662
293. Robert Koch-Institut (RKI). Norovirus-Gastroenteritiden haben in den letzten Wochen deutlich zugenommen – steht eine neue Winterepidemie bevor? Epid Bull. 2006;48:427-9.
294. Robert Koch-Institut (RKI). RKI-Ratgeber. Rotaviren-Gastroenteritis. 2019 [cited 2020 Nov 01]. Available from: https://www.rki.de/DE/Content/Infekt/EpidBull/Merkblaetter/Ratgeber_Rotaviren.html
295. Kleinkauf N, Eckmanns T, Robert Koch-Institut (RKI). Clostridium difficile: Zum Stand der Meldungen schwer verlaufender Infektionen in Deutschland. Epid Bull. 2008;15:117-9.
296. Daniel-Wayman S, Fahle G, Palmore T, Green KY, Prevots DR. Norovirus, astrovirus, and sapovirus among immunocompromised patients at a tertiary care research hospital. Diagn Microbiol Infect Dis. 2018;92(2):143-6.
297. Green KY. Norovirus infection in immunocompromised hosts. Clin Microbiol Infect. 2014 Aug;20(8):717-23. DOI: 10.1111/1469-0691.12761
298. Kamboj M, Son C, Cantu S, Chemaly RF, Dickman J, Dubberke E, Engles L, Lafferty T, Liddell G, Lesperance ME, Mangino JE, Martin S, Mayfield J, Mehta SA, O'Rourke S, Perego CS, Taplitz R, Eagan J, Sepkowitz KA. Hospital-onset Clostridium difficile infection rates in persons with cancer or hematopoietic stem cell transplant: a C3IC network report. Infect Control Hosp Epidemiol. 2012 Nov;33(11):1162-5. DOI: 10.1086/668023
299. Kamboj M, Sheahan A, Sun J, Taur Y, Robilotti E, Babady E, Papanicolaou G, Jakubowski A, Pamer E, Sepkowitz K. Transmission of Clostridium difficile During Hospitalization for Allogeneic Stem Cell Transplant. Infect Control Hosp Epidemiol. 2016 Jan;37(1):8-15. DOI: 10.1017/ice.2015.237
300. Plößer P. Clostridium difficile: Nachweis von Ribotyp 027 in Deutschschland – Clostridium difficile im Überblick - Hygienemaßnahmen. Hyg Med. 2007;32(10):403-5.
301. Schneider T, Eckmanns T, Ignatius R, Weist K, Liesenfeld O. Clostridium-difficile-assoziierte Diarrhö. Dtsch Arztebl. 2007;104(22):1588-94.
302. Boyle NM, Magaret A, Stednick Z, Morrison A, Butler-Wu S, Zerr D, Rogers K, Podczervinski S, Cheng A, Wald A, Pergam SA. Evaluating risk factors for Clostridium difficile infection in adult and pediatric hematopoietic cell transplant recipients. Antimicrob Resist Infect Control. 2015;4:41. DOI: 10.1186/s13756-015-0081-4

303. Bruminhent J, Wang ZX, Hu C, Wagner J, Sunday R, Bobik B, Hegarty S, Keith S, Alpdogan S, Carabasi M, Filicko-O'Hara J, Flomenberg N, Kasner M, Outschoorn UM, Weiss M, Flomenberg P. Clostridium difficile colonization and disease in patients undergoing hematopoietic stem cell transplantation. Biol Blood Marrow Transplant. 2014 Sep;20(9):1329-34. DOI: 10.1016/j.bbmt.2014.04.026
304. Kinnebrew MA, Lee YJ, Jenq RR, Lipuma L, Littmann ER, Gobourne A, No D, van den Brink M, Pamer EG, Taur Y. Early Clostridium difficile infection during allogeneic hematopoietic stem cell transplantation. PLoS One. 2014;9(3):e90158. DOI: 10.1371/journal.pone.0090158
305. Simon A, Mock M, Graf N, von Muller L. Investigation of Clostridium difficile ribotypes in symptomatic patients of a German pediatric oncology center. Eur J Pediatr. 2018;177(3):403-8.
306. Salamonowicz M, Ociepa T, Frączkiewicz J, Szmydki-Baran A, Matysiak M, Czyżewski K, Wysocki M, Gałązka P, Zalas-Więcek P, Irga-Jaworska N, Drożyńska E, Zając-Spychała O, Wachowiak J, Gryniewicz-Kwiatkowska O, Czajńska-Deptuła A, Dembowska-Bagińska B, Chełmecka-Wiktorczyk L, Balwierz W, Bartnik M, Zielezińska K, Urasiński T, Tomaszewska R, Szczepański T, Płonowski M, Krawczuk-Rybak M, Pierlejewski F, Młynarski W, Gamrot-Pyka Z, Woszczyk M, Małas Z, Badowska W, Urbanek-Dądela A, Karolczyk G, Stolpa W, Sobol-Milejska G, Zaucha-Prażmo A, Kowalczyk J, Goździk J, Gorczyńska E, Jermakow K, Król A, Chybicka A, Ussowicz M, Kałwak K, Styczyński J. Incidence, course, and outcome of Clostridium difficile infection in children with hematological malignancies or undergoing hematopoietic stem cell transplantation. Eur J Clin Microbiol Infect Dis. 2018 Sep;37(9):1805-12. DOI: 10.1007/s10096-018-3316-5
307. Risi GF, Tomascak V. Prevention of infection in the immunocompromised host. Am J Infect Control. 1998 Dec;26(6):594-604; quiz 605-6. DOI: 10.1053/ic.1998.v26.a89371
308. McCullough A, Ruehrdanz A, Jenkins MA, Gilmer MJ, Olson J, Pawar A, Holley L, Sierra-Rivera S, Linder DE, Pichette D, Grossman NJ, Hellman C, Guérin NA, O'Haire ME. Measuring the Effects of an Animal-Assisted Intervention for Pediatric Oncology Patients and Their Parents: A Multisite Randomized Controlled Trial [Formula: see text]. J Pediatr Oncol Nurs. 2018 May;35(3):159-77. DOI: 10.1177/1043454217748586
309. Schmitz A, Beermann M, MacKenzie CR, Fetz K, Schulz-Quach C. Animal-assisted therapy at a University Centre for Palliative Medicine – a qualitative content analysis of patient records. BMC Palliat Care. 2017;16(1):50.
310. Ariza-Heredia EJ, Kontoyiannis DP. Our recommendations for avoiding exposure to fungi outside the hospital for patients with haematological cancers. Mycoses. 2014 Jun;57(6):336-41. DOI: 10.1111/myc.12167
311. Böhme H, Fruth A, Rabsch W. Reptilien-assoziierte Salmonelleninfektionen bei Säuglingen und Kleinkindern in Deutschland. Klin Padiatr. 2009;221(2):60-4.
312. Boost MV, O'Donoghue MM, Siu KH. Characterisation of methicillin-resistant Staphylococcus aureus isolates from dogs and their owners. Clin Microbiol Infect. 2007 Jul;13(7):731-3. DOI: 10.1111/j.1469-0691.2007.01737.x
313. Gabriels P, Joosen H, Put E, Verhaegen J, Magerman K, Cartuyvels R. Recurrent Rhodococcus equi infection with fatal outcome in an immunocompetent patient. Eur J Clin Microbiol Infect Dis. 2006;25(1):46-8.
314. Harris JR, Neil KP, Behravesh CB, Sotir MJ, Angulo FJ. Recent multistate outbreaks of human salmonella infections acquired from turtles: a continuing public health challenge. Clin Infect Dis. 2010 Feb;50(4):554-9. DOI: 10.1086/649932
315. Morris DO, Lautenbach E, Zaoutis T, Leckerman K, Edelstein PH, Rankin SC. Potential for pet animals to harbour methicillin-resistant Staphylococcus aureus when residing with human MRSA patients. Zoonoses Public Health. 2012 Jun;59(4):286-93. DOI: 10.1111/j.1863-2378.2011.01448.x
316. Simon A. Umgang mit Tierkontakten bei immunsupprimierten Kindern. Hyg Med. 2013;38(7/8):321-3.
317. Kommission für Krankenhaushygiene und Infektionsprävention (KRINKO). Surveillance von nosokomialen Infektionen. Empfehlung der Kommission für Krankenhaushygiene und Infektionsprävention (KRINKO) beim Robert Koch-Institut. Bundesgesundheitsbl. 2020;63(2):228-41.
318. Bearman G, Doll M, Cooper K, Stevens MP. Hospital Infection Prevention: How Much Can We Prevent and How Hard Should We Try? Curr Infect Dis Rep. 2019 Feb;21(1):2. DOI: 10.1007/s11908-019-0660-2
319. Horowitz HW. Infection control IV: Moving forward- infection preventionists' scope of practice. Am J Infect Control. 2018 Jul;46(7):734-5. DOI: 10.1016/j.ajic.2018.02.030
320. Vokes RA, Bearman G, Bazzoli GJ. Hospital-Acquired Infections Under Pay-for-Performance Systems: an Administrative Perspective on Management and Change. Curr Infect Dis Rep. 2018 Jul;20(9):35. DOI: 10.1007/s11908-018-0638-5
321. Horowitz HW. Infection control: Public reporting, disincentives, and bad behavior. Am J Infect Control. 2015 Sep;43(9):989-91. DOI: 10.1016/j.ajic.2015.02.033
322. Horowitz HW. Infection control II: A practical guide to getting to zero. Am J Infect Control. 2016;44(9):1075-7. DOI: 10.1016/j.ajic.2016.02.032
323. Williams MR, Costa SK, Zaramela LS, Khalil S, Todd DA, Winter HL, Sanford JA, O'Neill AM, Liggins MC, Nakatsuji T, Cech NB, Cheung AL, Zengler K, Horswill AR, Gallo RL. Quorum sensing between bacterial species on the skin protects against epidermal injury in atopic dermatitis. Sci Transl Med. 2019 May 1;11(490):eaat8329. DOI: 10.1126/scitranslmed.aat8329
324. Kommission für Krankenhaushygiene und Infektionsprävention (KRINKO). Mitteilungen der Kommission für Krankenhaushygiene und Infektionsprävention zur Surveillance (Erfassung und Bewertung) von nosokomialen Infektionen (Umsetzung §23 IfSG). Bundesgesundheitsbl. 2001;44(5):523-36.
325. Ammann RA, Laws HJ, Schrey D, Ehlert K, Moser O, Dilloo D, Bode U, Wawer A, Schrauder A, Cario G, Laengler A, Graf N, Furtwängler R, Simon A. Bloodstream infection in paediatric cancer centres--leukaemia and relapsed malignancies are independent risk factors. Eur J Pediatr. 2015 May;174(5):675-86. DOI: 10.1007/s00431-015-2525-5

326. Dettenkofer M, Ebner W, Bertz H, Babikir R, Finke J, Frank U, Rüden H, Daschner FD. Surveillance of nosocomial infections in adult recipients of allogeneic and autologous bone marrow and peripheral blood stem-cell transplantation. Bone Marrow Transplant. 2003 May;31(9):795-801. DOI: 10.1038/sj.bmt.1703920
327. Dettenkofer M, Wenzler-Röttele S, Babikir R, Bertz H, Ebner W, Meyer E, Rüden H, Gastmeier P, Daschner FD; Hospital Infection Surveillance System for Patients with Hematologic/Oncologic Malignancies Study Group. Surveillance of nosocomial sepsis and pneumonia in patients with a bone marrow or peripheral blood stem cell transplant: a multicenter project. Clin Infect Dis. 2005 Apr;40(7):926-31. DOI: 10.1086/428046
328. Simon A, Fleischhack G, Hasan C, Bode U, Engelhart S, Kramer MH. Surveillance for nosocomial and central line-related infections among pediatric hematology-oncology patients. Infect Control Hosp Epidemiol. 2000 Sep;21(9):592-6. DOI: 10.1086/501809
329. Simon A, Fleischhack G. Surveillance nosokomialer Infektionen in der pädiatrischen Hämatologie/Onkologie. Klin Padiatr. 2001;213(S1):A106-13.
330. Simon A, Furtwängler R, Graf N, Laws HJ, Voigt S, Piening B, Geffers C, Agyeman P, Ammann RA. Surveillance of bloodstream infections in pediatric cancer centers - what have we learned and how do we move on? GMS Hyg Infect Control. 2016;11:Doc11. DOI: 10.3205/dgkh000271
331. Fraser TG, Gordon SM. CLABSI rates in immunocompromised patients: a valuable patient centered outcome? Clin Infect Dis. 2011 Jun;52(12):1446-50. DOI: 10.1093/cid/cir200
332. Sexton DJ, Chen LF, Anderson DJ. Current definitions of central line-associated bloodstream infection: is the emperor wearing clothes? Infect Control Hosp Epidemiol. 2010 Dec;31(12):1286-9. DOI: 10.1086/657583
333. Tamburini FB, Andermann TM, Tkachenko E, Senchyna F, Banaei N, Bhatt AS. Precision identification of diverse bloodstream pathogens in the gut microbiome. Nat Med. 2018 Dec;24(12):1809-14. DOI: 10.1038/s41591-018-0202-8
334. Gyarmati P, Kjellander C, Aust C, Kalin M, Öhrmalm L, Giske CG. Bacterial Landscape of Bloodstream Infections in Neutropenic Patients via High Throughput Sequencing. PLoS One. 2015;10(8):e0135756. DOI: 10.1371/journal.pone.0135756
335. Gopalakrishnan V, Jenq RR. Implicating or exonerating the gut microbiome in blood-borne infection. Nat Med. 2018 Dec;24(12):1788-9. DOI: 10.1038/s41591-018-0270-9
336. Chaftari AM, Jordan M, Hachem R, Al Hamal Z, Jiang Y, Yousif A, Garoge K, Deshmukh P, Raad I. A clinical practical approach to the surveillance definition of central line-associated bloodstream infection in cancer patients with mucosal barrier injury. Am J Infect Control. 2016 Aug 1;44(8):931-4. DOI: 10.1016/j.ajic.2016.03.011
337. Satwani P, Freedman JL, Chaudhury S, Jin Z, Levinson A, Foca MD, Krajewski J, Sahdev I, Talekar MK, Gardenswartz A, Silverman J, Hayes M, Dvorak CC. A Multicenter Study of Bacterial Blood Stream Infections in Pediatric Allogeneic Hematopoietic Cell Transplantation Recipients: The Role of Acute Gastrointestinal Graft-versus-Host Disease. Biol Blood Marrow Transplant. 2017 Apr;23(4):642-7. DOI: 10.1016/j.bbmt.2017.01.073
338. Allaway Z, Phillips RS, Thursky KA, Haeusler GM. Nonneutropenic fever in children with cancer: A scoping review of management and outcome. Pediatr Blood Cancer. 2019 Jun;66(6):e27634. DOI: 10.1002/pbc.27634
339. Infektionsschutzgesetz vom 20. Juli 2000 (BGBl. I S. 1045), das zuletzt durch Artikel 5 des Gesetzes vom 19. Juni 2020 (BGBl. I S. 1385) geändert worden ist.
340. Shaw BE, Boswell T, Byrne JL, Yates C, Russell NH. Clinical impact of MRSA in a stem cell transplant unit: analysis before, during and after an MRSA outbreak. Bone Marrow Transplant. 2007;39(10):623-9.
341. Miles-Jay A, Podczervinski S, Stednick ZJ, Pergam SA. Evaluation of routine pretransplantation screening for methicillin-resistant Staphylococcus aureus in hematopoietic cell transplant recipients. Am J Infect Control. 2015 Jan;43(1):89-91. DOI: 10.1016/j.ajic.2014.10.010
342. Liss BJ, Vehreschild JJ, Cornely OA, Hallek M, Fätkenheuer G, Wisplinghoff H, Seifert H, Vehreschild MJ. Intestinal colonisation and blood stream infections due to vancomycin-resistant enterococci (VRE) and extended-spectrum beta-lactamase-producing Enterobacteriaceae (ESBLE) in patients with haematological and oncological malignancies. Infection. 2012 Dec;40(6):613-9. DOI: 10.1007/s15010-012-0269-y
343. Ziakas PD, Pliakos EE, Zervou FN, Knoll BM, Rice LB, Mylonakis E. MRSA and VRE colonization in solid organ transplantation: a meta-analysis of published studies. Am J Transplant. 2014 Aug;14(8):1887-94. DOI: 10.1111/ajt.12784
344. Bert F, Larroque B, Dondero F, Durand F, Paugam-Burtz C, Belghiti J, Moreau R, Nicolas-Chanoine MH. Risk factors associated with preoperative fecal carriage of extended-spectrum β-lactamase-producing Enterobacteriaceae in liver transplant recipients. Transpl Infect Dis. 2014 Feb;16(1):84-9. DOI: 10.1111/tid.12169
345. Webb BJ, Healy R, Majers J, Burr Z, Gazdik M, Lopansri B, Hoda D, Petersen FB, Ford C. Prediction of Bloodstream Infection Due to Vancomycin-Resistant Enterococcus in Patients Undergoing Leukemia Induction or Hematopoietic Stem-Cell Transplantation. Clin Infect Dis. 2017 Jun 15;64(12):1753-9. DOI: 10.1093/cid/cix232
346. Averbuch D, Orasch C, Cordonnier C, Livermore DM, Mikulska M, Viscoli C, Gyssens IC, Kern WV, Klyasova G, Marchetti O, Engelhard D, Akova M; ECIL4, a joint venture of EBMT, EORTC, ICHS, ESGICH/ESCMID and ELN. European guidelines for empirical antibacterial therapy for febrile neutropenic patients in the era of growing resistance: summary of the 2011 4th European Conference on Infections in Leukemia. Haematologica. 2013 Dec;98(12):1826-35. DOI: 10.3324/haematol.2013.091025
347. Baker TM, Satlin MJ. The growing threat of multidrug-resistant Gram-negative infections in patients with hematologic malignancies. Leuk Lymphoma. 2016 Oct;57(10):2245-58. DOI: 10.1080/10428194.2016.1193859

348. Friedman ND, Carmeli Y, Walton AL, Schwaber MJ. Carbapenem-Resistant Enterobacteriaceae: A Strategic Roadmap for Infection Control. Infect Control Hosp Epidemiol. 2017 May;38(5):580-94. DOI: 10.1017/ice.2017.42
349. Holland T, Fowler VG Jr, Shelburne SA 3rd. Invasive gram-positive bacterial infection in cancer patients. Clin Infect Dis. 2014 Nov;59 Suppl 5:S331-4. DOI: 10.1093/cid/ciu598
350. Munoz-Price LS, Poirel L, Bonomo RA, Schwaber MJ, Daikos GL, Cormican M, Cornaglia G, Garau J, Gniadkowski M, Hayden MK, Kumarasamy K, Livermore DM, Maya JJ, Nordmann P, Patel JB, Paterson DL, Pitout J, Villegas MV, Wang H, Woodford N, Quinn JP. Clinical epidemiology of the global expansion of Klebsiella pneumoniae carbapenemases. Lancet Infect Dis. 2013 Sep;13(9):785-96. DOI: 10.1016/S1473-3099(13)70190-7
351. Trubiano JA, Worth LJ, Thursky KA, Slavin MA. The prevention and management of infections due to multidrug resistant organisms in haematology patients. Br J Clin Pharmacol. 2015 Feb;79(2):195-207. DOI: 10.1111/bcp.12310
352. Heidenreich D, Kreil S, Jawhar M, Müller N, Nolte F, Becker KP, Miethke T, Hofmann WK, Klein SA. Course of colonization by multidrug-resistant organisms after allogeneic hematopoietic cell transplantation. Ann Hematol. 2018 Dec;97(12):2501-8. DOI: 10.1007/s00277-018-3475-6
353. Heidenreich D, Kreil S, Nolte F, Hofmann WK, Miethke T, Klein SA. Multidrug-resistant organisms in allogeneic hematopoietic cell transplantation. Eur J Haematol. 2017 May;98(5):485-92. DOI: 10.1111/ejh.12859
354. Bartoletti M, Giannella M, Tedeschi S, Viale P. Multidrug-Resistant Bacterial Infections in Solid Organ Transplant Candidates and Recipients. Infect Dis Clin North Am. 2018 Sep;32(3):551-80. DOI: 10.1016/j.idc.2018.04.004
355. Rohde AM, Wiese-Posselt M, Zweigner J, Schwab F, Mischnik A, Seifert H, Gastmeier P, Kern WV; DZIF-ATHOS Study Group. High admission prevalence of fluoroquinolone resistance in third-generation cephalosporin-resistant Enterobacteriaceae in German university hospitals. J Antimicrob Chemother. 2018 Jun;73(6):1688-91. DOI: 10.1093/jac/dky040
356. Seo GH, Kim MJ, Seo S, Hwang B, Lee E, Yun Y, Choi M, Kim M, Kim JW, Kim ES, Kim HB, Song KH. Cancer-specific incidence rates of tuberculosis: A 5-year nationwide population-based study in a country with an intermediate tuberculosis burden. Medicine (Baltimore). 2016 Sep;95(38):e4919. DOI: 10.1097/MD.0000000000004919
357. Simonsen DF FD, Horsburgh CR. Increased risk of active tuberculosis after cancer diagnosis. J Infect Chemother. 2017;74:590-8. DOI: 10.1016/j.jinf.2017.03.012
358. Kommission für Krankenhaushygiene und Infektionsprävention (KRINKO). Aspekte der mikrobiologischen Diagnostik im Rahmen der Prävention von nosokomialen Infektionen. Epid Bull. 2013;(19):171-2.
359. Neumann S, Krause SW, Maschmeyer G, Schiel X, von Lilienfeld-Toal M; Infectious Diseases Working Party (AGIHO); German Society of Hematology and Oncology (DGHO). Primary prophylaxis of bacterial infections and Pneumocystis jirovecii pneumonia in patients with hematological malignancies and solid tumors : guidelines of the Infectious Diseases Working Party (AGIHO) of the German Society of Hematology and Oncology (DGHO). Ann Hematol. 2013 Apr;92(4):433-42. DOI: 10.1007/s00277-013-1698-0
360. Baden LR, Swaminathan S, Angarone M, Blouin G, Camins BC, Casper C, Cooper B, Dubberke ER, Engemann AM, Freifeld AG, Greene JN, Ito JI, Kaul DR, Lustberg ME, Montoya JG, Rolston K, Satyanarayana G, Segal B, Seo SK, Shoham S, Taplitz R, Topal J, Wilson JW, Hoffmann KG, Smith C. Prevention and Treatment of Cancer-Related Infections, Version 2.2016, NCCN Clinical Practice Guidelines in Oncology. J Natl Compr Canc Netw. 2016 Jul;14(7):882-913. DOI: 10.6004/jnccn.2016.0093
361. Tacconelli E, Sifakis F, Harbarth S, Schrijver R, van Mourik M, Voss A, Sharland M, Rajendran NB, Rodríguez-Baño J; EPI-Net COMBACTE-MAGNET Group. Surveillance for control of antimicrobial resistance. Lancet Infect Dis. 2018 Mar;18(3):e99-e106. DOI: 10.1016/S1473-3099(17)30485-1
362. Rangaraj G, Granwehr BP, Jiang Y, Hachem R, Raad I. Perils of quinolone exposure in cancer patients: breakthrough bacteremia with multidrug-resistant organisms. Cancer. 2010 Feb;116(4):967-73. DOI: 10.1002/cncr.24812
363. Mikulska M, Cordonnier C. Fluoroquinolone prophylaxis during neutropenia: what can we expect nowadays? Clin Microbiol Infect. 2018 Jul;24(7):678-9. DOI: 10.1016/j.cmi.2018.02.031
364. Verlinden A, Jansens H, Goossens H, van de Velde AL, Schroyens WA, Berneman ZN, Gadisseur AP. Clinical and microbiological impact of discontinuation of fluoroquinolone prophylaxis in patients with prolonged profound neutropenia. Eur J Haematol. 2014 Oct;93(4):302-8. DOI: 10.1111/ejh.12345
365. Haeusler GM, Slavin MA. Fluoroquinolone prophylaxis: worth the cost? Leuk Lymphoma. 2013 Apr;54(4):677-8. DOI: 10.3109/10428194.2012.736988
366. Saini L, Rostein C, Atenafu EG, Brandwein JM. Ambulatory consolidation chemotherapy for acute myeloid leukemia with antibacterial prophylaxis is associated with frequent bacteremia and the emergence of fluoroquinolone resistant E. Coli. BMC Infect Dis. 2013 Jun;13:284. DOI: 10.1186/1471-2334-13-284
367. Lehrnbecher T, Fisher BT, Phillips B, Alexander S, Ammann RA, Beauchemin M, Carlesse F, Castagnola E, Davis BL, Dupuis LL, Egan G, Groll AH, Haeusler GM, Santolaya M, Steinbach WJ, van de Wetering M, Wolf J, Cabral S, Robinson PD, Sung L. Guideline for Antibacterial Prophylaxis Administration in Pediatric Cancer and Hematopoietic Stem Cell Transplantation. Clin Infect Dis. 2020 Jun;71(1):226-36. DOI: 10.1093/cid/ciz1082
368. Egan G, Robinson PD, Martinez JPD, Alexander S, Ammann RA, Dupuis LL, Fisher BT, Lehrnbecher T, Phillips B, Cabral S, Tomlinson G, Sung L. Efficacy of antibiotic prophylaxis in patients with cancer and hematopoietic stem cell transplantation recipients: A systematic review of randomized trials. Cancer Med. 2019 Aug;8(10):4536-46. DOI: 10.1002/cam4.2395

369. Elishoov H, Or R, Strauss N, Engelhard D. Nosocomial colonization, septicemia, and Hickman/Broviac catheter-related infections in bone marrow transplant recipients. A 5-year prospective study. Medicine (Baltimore). 1998 Mar;77(2):83-101. DOI: 10.1097/00005792-199803000-00002
370. Cohen ML, Murphy MT, Counts GW, Buckner CD, Clift RA, Meyers JD. Prediction by surveillance cultures of bacteremia among neutropenic patients treated in a protective environment. J Infect Dis. 1983;147(5):789-93.
371. Daw MA, Munnelly P, McCann SR, Daly PA, Falkiner FR, Keane CT. Value of surveillance cultures in the management of neutropenic patients. Eur J Clin Microbiol Infect Dis. 1988 Dec;7(6):742-7. DOI: 10.1007/BF01975040
372. de Jong PJ, de Jong MD, Kuijper EJ, van der Lelie H. The value of surveillance cultures in neutropenic patients receiving selective intestinal decontamination. Scand J Infect Dis. 1993;25(1):107-13.
373. Feld R. The role of surveillance cultures in patients likely to develop chemotherapy-induced mucositis. Support Care Cancer. 1997 Sep;5(5):371-5. DOI: 10.1007/s005200050094
374. Baier C, Linderkamp C, Beilken A, Thol F, Heuser M, Ebadi E, Ganzenmueller T, Heim A, Bange FC. Influenza and respiratory syncytial virus screening for the detection of asymptomatically infected patients in hematology and oncology. GMS Hyg Infect Control. 2018 Sep 24;13:Doc08. DOI: 10.3205/dgkh000314
375. Hermann B, Lehners N, Brodhun M, Boden K, Hochhaus A, Kochanek M, Meckel K, Mayer K, Rachow T, Rieger C, Schalk E, Weber T, Schmeier-Jürchott A, Schlattmann P, Teschner D, von Lilienfeld-Toal M. Influenza virus infections in patients with malignancies -- characteristics and outcome of the season 2014/15. A survey conducted by the Infectious Diseases Working Party (AGIHO) of the German Society of Haematology and Medical Oncology (DGHO). Eur J Clin Microbiol Infect Dis. 2017 Mar;36(3):565-73. DOI: 10.1007/s10096-016-2833-3
376. French CE, McKenzie BC, Coope C, Rajanaidu S, Paranthaman K, Pebody R, Nguyen-Van-Tam JS; Noso-RSV Study GroupHiggins JP, Beck CR. Risk of nosocomial respiratory syncytial virus infection and effectiveness of control measures to prevent transmission events: a systematic review. Influenza Other Respir Viruses. 2016 Jul;10(4):268-90. DOI: 10.1111/irv.12379
377. RSV Outbreak Investigation Team. Contributing and Terminating Factors of a Large RSV Outbreak in an Adult Hematology and Transplant Unit. PLoS Curr. 2014 Sep 19;6:ecurrents.outbreaks.3bc85b2a508d205ecc4a5534ecb1f9be. DOI: 10.1371/currents.outbreaks.3bc85b2a508d205ecc4a5534ecb1f9be
378. Inkster T, Ferguson K, Edwardson A, Gunson R, Soutar R. Consecutive yearly outbreaks of respiratory syncytial virus in a haemato-oncology ward and efficacy of infection control measures. J Hosp Infect. 2017;96(4):353-9.
379. Jensen TO, Stelzer-Braid S, Willenborg C, Cheung C, Andresen D, Rawlinson W, Clezy K. Outbreak of respiratory syncytial virus (RSV) infection in immunocompromised adults on a hematology ward. J Med Virol. 2016 Oct;88(10):1827-31. DOI: 10.1002/jmv.24521
380. Gudiol C, Verdaguer R, Angeles Dominguez M, Fernandez-Sevilla A, Carratala J. Outbreak of Legionnaires' disease in immunosuppressed patients at a cancer centre: usefulness of universal urine antigen testing and early levofloxacin therapy. Clin Microbiol Infect. 2007;13(11):1125-8.
381. Deutsche Gesellschaft für Pädiatrische Infektiologie (DGPI). S2k Leitlinie „Antibiotic Stewardship – Konzeption und Umsetzung in der stationären Kinder- und Jugendmedizin“. AWMF-Registernummer 048/15. AWMF; 2018 [cited 2020 Nov 01]. Available from: https://www.awmf.org/uploads/tx_szleitlinien/048-015l_S2k_Antibiotic-Stewardship-ABS-Konzeption-Umsetzung-stationaere-Kinder-Jugendmedizin_2019-06.pdf
382. de With K, Wilke K, Kern WV, Strauß R, Kramme E, Friedrichs A, s Holzmann T, Geiss HK, Isner C, Fellhauer M, von Ameln-Mayerhofer A, Abele-Horn M, Häcker G, Walger P, Deja M, Vehreschild JJ, Kather A, Friese E, Porsche U, Janata O, Krause R, Wechsler-Fördös A. S3-Leitlinie. Strategien zur Sicherung rationaler Antibiotika-Anwendung im Krankenhaus. AWMF-Registernummer 092-001 – update 2018 (Stand: 31.01.2019). AWMF; 2019 [cited 2020 Nov 01]. Available from: https://www.awmf.org/uploads/tx_szleitlinien/092-001l_S3_Strategien-zur-Sicherung-rationaler-Antibiotika-Anwendung-im-Krankenhaus_2020-02.pdf
383. Dik JH, Poelman R, Friedrich AW, Niesters HGM, Rossen JWA, Sinha B. Integrated Stewardship Model Comprising Antimicrobial, Infection Prevention, and Diagnostic Stewardship (AID Stewardship). J Clin Microbiol. 2017;55(11):3306-7. DOI: 10.1128/JCM.01283-17
384. Dik JW, Poelman R, Friedrich AW, Panday PN, Lo-Ten-Foe JR, van Assen S, van Gemert-Pijnen JE, Niesters HG, Hendrix R, Sinha B. An integrated stewardship model: antimicrobial, infection prevention and diagnostic (AID). Future Microbiol. 2016;11(1):93-102. DOI: 10.2217/fmb.15.99
385. Manning ML, Septimus EJ, Ashley ESD, Cosgrove SE, Fakih MG, Schweon SJ, Myers FE, Moody JA. Antimicrobial Stewardship and Infection Prevention-Leveraging the Synergy: A Position Paper Update. Infect Control Hosp Epidemiol. 2018 Apr;39(4):467-72. DOI: 10.1017/ice.2018.33
386. Mielke M. Die Rolle der Infektionsprävention bei der Eindämmung der Antibiotikaresistenzentwicklung. Jede vermiedene Infektion trägt zur Reduktion des Antibiotikaeinsatzes bei. Bundesgesundheitsbl. 2018;61(5):553-61.
387. Septimus EJ. Antimicrobial Resistance: An Antimicrobial/Diagnostic Stewardship and Infection Prevention Approach. Med Clin North Am. 2018 Sep;102(5):819-29. DOI: 10.1016/j.mcna.2018.04.005
388. Schelenz S, Nwaka D, Hunter PR. Longitudinal surveillance of bacteraemia in haematology and oncology patients at a UK cancer centre and the impact of ciprofloxacin use on antimicrobial resistance. J Antimicrob Chemother. 2013;68(6):1431-8.
389. Iacob S, Iacob DG. Infectious Threats, the Intestinal Barrier, and Its Trojan Horse: Dysbiosis. Front Microbiol. 2019;10:1676. DOI: 10.3389/fmicb.2019.01676

390. Araoka H, Fujii T, Izutsu K, Kimura M, Nishida A, Ishiwata K, Nakano N, Tsuji M, Yamamoto H, Asano-Mori Y, Uchida N, Wake A, Taniguchi S, Yoneyama A. Rapidly progressive fatal hemorrhagic pneumonia caused by Stenotrophomonas maltophilia in hematologic malignancy. Transpl Infect Dis. 2012 Aug;14(4):355-63. DOI: 10.1111/j.1399-3062.2011.00710.x
391. Arnan M, Gudiol C, Calatayud L, Liñares J, Dominguez MÁ, Batlle M, Ribera JM, Carratalà J, Gudiol F. Risk factors for, and clinical relevance of, faecal extended-spectrum β-lactamase producing Escherichia coli (ESBL-EC) carriage in neutropenic patients with haematological malignancies. Eur J Clin Microbiol Infect Dis. 2011 Mar;30(3):355-60. DOI: 10.1007/s10096-010-1093-x
392. Averbuch D, Avaky C, Harit M, Stepensky P, Fried I, Ben-Ami T, Temper V, Peled Y, Troen H, Masarwa R, Abu Ahmad W, Weintraub M, Revel-Vilk S, Engelhard D. Non-fermentative Gram-negative rods bacteremia in children with cancer: a 14-year single-center experience. Infection. 2017 Jun;45(3):327-34. DOI: 10.1007/s15010-017-0988-1
393. Averbuch D, Cordonnier C, Livermore DM, Mikulska M, Orasch C, Viscoli C, Gyssens IC, Kern WV, Klyasova G, Marchetti O, Engelhard D, Akova M; ECIL4, a joint venture of EBMT, EORTC, ICHS, ESGICH/ESCMID and ELN. Targeted therapy against multi-resistant bacteria in leukemic and hematopoietic stem cell transplant recipients: guidelines of the 4th European Conference on Infections in Leukemia (ECIL-4, 2011). Haematologica. 2013 Dec;98(12):1836-47. DOI: 10.3324/haematol.2013.091330
394. Bhusal Y, Mihu CN, Tarrand JJ, Rolston KV. Incidence of fluoroquinolone-resistant and extended-spectrum beta-lactamase-producing Escherichia coli at a comprehensive cancer center in the United States. Chemotherapy. 2011;57(4):335-8. DOI: 10.1159/000329661
395. Brooke JS. Stenotrophomonas maltophilia: an emerging global opportunistic pathogen. Clin Microbiol Rev. 2012 Jan;25(1):2-41. DOI: 10.1128/CMR.00019-11
396. Carattoli A, Fortini D, Galetti R, Garcia-Fernandez A, Nardi G, Orazi D, Capone A, Majolino I, Proia A, Mariani B, Parisi G, Morrone A, Petrosillo N. Isolation of NDM-1-producing Pseudomonas aeruginosa sequence type ST235 from a stem cell transplant patient in Italy, May 2013. Euro Surveill. 2013 Nov;18(46). DOI: 10.2807/1560-7917.es2013.18.46.20633
397. Ciofi Degli Atti M, Bernaschi P, Carletti M, Luzzi I, García-Fernández A, Bertaina A, Sisto A, Locatelli F, Raponi M. An outbreak of extremely drug-resistant Pseudomonas aeruginosa in a tertiary care pediatric hospital in Italy. BMC Infect Dis. 2014 Sep 10;14:494. DOI: 10.1186/1471-2334-14-494
398. Fukuta Y, Muder RR, Agha ME, Clarke LG, Wagener MM, Hensler AM, Doi Y. Risk factors for acquisition of multidrug-resistant Acinetobacter baumannii among cancer patients. Am J Infect Control. 2013 Dec;41(12):1249-52. DOI: 10.1016/j.ajic.2013.04.003
399. Gao W, Howden BP, Stinear TP. Evolution of virulence in Enterococcus faecium, a hospital-adapted opportunistic pathogen. Curr Opin Microbiol. 2018 Feb;41:76-82. DOI: 10.1016/j.mib.2017.11.030
400. Gudiol C, Bodro M, Simonetti A, Tubau F, González-Barca E, Cisnal M, Domingo-Domenech E, Jiménez L, Carratalà J. Changing aetiology, clinical features, antimicrobial resistance, and outcomes of bloodstream infection in neutropenic cancer patients. Clin Microbiol Infect. 2013 May;19(5):474-9. DOI: 10.1111/j.1469-0691.2012.03879.x
401. Gudiol C, Calatayud L, Garcia-Vidal C, Lora-Tamayo J, Cisnal M, Duarte R, Arnan M, Marin M, Carratalà J, Gudiol F. Bacteraemia due to extended-spectrum beta-lactamase-producing Escherichia coli (ESBL-EC) in cancer patients: clinical features, risk factors, molecular epidemiology and outcome. J Antimicrob Chemother. 2010 Feb;65(2):333-41. DOI: 10.1093/jac/dkp411
402. Haeusler GM, Mechinaud F, Daley AJ, Starr M, Shann F, Connell TG, Bryant PA, Donath S, Curtis N. Antibiotic-resistant Gram-negative bacteremia in pediatric oncology patients--risk factors and outcomes. Pediatr Infect Dis J. 2013 Jul;32(7):723-6. DOI: 10.1097/INF.0b013e31828aebc8
403. Kim SB, Min YH, Cheong JW, Kim JS, Kim SJ, Ku NS, Jeong SJ, Han SH, Choi JY, Song YG, Kim JM. Incidence and risk factors for carbapenem- and multidrug-resistant Acinetobacter baumannii bacteremia in hematopoietic stem cell transplantation recipients. Scand J Infect Dis. 2014 Feb;46(2):81-8. DOI: 10.3109/00365548.2013.857042
404. Perez F, Adachi J, Bonomo RA. Antibiotic-resistant gram-negative bacterial infections in patients with cancer. Clin Infect Dis. 2014 Nov;59 Suppl 5:S335-9. DOI: 10.1093/cid/ciu612
405. Snitkin ES, Zelazny AM, Thomas PJ, Stock F; NISC Comparative Sequencing Program GroupHenderson DK, Palmore TN, Segre JA. Tracking a hospital outbreak of carbapenem-resistant Klebsiella pneumoniae with whole-genome sequencing. Sci Transl Med. 2012 Aug;4(148):148ra116. DOI: 10.1126/scitranslmed.3004129
406. Tada K, Kurosawa S, Hiramoto N, Okinaka K, Ueno N, Asakura Y, Kim SW, Yamashita T, Mori SI, Heike Y, Maeshima AM, Tanosaki R, Tobinai K, Fukuda T. Stenotrophomonas maltophilia infection in hematopoietic SCT recipients: high mortality due to pulmonary hemorrhage. Bone Marrow Transplant. 2013 Jan;48(1):74-9. DOI: 10.1038/bmt.2012.87
407. Tschudin-Sutter S, Lucet JC, Mutters NT, Tacconelli E, Zahar JR, Harbarth S. Contact Precautions for Preventing Nosocomial Transmission of Extended-Spectrum β Lactamase-Producing Escherichia coli: A Point/Counterpoint Review. Clin Infect Dis. 2017 Jul;65(2):342-7. DOI: 10.1093/cid/cix258
408. von Lilienfeld-Toal M, Maschmeyer G. Challenges in Infectious Diseases for Haematologists. Oncol Res Treat. 2018;41(6):406-10. DOI: 10.1159/000487439
409. Yeo CL, Chan DS, Earnest A, Wu TS, Yeoh SF, Lim R, Jureen R, Fisher D, Hsu LY. Prospective audit and feedback on antibiotic prescription in an adult hematology-oncology unit in Singapore. Eur J Clin Microbiol Infect Dis. 2012 Apr;31(4):583-90. DOI: 10.1007/s10096-011-1351-6

410. Yeo CL, Wu JE, Chung GW, Chan DS, Fisher D, Hsu LY. Specialist trainees on rotation cannot replace dedicated consultant clinicians for antimicrobial stewardship of specialty disciplines. Antimicrob Resist Infect Control. 2012 Nov;1(1):36. DOI: 10.1186/2047-2994-1-36
411. Trecarichi EM, Tumbarello M, Spanu T, Caira M, Fianchi L, Chiusolo P, Fadda G, Leone G, Cauda R, Pagano L. Incidence and clinical impact of extended-spectrum-beta-lactamase (ESBL) production and fluoroquinolone resistance in bloodstream infections caused by Escherichia coli in patients with hematological malignancies. J Infect. 2009 Apr;58(4):299-307. DOI: 10.1016/j.jinf.2009.02.002
412. Aguiar EB, Maciel LC, Halpern M, de Lemos AS, Ferreira AL, Basto ST, Gonçalves RT, de Gouvêa EF, Santoro-Lopes G. Outcome of bacteremia caused by extended-spectrum β-lactamase-producing Enterobacteriaceae after solid organ transplantation. Transplant Proc. 2014 Jul-Aug;46(6):1753-6. DOI: 10.1016/j.transproceed.2014.05.003
413. Mikulska M, Del Bono V, Bruzzi P, Raiola AM, Gualandi F, Van Lint MT, Bacigalupo A, Viscoli C. Mortality after bloodstream infections in allogeneic haematopoietic stem cell transplant (HSCT) recipients. Infection. 2012 Jun;40(3):271-8. DOI: 10.1007/s15010-011-0229-y
414. Tang Y, Wu X, Cheng Q, Li X. Inappropriate initial antimicrobial therapy for hematological malignancies patients with Gram-negative bloodstream infections. Infection. 2020 Feb;48(1):109-16. DOI: 10.1007/s15010-019-01370-x
415. Shono Y, Docampo MD, Peled JU, Perobelli SM, Velardi E, Tsai JJ, Slingerland AE, Smith OM, Young LF, Gupta J, Lieberman SR, Jay HV, Ahr KF, Porosnicu Rodriguez KA, Xu K, Calarfiore M, Poeck H, Caballero S, Devlin SM, Rapaport F, Dudakov JA, Hanash AM, Gyurkocza B, Murphy GF, Gomes C, Liu C, Moss EL, Falconer SB, Bhatt AS, Taur Y, Pamer EG, van den Brink MRM, Jenq RR. Increased GVHD-related mortality with broad-spectrum antibiotic use after allogeneic hematopoietic stem cell transplantation in human patients and mice. Sci Transl Med. 2016 May;8(339):339ra71. DOI: 10.1126/scitranslmed.aaf2311
416. Zimmermann P, Curtis N. The effect of antibiotics on the composition of the intestinal microbiota – a systematic review. J Infect. 2019 Dec;79(6):471-89. DOI: 10.1016/j.jinf.2019.10.008
417. Palacios-Baena ZR, Gutiérrez-Gutiérrez B, Calbo E, Almirante B, Viale P, Oliver A, Pintado V, Gasch O, Martínez-Martínez L, Pitout J, Akova M, Peña C, Molina Gil-Bermejo J, Hernández A, Venditti M, Prim N, Bou G, Tacconelli E, Tumbarello M, Hamprecht A, Giamarellou H, Almela M, Pérez F, Schwaber MJ, Bermejo J, Lowman W, Hsueh PR, Paño-Pardo JR, Torre-Cisneros J, Souli M, Bonomo RA, Carmeli Y, Paterson DL, Pascual Á, Rodríguez-Baño J; Spanish Network for Research in Infectious Diseases (REIPI)/European Study Group of Bloodstream Infections and Sepsis (ESGBIS)/INCREMENT Group. Empiric Therapy With Carbapenem-Sparing Regimens for Bloodstream Infections due to Extended-Spectrum β-Lactamase-Producing Enterobacteriaceae: Results From the INCREMENT Cohort. Clin Infect Dis. 2017 Oct 30;65(10):1615-23. DOI: 10.1093/cid/cix606
418. Short E, Esterly J, Postelnick M, Ong J, McLaughlin M. Disposition of linezolid or daptomycin in Enterococcal bloodstream infections according to vancomycin resistant Enterococcus colonization. Antimicrob Resist Infect Control. 2014;3(1):37. DOI: 10.1186/2047-2994-3-37
419. Kamboj M, Cohen N, Huang YT, Kerpelev M, Jakubowski A, Sepkowitz KA, Papanicolaou GA, Seo SK. Impact of Empiric Treatment for Vancomycin-Resistant Enterococcus in Colonized Patients Early after Allogeneic Hematopoietic Stem Cell Transplantation. Biol Blood Marrow Transplant. 2019 Mar;25(3):594-8. DOI: 10.1016/j.bbmt.2018.11.008
420. Cervantes J. Use your antibiotics wisely. Consequences to the intestinal microbiome. FEMS Microbiol Lett. 2016;363(10):fnw081.
421. Martinez-Nadal G, Puerta-Alcalde P, Gudiol C, Cardozo C, Albasanz-Puig A, Marco F, Laporte-Amargós J, Moreno-García E, Domingo-Doménech E, Chumbita M, Martínez JA, Soriano A, Carratalà J, Garcia-Vidal C. Inappropriate Empirical Antibiotic Treatment in High-risk Neutropenic Patients With Bacteremia in the Era of Multidrug Resistance. Clin Infect Dis. 2020 Mar;70(6):1068-74. DOI: 10.1093/cid/ciz319
422. Trubiano JA, Beekmann SE, Worth LJ, Polgreen PM, Thursky KA, Slavin MA, Grayson ML, Phillips EJ. Improving Antimicrobial Stewardship by Antibiotic Allergy Delabeling: Evaluation of Knowledge, Attitude, and Practices Throughout the Emerging Infections Network. Open Forum Infect Dis. 2016 Sep;3(3):ofw153. DOI: 10.1093/ofid/ofw153
423. Trubiano JA, Chen C, Cheng AC, Grayson ML, Slavin MA, Thursky KA; National Antimicrobial Prescribing Survey (NAPS). Antimicrobial allergy 'labels' drive inappropriate antimicrobial prescribing: lessons for stewardship. J Antimicrob Chemother. 2016 Jun;71(6):1715-22. DOI: 10.1093/jac/dkw008
424. Trubiano JA, Slavin MA, Thursky KA, Grayson ML, Phillips EJ. Beta-Lactam and Sulfonamide Allergy Testing Should Be a Standard of Care in Immunocompromised Hosts. J Allergy Clin Immunol Pract. 2019 Sep-Oct;7(7):2151-3. DOI: 10.1016/j.jaip.2019.05.051
425. Stover KR, Barber KE, Wagner JL. Allergic Reactions and Cross-Reactivity Potential with Beta-Lactamase Inhibitors. Pharmacy (Basel). 2019 Jun 28;7(3):77. DOI: 10.3390/pharmacy7030077
426. Stover KR, Bland CM, Gallagher JC; Society of Infectious Diseases Pharmacists. The Point of Antimicrobial Susceptibility Testing Is to Inform Antimicrobial Prescribing. Clin Infect Dis. 2017 Jan;64(1):103-4. DOI: 10.1093/cid/ciw686
427. Stone CA Jr, Trubiano J, Coleman DT, Rukasin CRF, Phillips EJ. The challenge of de-labeling penicillin allergy. Allergy. 2020 Feb;75(2):273-88. DOI: 10.1111/all.13848
428. Huang KG, Cluzet V, Hamilton K, Fadugba O. The Impact of Reported Beta-Lactam Allergy in Hospitalized Patients With Hematologic Malignancies Requiring Antibiotics. Clin Infect Dis. 2018 Jun;67(1):27-33. DOI: 10.1093/cid/ciy037

429. Agrawal S, Barnes R, Brüggemann RJ, Rautemaa-Richardson R, Warris A. The role of the multidisciplinary team in antifungal stewardship. J Antimicrob Chemother. 2016 Nov;71(suppl 2):ii37-ii42. DOI: 10.1093/jac/dkw395
430. Aguado JM, Silva JT, Bouza E. Conclusion and future perspectives on antifungal stewardship. J Antimicrob Chemother. 2016 Nov;71(suppl 2):ii43-4. DOI: 10.1093/jac/dkw396
431. Farmakiotis D, Kontoyiannis DP. Epidemiology of antifungal resistance in human pathogenic yeasts: current viewpoint and practical recommendations for management. Int J Antimicrob Agents. 2017 Sep;50(3):318-24. DOI: 10.1016/j.ijantimicag.2017.05.019
432. Hamdy RF, Zaoutis TE, Seo SK. Antifungal stewardship considerations for adults and pediatrics. Virulence. 2017 Aug;8(6):658-72. DOI: 10.1080/21505594.2016.1226721
433. Lachenmayr SJ, Berking S, Horns H, Strobach D, Ostermann H, Berger K. Antifungal treatment in haematological and oncological patients: Need for quality assessment in routine care. Mycoses. 2018 Jul;61(7):464-71. DOI: 10.1111/myc.12768
434. Mellinghoff SC, Hartmann P, Cornely FB, Knauth L, Köhler F, Köhler P, Krause C, Kronenberg C, Kranz SL, Menon V, Müller H, Naendrup JH, Pützfeld S, Ronge A, Rutz J, Seidel D, Wisplinghoff H, Cornely OA. Analyzing candidemia guideline adherence identifies opportunities for antifungal stewardship. Eur J Clin Microbiol Infect Dis. 2018 Aug;37(8):1563-71. DOI: 10.1007/s10096-018-3285-8
435. Micallef C, Aliyu SH, Santos R, Brown NM, Rosembert D, Enoch DA. Introduction of an antifungal stewardship programme targeting high-cost antifungals at a tertiary hospital in Cambridge, England. J Antimicrob Chemother. 2015;70(6):1908-11.
436. Micallef C, Ashiru-Oredope D, Hansraj S, Denning DW, Agrawal SG, Manuel RJ, Schelenz S, Guy R, Muller-Pebody B, Patel R, Howard P, Hopkins S, Johnson E, Enoch DA. An investigation of antifungal stewardship programmes in England. J Med Microbiol. 2017 Nov;66(11):1581-9. DOI: 10.1099/jmm.0.000612
437. Muñoz P, Bouza E; COMIC (Collaboration Group on Mycosis) study group. The current treatment landscape: the need for antifungal stewardship programmes. J Antimicrob Chemother. 2016 11;71(suppl 2):ii5-ii12. DOI: 10.1093/jac/dkw391
438. Ruhnke M. Antifungal stewardship in invasive Candida infections. Clin Microbiol Infect. 2014 Jun;20 Suppl 6:11-8. DOI: 10.1111/1469-0691.12622
439. Schwartz IS, Patterson TF. The Emerging Threat of Antifungal Resistance in Transplant Infectious Diseases. Curr Infect Dis Rep. 2018 Feb;20(3):2. DOI: 10.1007/s11908-018-0608-y
440. Valerio M, Muñoz P, Rodríguez-González C, Sanjurjo M, Guinea J, Bouza E; COMIC study group (Collaborative group on Mycosis). Training should be the first step toward an antifungal stewardship program. Enferm Infecc Microbiol Clin. 2015 Apr;33(4):221-7. DOI: 10.1016/j.eimc.2014.04.016
441. Valerio M, Muñoz P, Rodríguez CG, Caliz B, Padilla B, Fernández-Cruz A, Sánchez-Somolinos M, Gijón P, Peral J, Gayoso J, Frias I, Salcedo M, Sanjurjo M, Bouza E; COMIC Study Group Collaborative Group on Mycosis. Antifungal stewardship in a tertiary-care institution: a bedside intervention. Clin Microbiol Infect. 2015 May;21(5):492.e1-9. DOI: 10.1016/j.cmi.2015.01.013
442. Valerio M, Rodriguez-Gonzalez CG, Muñoz P, Caliz B, Sanjurjo M, Bouza E; COMIC Study Group (Collaborative Group on Mycoses). Evaluation of antifungal use in a tertiary care institution: antifungal stewardship urgently needed. J Antimicrob Chemother. 2014 Jul;69(7):1993-9. DOI: 10.1093/jac/dku053
443. Valerio M, Vena A, Bouza E, Reiter N, Viale P, Hochreiter M, Giannella M, Muñoz P; COMIC study group (Collaborative group on Mycosis). How much European prescribing physicians know about invasive fungal infections management? BMC Infect Dis. 2015 Feb;15:80. DOI: 10.1186/s12879-015-0809-z
444. Wattal C, Chakrabarti A, Oberoi JK, Donnelly JP, Barnes RA, Sherwal BL, Goel N, Saxena S, Varghese GM, Soman R, Loomba P, Tarai B, Singhal S, Mehta N, Ramasubramanian V, Choudhary D, Mehta Y, Ghosh S, Muralidhar S, Kaur R. Issues in antifungal stewardship: an opportunity that should not be lost. J Antimicrob Chemother. 2017 Apr 1;72(4):969-74. DOI: 10.1093/jac/dkw506
445. Lachenmayr SJ, Strobach D, Berking S, Horns H, Berger K, Ostermann H. Improving quality of antifungal use through antifungal stewardship interventions. Infection. 2019 Aug;47(4):603-10. DOI: 10.1007/s15010-019-01288-4
446. Seo SK, Lo K, Abbo LM. Current State of Antimicrobial Stewardship at Solid Organ and Hematopoietic Cell Transplant Centers in the United States. Infect Control Hosp Epidemiol. 2016 Oct;37(10):1195-200. DOI: 10.1017/ice.2016.149
447. Abbo LM, Ariza-Heredia EJ. Antimicrobial stewardship in immunocompromised hosts. Infect Dis Clin North Am. 2014 Jun;28(2):263-79. DOI: 10.1016/j.idc.2014.01.008
448. Cordonnier C, Pautas C, Maury S, Vekhoff A, Farhat H, Suarez F, Dhédin N, Isnard F, Ades L, Kuhnowski F, Foulet F, Kuentz M, Maison P, Bretagne S, Schwarzinger M. Empirical versus preemptive antifungal therapy for high-risk, febrile, neutropenic patients: a randomized, controlled trial. Clin Infect Dis. 2009 Apr;48(8):1042-51. DOI: 10.1086/597395
449. Cordonnier C, Robin C, Alanio A, Bretagne S. Antifungal pre-emptive strategy for high-risk neutropenic patients: why the story is still ongoing. Clin Microbiol Infect. 2014 Jun;20 Suppl 6:27-35. DOI: 10.1111/1469-0691.12428
450. Dumford DM 3rd, Skalweit M. Antibiotic-Resistant Infections and Treatment Challenges in the Immunocompromised Host. Infect Dis Clin North Am. 2016 Jun;30(2):465-89. DOI: 10.1016/j.idc.2016.02.008
451. Gyssens IC, Kern WV, Livermore DM; ECIL-4, a joint venture of EBMT, EORTC, ICHS and ESGICH of ESCMID. The role of antibiotic stewardship in limiting antibacterial resistance among hematology patients. Haematologica. 2013 Dec;98(12):1821-5. DOI: 10.3324/haematol.2013.091769

452. Hamandi B, Husain S, Humar A, Papadimitropoulos EA. Impact of infectious disease consultation on the clinical and economic outcomes of solid organ transplant recipients admitted for infectious complications. Clin Infect Dis. 2014 Oct;59(8):1074-82. DOI: 10.1093/cid/ciu522
453. la Martire G, Robin C, Oubaya N, Lepeule R, Beckerich F, Leclerc M, Barhoumi W, Toma A, Pautas C, Maury S, Akrout W, Cordonnier-Jourdin C, Fihman V, Venditti M, Cordonnier C. De-escalation and discontinuation strategies in high-risk neutropenic patients: an interrupted time series analyses of antimicrobial consumption and impact on outcome. Eur J Clin Microbiol Infect Dis. 2018 Oct;37(10):1931-40. DOI: 10.1007/s10096-018-3328-1
454. Lortholary O, Lefort A, Tod M, Chomat AM, Darras-Joly C, Cordonnier C; Club de Reflexion sur les Infections en Onco-Hématologie. Pharmacodynamics and pharmacokinetics of antibacterial drugs in the management of febrile neutropenia. Lancet Infect Dis. 2008 Oct;8(10):612-20. DOI: 10.1016/S1473-3099(08)70228-7
455. Mokart D, Slehofer G, Lambert J, Sannini A, Chow-Chine L, Brun JP, Berger P, Duran S, Faucher M, Blache JL, Saillard C, Vey N, Leone M. De-escalation of antimicrobial treatment in neutropenic patients with severe sepsis: results from an observational study. Intensive Care Med. 2014 Jan;40(1):41-9. DOI: 10.1007/s00134-013-3148-9
456. Paskovaty A, Pastores SM, Gedrimaite Z, Kostelecky N, Riedel ER, Seo SK. Antimicrobial de-escalation in septic cancer patients: is it safe to back down? Intensive Care Med. 2015 Nov;41(11):2022-3. DOI: 10.1007/s00134-015-4016-6
457. Reinecke J, Lowas S, Snowden J, Neemann K. Blood Stream Infections and Antibiotic Utilization in Pediatric Leukemia Patients With Febrile Neutropenia. J Pediatr Hematol Oncol. 2019 May;41(4):251-5. DOI: 10.1097/MPH.0000000000001279
458. Rosa R, Simkins J, Camargo JF, Martinez O, Abbo LM. Solid organ transplant antibiograms: an opportunity for antimicrobial stewardship. Diagn Microbiol Infect Dis. 2016 Dec;86(4):460-3. DOI: 10.1016/j.diagmicrobio.2016.08.018
459. Rosa RG, Dos Santos RP, Goldani LZ. Mortality related to coagulase-negative staphylococcal bacteremia in febrile neutropenia: A cohort study. Can J Infect Dis Med Microbiol. 2014;25(1):e14-7. DOI: 10.1155/2014/702621
460. Rosa RG, Goldani LZ. Cohort study of the impact of time to antibiotic administration on mortality in patients with febrile neutropenia. Antimicrob Agents Chemother. 2014 Jul;58(7):3799-803. DOI: 10.1128/AAC.02561-14
461. Rosa RG, Goldani LZ, dos Santos RP. Association between adherence to an antimicrobial stewardship program and mortality among hospitalised cancer patients with febrile neutropaenia: a prospective cohort study. BMC Infect Dis. 2014;14:286. DOI: 10.1186/1471-2334-14-286
462. Tverdek FP, Rolston KV, Chemaly RF. Antimicrobial stewardship in patients with cancer. Pharmacotherapy. 2012 Aug;32(8):722-34. DOI: 10.1002/j.1875-9114.2012.01162.x
463. Vicente M, Al-Nahedh M, Parsad S, Knoebel RW, Pisano J, Pettit NN. Impact of a clinical pathway on appropriate empiric vancomycin use in cancer patients with febrile neutropenia. J Oncol Pharm Pract. 2017 Dec;23(8):575-81. DOI: 10.1177/1078155216668672
464. Wattier RL, Levy ER, Sabnis AJ, Dvorak CC, Auerbach AD. Reducing Second Gram-Negative Antibiotic Therapy on Pediatric Oncology and Hematopoietic Stem Cell Transplantation Services. Infect Control Hosp Epidemiol. 2017 Sep;38(9):1039-47. DOI: 10.1017/ice.2017.118
465. Zhu LL, Zhou Q. Optimal infusion rate in antimicrobial therapy explosion of evidence in the last five years. Infect Drug Resist. 2018;11:1105-17. DOI: 10.2147/IDR.S167616
466. Robilotti E, Holubar M, Seo SK, Deresinski S. Feasibility and applicability of antimicrobial stewardship in immunocompromised patients. Curr Opin Infect Dis. 2017 Aug;30(4):346-53. DOI: 10.1097/QCO.0000000000000380
467. Puerta-Alcalde P, Cardozo C, Suárez-Lledó M, Rodríguez-Núñez O, Morata L, Fehér C, Marco F, Del Río A, Martínez JA, Mensa J, Rovira M, Esteve J, Soriano A, Garcia-Vidal C. Current time-to-positivity of blood cultures in febrile neutropenia: a tool to be used in stewardship de-escalation strategies. Clin Microbiol Infect. 2019 Apr;25(4):447-53. DOI: 10.1016/j.cmi.2018.07.026
468. Abele-Horn M, de With K, Seifert J, Eckmanns T, Schmidt N, Fellhauer M, Häcker G, Kern W, Liese J, Walger P. Strukturelle und personelle Voraussetzungen für die Sicherung einer rationalen Antiinfektivaverordnung in Krankenhäusern. Bundesgesundheitsbl. 2020;63(6):749-60.
469. Sax H, Clack L, Touveneau S, Jantarada Fda L, Pittet D, Zingg W; PROHIBIT study group. Implementation of infection control best practice in intensive care units throughout Europe: a mixed-method evaluation study. Implement Sci. 2013 Feb;8:24. DOI: 10.1186/1748-5908-8-24
470. Storr J, Twyman A, Zingg W, Damani N, Kilpatrick C, Reilly J, Price L, Egger M, Grayson ML, Kelley E, Allegranzi B; WHO Guidelines Development Group. Core components for effective infection prevention and control programmes: new WHO evidence-based recommendations. Antimicrob Resist Infect Control. 2017;6:6. DOI: 10.1186/s13756-016-0149-9
471. Goff DA, Kullar R, Bauer KA, File TM Jr. Eight Habits of Highly Effective Antimicrobial Stewardship Programs to Meet the Joint Commission Standards for Hospitals. Clin Infect Dis. 2017 Apr;64(8):1134-9. DOI: 10.1093/cid/cix065
472. Kern WV, Fellhauer M, Hug M, Hoppe-Tichy T, Först G, Steib-Bauert M, de With K. Antibiotika-Anwendung 2012/13 in 109 deutschen Akutkrankenhäusern [Recent antibiotic use in German acute care hospitals – from benchmarking to improved prescribing and quality care]. Dtsch Med Wochenschr. 2015 Nov;140(23):e237-46. DOI: 10.1055/s-0041-105938

473. Thern J, de With K, Strauss R, Steib-Bauert M, Weber N, Kern WV. Selection of hospital antimicrobial prescribing quality indicators: a consensus among German antibiotic stewardship (ABS) networkers. Infection. 2014 Apr;42(2):351-62. DOI: 10.1007/s15010-013-0559-z
474. Davies HD; Committee on Infectious Diseases. Infectious Complications With the Use of Biologic Response Modifiers in Infants and Children. Pediatrics. 2016 Aug;138(2):e20161209. doi: 10.1542/peds.2016-1209
475. Reinwald M, Silva JT, Mueller NJ, Fortún J, Garzoni C, de Fijter JW, Fernández-Ruiz M, Grossi P, Aguado JM. ESCMID Study Group for Infections in Compromised Hosts (ESGICH) Consensus Document on the safety of targeted and biological therapies: an infectious diseases perspective (Intracellular signaling pathways: tyrosine kinase and mTOR inhibitors). Clin Microbiol Infect. 2018 Jun;24(Suppl 2):S53-S70. DOI: 10.1016/j.cmi.2018.02.009
476. Baddley JW, Cantini F, Goletti D, Gómez-Reino JJ, Mylonakis E, San-Juan R, Fernández-Ruiz M, Torre-Cisneros J. ESCMID Study Group for Infections in Compromised Hosts (ESGICH) Consensus Document on the safety of targeted and biological therapies: an infectious diseases perspective (Soluble immune effector molecules [I]: anti-tumor necrosis factor-α agents). Clin Microbiol Infect. 2018 Jun;24(Suppl 2):S10-S20. DOI: 10.1016/j.cmi.2017.12.025
477. Drgona L, Gudiol C, Lanini S, Salzberger B, Ippolito G, Mikulska M. ESCMID Study Group for Infections in Compromised Hosts (ESGICH) Consensus Document on the safety of targeted and biological therapies: an infectious diseases perspective (Agents targeting lymphoid or myeloid cells surface antigens [II]: CD22, CD30, CD33, CD38, CD40, SLAMF-7 and CCR4). Clin Microbiol Infect. 2018;24(Suppl 2):S83-94.
478. Mikulska M, Lanini S, Gudiol C, Drgona L, Ippolito G, Fernández-Ruiz M, Salzberger B. ESCMID Study Group for Infections in Compromised Hosts (ESGICH) Consensus Document on the safety of targeted and biological therapies: an infectious diseases perspective (Agents targeting lymphoid cells surface antigens [I]: CD19, CD20 and CD52). Clin Microbiol Infect. 2018 Jun;24(Suppl 2):S71-S82. DOI: 10.1016/j.cmi.2018.02.003
479. Ioannou P, Vamvoukaki R, Samonis G. Rhodotorula species infections in humans: A systematic review. Mycoses. 2019 Feb;62(2):90-100. DOI: 10.1111/myc.12856
480. Potenza L, Chitasombat MN, Klimko N, Bettelli F, Dragonetti G, Del Principe MI, Nucci M, Busca A, Fracchiolla N, Sciumè M, Spolzino A, Delia M, Mancini V, Nadali GP, Dargenio M, Shadrivova O, Bacchelli F, Aversa F, Sanguinetti M, Luppi M, Kontoyiannis DP, Pagano L. Rhodotorula infection in haematological patient: Risk factors and outcome. Mycoses. 2019 Mar;62(3):223-9. DOI: 10.1111/myc.12875
481. Fabiani S, Fortunato S, Petrini M, Bruschi F. Allogeneic hematopoietic stem cell transplant recipients and parasitic diseases: A review of the literature of clinical cases and perspectives to screen and follow-up active and latent chronic infections. Transpl Infect Dis. 2017 Apr;19(2). DOI: 10.1111/tid.12669
482. Peixoto D, Prestes DP. Parasitic Infections of the Stem Cell Transplant Recipient and the Hematologic Malignancy Patient, Including Toxoplasmosis and Strongyloidiasis. Infect Dis Clin North Am. 2019 Jun;33(2):567-91. DOI: 10.1016/j.idc.2019.02.009
483. Michel BA, Hunder GG, Bloch DA, Calabrese LH. Hypersensitivity vasculitis and Henoch-Schönlein purpura: a comparison between the 2 disorders. J Rheumatol. 1992 May;19(5):721-8.
484. Chang HJ, Miller HL, Watkins N, Arduino MJ, Ashford DA, Midgley G, Aguero SM, Pinto-Powell R, von Reyn CF, Edwards W, McNeil MM, Jarvis WR. An epidemic of Malassezia pachydermatis in an intensive care nursery associated with colonization of health care workers' pet dogs. N Engl J Med. 1998 Mar 12;338(11):706-11. DOI: 10.1056/NEJM199803123381102
485. Bundeszentrale für gesundheitliche Aufklärung (BZgA). Hygiene und Tiere. [cited 2020 Nov 01]. Available from: https://www.infektionsschutz.de/hygienetipps/hygiene-und-tiere.html
486. Institut für Hygiene und Öffentliche Gesundheit der Universität Bonn. Hygiene-Tipps für Kids – Umgang mit Tieren. Bonn:Institut für Hygiene und Öffentliche Gesundheit (IHPH); 2007. [cited 2020 Nov 01]. Available from: https://hygiene-tipps-fuer-kids.de/files/download/pdf/Elternseiten/3_6_TiereMerkblatt.pdf
